# How Are the Flower Structure and Nectar Composition of the Generalistic Orchid *Neottia ovata* Adapted to a Wide Range of Pollinators?

**DOI:** 10.3390/ijms22042214

**Published:** 2021-02-23

**Authors:** Emilia Brzosko, Andrzej Bajguz, Magdalena Chmur, Justyna Burzyńska, Edyta Jermakowicz, Paweł Mirski, Piotr Zieliński

**Affiliations:** Faculty of Biology, University of Bialystok, Ciolkowskiego 1J, 15-245 Bialystok, Poland; m.chmur@uwb.edu.pl (M.C.); j.burzynska@uwb.edu.pl (J.B.); edytabot@uwb.edu.pl (E.J.); p.mirski@uwb.edu.pl (P.M.); p.zielinski@uwb.edu.pl (P.Z.)

**Keywords:** amino acids, female reproductive success, pollinaria removal, natural selection, orchids, plant-pollinator interactions, sugars

## Abstract

Plant-pollinator interactions significantly influence reproductive success (RS) and drive the evolution of pollination syndromes. In the context of RS, mainly the role of flower morphology is touched. The importance of nectar properties is less studied, despite its significance in pollination effectiveness. Therefore, the aim of this study was to test selection on flower morphology and nectar chemistry in the generalistic orchid *Neottia ovata*. In 2019–2020, we measured three floral displays and six flower traits, pollinaria removal (PR), female reproductive success (FRS), and determined the soil properties. The sugars and amino acids (AAs) were analyzed using the HPLC method. Data were analyzed using multiple statistical methods (boxplots, ternary plot, one-way ANOVA, Kruskal-Wallis test, and PCA). Variation of flower structure and nectar chemistry and their weak correlation with RS confirms the generalistic character of *N. ovata*. In particular populations, different traits were under selection. PR was high and similar in all populations in both years, while FRS was lower and varied among populations. Nectar was dominated by glucose, fructose, and included 28 AAs (Ala and Glu have the highest content). Sugars and AAs influenced mainly FRS. Among soil parameters, carbon and carbon:nitrogen ratio seems to be the most important in shaping flower structure and nectar chemistry.

## 1. Introduction

Plants dependent on animals in the pollination process evolved different strategies to attract pollinators, thereby increasing reproductive success. The main parts of these strategies are flower traits (the size, shape, color, scent, and nectar) adapted to a given pollinator or their whole group. Pollinator-mediated selection on floral traits is well documented, and adaptation of plants to the most effective pollinators drives the evolution of pollination syndromes [1]. The flagship example of the unusual diversity of flowers and equally differentiated pollination mechanisms is Orchidaceae, which is one of the biggest families among flowering plants [2]. About one-third of its representatives deceive pollinators through sexual or food deception [2,3,4]. Other groups of orchids reward pollinators in a different way, producing oils, nectar, resin, wax, and fragrances [5,6]. Among rewards offered by orchids, nectar is the most effective [2,7,8]. Fruiting in nectariferous orchids is significantly higher than in nectarless [2,8]. Although nectariferous orchids constitute a large part of the family, and the role of nectar in highly effective pollination is indisputable, information on its chemical composition in Orchidaceae is very scarce. Moreover, many data derive from studies using less sensitive methods in comparison to those applied recently. Importantly, more data on nectar chemistry provide results of studies on plants from other families [9,10,11,12,13,14,15], but they often focus on cultivars and the feeding needs of their pollinators, mainly bees.

Although available data document a great variability of nectar properties at different levels (species, population, and even individual), some patterns are outlined. In flower nectar, three main sugar components dominate, i.e., sucrose, glucose, and fructose, with different ratios between them. Nectar of the majority species is sucrose dominated [12,13,16], but some papers document domination of hexoses over sucrose [14,17,18,19]. The concentration of sugars also shows a great variation (from about 7–70%, [20,21]) and is connected with pollinator types [9,10,22,23], especially with the adaptation of their mouthparts to use nectar of a given viscosity. For example, bees prefer the highest concentration of sugars in nectar (35% on average), while bats and hawkmoths can suck nectar with a 17–19% concentration of sugars [23,24,25,26]. In orchids, nectar sugar concentrations range from a few to about 50% [17,18,27,28,29,30]. The preferences of pollinators also concern other components of nectar: amino acids (AAs). They are present in nectar at a lower amount than sugars but play a significant role as a source of nutrition and in attracting pollinators, thereby affecting reproductive success and survival of nectar-feeding animals [14,16,31,32,33]. Some authors suggest that taste function is even more important than a nutritive value [34,35]. Nectar of plants adapted to pollination by butterflies is characterized by high AA concentration, while those pollinated by birds or flies are characterized by their lower concentration [34]. In the nectar of different species, some AAs dominate, and others are present in low concentrations or are absent [17,28,36,37].

Apart from nectar quantity and quality, its accessibility also influences plant-pollinator interaction, thereby affects plant reproductive success. If nectar is secreted inside the corolla or in a spur, it is protected against evaporation and is available for specific, restricted groups of pollinators. On the other hand, exposed nectar may be collected by pollinators representing different morphological and ecological types and is more vulnerable to evaporation and robbery [38]. Moreover, nectar in flowers with concealed nectaries tends to be dominated by sucrose, while in more open flowers, it is dominated by glucose and fructose [29,39].

In papers dedicated to plant-pollinator interaction, the role of flower structure in attracting pollinators was studied more often than nectar properties [40,41,42,43]. In particular, phenotypic selection and its dependence on the mutual match between pollinator and flower traits are well documented [44]. This match is one of the most important evolutionary mechanisms [2,4] and is an effect of the potential for adaptation to the local partners. Many researchers have shown that pollinators act as selection agents on floral morphology and contribute to plant fitness [45,46,47]. Van der Niet, et al. [46] stated that when pollinators’ fitness is strongly influenced by an ability to access the reward in flowers of a given species, the adaptation of pollinators to flowers, rather than flowers to pollinators, takes place. In the case of plants, in which flowers are arranged as the inflorescences, floral display (the length of inflorescence and number of flowers) may also contribute to reproductive success. Plants with larger inflorescences often set more fruit, due to attracting more pollinators, which visit more flowers on larger inflorescences [48,49,50,51,52]. However, in cases in which larger inflorescences suffer from factors that decrease fitness, such as a higher probability of geitonogamy or intense herbivore activity, smaller inflorescences are favored by natural selection [52,53,54].

Both floral characters and pollinator assemblages vary in space [19,41,55,56]. Variation of floral traits in the geographic range of plant species is often an adaptation to the locally most-effective pollinators, being an answer for requirements of their specific assemblages present in a given environment [14,28,46,56,57]. The shift of floral traits and pollinators assemblages in space translate into differentiated direction and strength of selection and variation of the level of reproductive success [58].

Reproductive success depends on more than an evolutionary match between plants and pollinators. Environmental factors, both biotic (co-occurring plants) and abiotic (soil resources, weather conditions), in places where populations exist may also importantly shape plant-pollinator interactions. The composition of local pollinators is strictly connected to the diversity of the plant community because more plant species accumulate a wider spectrum of resources for flower-visiting animals [59]. Plant species richness, blossom cover, and especially the presence of attractive plant species influence assemblages of pollinators and the frequency of their visits [60,61]. It seems especially important in the case of generalist plants, which depend on many species in the pollination process. On the one hand, the presence of other flowering plants may facilitate the visitation rate, and as a consequence, increase the reproductive success of a given species [62,63]. On the other hand, a higher diversity of plant species may increase competition for pollinators when species share pollinators [62,63,64,65], especially when populations of pollinators are not abundant. Competition for pollination resources can also include intraspecific competition, which may be stronger than interspecies competition, according to niche theory [66]. The richness of the plant community, and the growth and flowering of particular species, strictly depend on soil conditions. For example, David, et al. [67] found that a high level of N in soil and a low pH decrease species diversity and the abundance of nectariferous plants. In effect, nectar and pollen resources decline, causing a decrease in pollinators’ assemblages [68]. Soil properties also shape other plant traits, which influence the level of reproductive success, e.g., the flowering [69] or quantity and quality of nectar [27].

Due to the unusual richness of orchids’ flowers and the wide variation of relations with pollinators, orchids are often considered a model system to study plant-pollinator interactions and evolutionary processes. The majority of orchid species are specialists and are connected to only one pollinator species (67% of all orchids; [70]) or a single functional group [71,72,73,74]. Others are generalists, and a wide range of animals may pollinate them. For example, *Epipactis palustris* is pollinated by more than 100 species [75], and in *Neottia ovata* almost 300 different species were noted as visitors, with about 50 species carrying pollinia [76]. Specialist orchids are more frequent objects of studies on selection/coevolution between plant and pollinators than generalists. Therefore, it seems interesting to choose the generalist orchid *N. ovata* as a model species to test in which way flower traits are adapted to pollination by a wide range of pollinators. *N. ovata* was the object of studies on pollination mechanism [76,77,78], demographic processes [79,80,81], genetic variation [82,83], and flower anatomy [84]. So far, there are no published data on nectar composition and floral structure in this orchid and their role in the effectiveness of reproduction. Therefore, the main aim of our study was to determine the floral traits of the generalist orchid *N. ovata* and to test for selection on floral morphology and nectar chemistry in populations existing in different habitats. Such studies enrich knowledge about evolutionary factors and processes that underlie the generalization or specialization and consequences at the population and species levels.

## 2. Results

### 2.1. Floral Display

We found statistically significant differences between populations in the height of the flowering shoots in both years (F = 9.390/3.422, *p* < 0.0001/0.01), while the inflorescence length differed only in 2019 (F = 14.740, *p* < 0.0001), and the number of flowers per inflorescence in 2020 (F = 2.510, *p* < 0.05) (Table 1). The highest shoots and the longest inflorescences were noted in TUR in both years. The number of flowers developed on *N. ovata* shoots was the lowest in ZAB1 in 2019 and in ZAB2 in 2020. Higher values of floral display traits were found in 2020 in four out of five cases, where statistically significant differences between years were noted. Soil parameters did not influence floral display traits [Personal communication].

### 2.2. Flower Structure

All measured flower traits were differentiated between *N. ovata* populations in 2019, while in 2020 populations differed only in labellum length and width as well as in cavity length and width (Table 1). The width of the flower was the lowest in OPA in both years and the highest in ZAB2, POG, and SKA. Labellum length was the most intra-population differentiated flower trait (CV to 1.9); it also showed the largest variability between years (in 5 among 7 populations, significant differences were noted). The labellum was the widest in ZAB1 and ZAB2 in 2019 and in OPA and POG in 2020. The size of the groove with nectar along the labellum was the shortest in OPA and TUR populations, and in the remaining cases in both years, the values of this trait reached about or even above 4 mm. The minimal and maximal values of size of the cavity with nectar were noted in different populations in both years (Table 1). In 11 out of 14 cases, where year-to-year statistically significant changes were noted, values of floral traits were higher in 2020. In populations with a higher concentration of P in the soil (Table 2), we observed correlated with shorter labellum (r_s_ = −0.71) and groove length (r_s_ = −0.83). On the other hand, in populations where a higher C:P ratio was noted (Table 2), labellum length was longer (r_s_ = 0.86).

### 2.3. Nectar Chemistry

#### 2.3.1. Sugars

**Figure 1:**
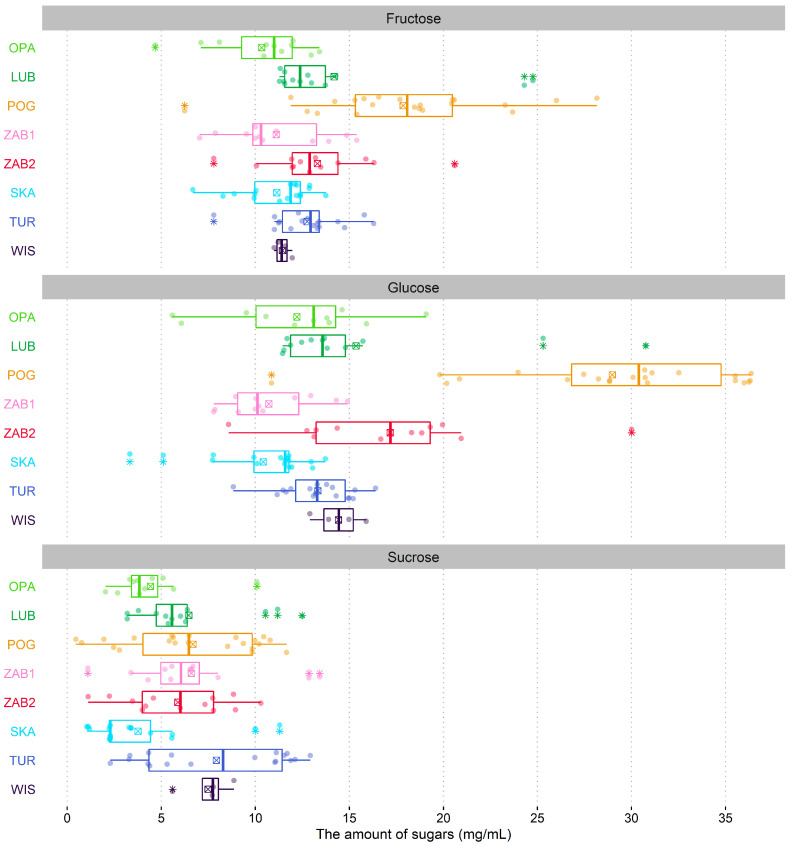
Boxplots of sugar amounts for *Neottia ovata* populations. Colored dots are individual samples. The crossed square shows the mean. The lower and upper hinges correspond to the lower (Q1) and upper (Q3) quartiles. Thus box length shows the interquartile range (IQR). The thicker line inside boxes corresponds to the median. The lower whisker extends from the hinge to the smallest value at most Q1 − 1.5 × IQR of the hinge. The upper whisker extends from the hinge to the largest value no further than Q3 + 1.5 × IQR. Data beyond the end of the whiskers, indicated with an asterisk symbol, are outliers.

In *N. ovata* nectar, three main sugars, i.e., glucose, fructose, and sucrose, were detected (Table 3). Generally, in POG, the highest concentration of all three sugars was reported (Q_3_ = 192.96 µM for glucose, 113.68 for µM fructose, and 28.75 µM for sucrose). Other populations had either significantly lower or statistically equal concentration of sugars (in terms of mean and median). *N. ovata* nectar was dominated by glucose and fructose — sucrose concentration was about 3–5 times lower than the other two sugars. Distribution of individual sugar amounts significantly varied between populations (PermANOVA, F = 5.862, R^2^ = 0.277, *p* < 0.001) (Table 3, Figure 1).

The amount of sugars varied between populations (F = 16.294, *p* < 0.001) and ranged from 25.33 ± 6.79 mg/mL and 25.50 ± 8.79 mg/mL in SKA and OPA populations to 53.51 ± 12.90 mg/mL in POG (Table 4, Figure 1). Nectar was dominated by hexoses—the sucrose:hexoses ratio shaped from 0.14 (POG) to 0.30 (ZAB1, TUR, and WIS) and varied significantly between populations (F = 5.897, *p* < 0.001) (Table 4). Populations also differed in the fructose:glucose ratio (F = 19.011, *p* < 0.001), which was close to 1 in four populations (LUB, ZAB1, SKA, and TUR). The sum of sugars as well as the amount of both hexoses positively correlated with the C:N ratio (r_s_ = 0.83 in all cases), while the N:P ratio negatively correlated with the sum of sugar (r_s_ = −0.71) and fructose content (r_s_ = −0.71).

#### 2.3.2. Amino Acids

The content of all AAs differed between *N. ovata* populations (PermANOVA, F = 8.228, R^2^ = 0.474, *p* < 0.001) and was the lowest in OPA and SKA, and the highest in POG (about 3–8 times higher than in other populations (16,662.1 ± 655.4 μg/mL, Table 5). The content of non-proteogenic AAs was also the highest in POG (735.1 ± 54.2 μg/mL). The percentage of this group of AAs differed between populations (F = 4.525, *p* < 0.001) and was the highest in OPA and SKA (about 10%) and the lowest in POG and LUB (a little above 4%, Table 5).

Among all *N. ovata* populations, 28 AAs were detected (20 proteogenic and eight non-proteogenic), with the lowest detected in populations from SLP (TUR-25 and WIS-26). Twenty-six AAs among 28 were present in all populations (Table 5). In some populations, β-Ala and Nva were absent. In each population, five non-proteogenic AAs were found. The highest participation in all populations had Ala (12.4–19.3%) and Glu (12.4–17.3%), and nine others (Leu, Gln, Asp, Asn, Cys, Pro, Val, Ser, and GABA) were noted with 5–10% frequency, although most of them reached such a frequency only in some populations (Table 5, Figure S1). Populations differed in the ratio of sugars to AAs—from 27.8 in POG to 94.6 in SKA (Table 6).

**Figure 2:**
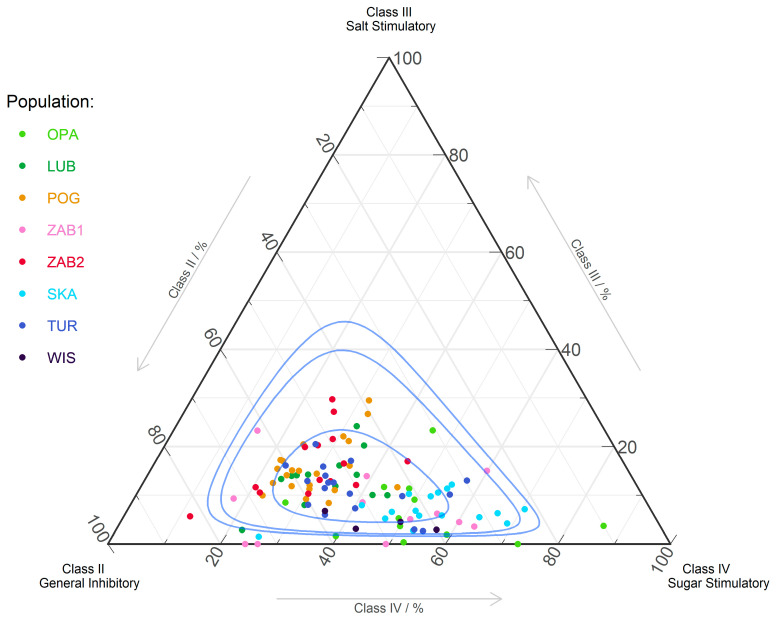
Ternary plot of amino acid classes for *Neottia ovata* populations: II (Asp, Glu, His, Arg, Lys), III (Hyp, Pro), and IV (Val, Met, Trp, Phe, Ile, Leu). Blue lines show 50%, 90%, and 95% confidence intervals via the Mahalanobis Distance and use of the Log-Ratio Transformation. The first class of AAs (Asn, Gln, Ala, Cys, Gly, Ser, Thr, Tyr) does not affect the chemoreceptors of fly (data not shown).

Amino acids responsible for nectar taste were divided into four classes. Possible simulation of insect chemoreceptors by AAs in nectars have AAs from classes II (chemoreceptor inhibitors), III (stimulation of salt cells), and IV (stimulation of sugar cells). The percentage share of class II ranged between 10.0–82.5%, while that for class III ranged between 0–19.7% and that for class IV ranged between 11.8–86.3%; the mean percentage shares were 49.9%, 11.19%, and 39.0% for classes II-IV, respectively. Fifty percent of samples had a percentage share in the range of app. 35–78% for class II, 10–22% for class III, and 22–58% for class IV (Figure 2).

**Figure 3:**
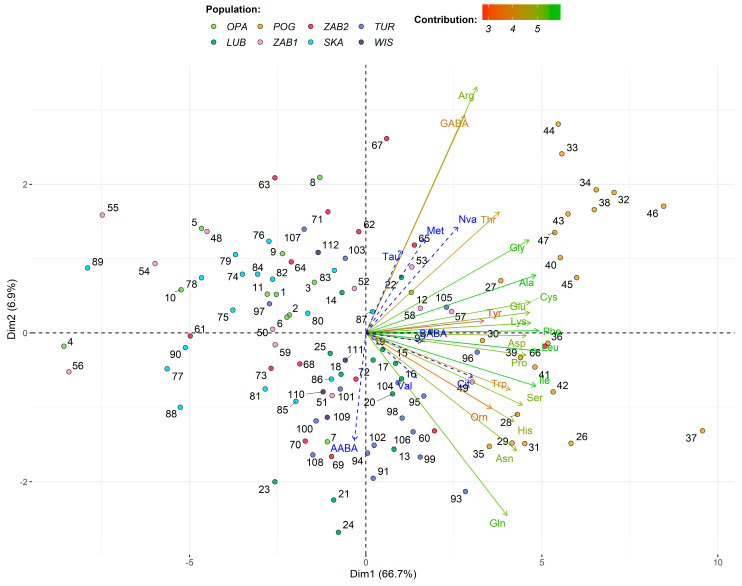
Biplot of amino acid profiles for *Neottia ovata* populations, showing the first two dimensions/factors (Dim1-2) of PCA that together explain 73.5% of the variance. Biplot vectors indicate the strength and direction of factor loading for the first two factors. Vectors of supplementary variables are in blue. Individuals (populations) are color-coded and labeled with a number corresponding to Id used in Table S3.

PCA (especially when considering only Dim1) and UMAP gave similar results in clustering individual samples and studying the underlying relations of AAs. However, it should be noted that, contrary to PCA, in the UMAP model the size of clusters relative to each other is essentially meaningless, and the distances between clusters are likely to be meaningless (Figure 3 and Figure S4). Positive scores for the first principal component (Dim1) generally indicate higher values of Gly, Ala, Cys, Glu, Lys, Phe, Asp, Leu, Pro, Ile, Ser, Trp, Cit, and BABA than mean values. Moreover, positive scores for the second principal component (Dim2) indicate values for GABA, Arg, Tau, Met, Nva, and Thr that are higher than mean values for all the populations, while a negative score shows higher values of AABA, Val, and Gln (Figure 3). The AAs show a very good quality of representation on the created model. POG population is the unique one because it has the largest amount of AAs, but it is differentiated by the levels of, e.g., AABA, Val, Tau, GABA, Arg, and Met. SKA population is very similar to OPA, while TUR is similar to LUB and possibly WIS. Samples of ZAB1 and ZAB2 populations vary; some of them are similar to SKA, OPA, and WIS (e.g., 48, 54, 55, and 83), but others to TUR (e.g., 60, 69, 70, 72, 85, and 86) and even POG (e.g., 49 and 66).

No correlation between soil parameters and the total amount of AAs was found, but the concentration of some AAs was correlated with soil properties. Different soil parameters were correlated with the amount and percentage of different AAs, although negative correlations dominated. Among soil traits, the concentration of C and the C:N ratio were most often correlated with AAs in *N. ovata* nectar (data not shown).

### 2.4. Reproductive Success

The pollinaria removal (PR) in *N. ovata* populations was shaped at a high level—above 90% in all populations in 2019, and from 81.6% (TUR) to 95% (ZAB1) in 2020 (Table 7). No statistically significant differences between populations in PR (F = 1.318, *p* = 0.28 in 2019 and F = 0.628, *p* = 0.71 in 2020) were found. Fruiting was more differentiated in both years (F = 15.430 in 2019 and F = 10.971 in 2020, *p* < 0.001) and significantly lower than PR, excluding LUB in 2019, where female reproductive success (FRS) was slightly higher than PR. In 2019, the ratio of flowers that developed into fruits was lowest in TUR and SKA populations (40.7% and 44.4%, respectively), while it was the highest in LUB (93.7%). In 2020, in most cases, the level of fruiting was lower and ranged from 14.9% (SKA) to 86.8% (POG). The efficiency of pollination varied between populations and was higher in populations from Biebrza Valley, while from other regions, the PR was about 5–9 times higher than that of FRS.

### 2.5. Factors Influencing Reproductive Success

Both PR and FRS in *N. ovata* populations were weakly correlated with flower traits. Only in five cases among 84 analyzed were statistically significant correlations between flower traits and RS parameters noted, and in particular populations, different flower traits were under selection. PR was positively correlated with flower width in TUR in 2019 (r_s_ = 0.74) and with cave width in ZAB2 in 2020 (r_s_ = 0.82), while in WIS in 2020 it was negatively correlated with groove length (r_s_ = −0.94). On the other hand, in 2020, we noted correlations between FRS and groove length in OPA (r_s_ = 0.85) as well as cave length in SKA (r_s_ = 0.76).

The statistically significant relationship between the amount of sugar and its participation and PR or FRS was noted only in three populations (ZAB1, SKA, and TUR). ZAB1 was the only population where PR, was negatively correlated with sugars: fructose (r_s_ = −0.68), sucrose (r_s_ = −0.64), and the sum of sugars (r_s_ = −0.68). The amount of glucose and the sum of sugars negatively correlated with FRS in SKA and TUR (r_s_ = −0.63 and r_s_ = −0.94, respectively). Moreover, FRS in TUR decreased with the increasing amount of sucrose and its percentage (r_s_ = −0.56 and r_s_ = −0.50, respectively) as well as the sucrose:hexose ratio (r_s_ = −0.50) and increased with the increase of fructose participation (r_s_ = 0.55).

In three populations (OPA, LUB, and WIS), AAs were not correlated with RS in any way, and in two others, only single statistically significant correlations were noted. In TUR, only the percentage of Cys positively correlated with FRS (r_s_ = 0.57), and in SKA only the percentage of Cys negatively correlated with FRS (r_s_ = −0.83) and an increased amount of Gln benefited FRS (r_s_ = 0.83). In the remaining three populations (POG, ZAB1, and ZAB2), we noted more statistically significant correlations between AAs and FRS (Table 6). Pollinia removal correlated only with Pro in POG (r_s_ = 0.53), with GABA and Cit in ZAB1 (r_s_ = −0.65 and r_s_ = 0.85, respectively), and with Val and Gly in ZAB2 (r_s_ = −0.83 and r_s_ = −0.72, respectively). Only in two populations (POG and ZAB1) did we find correlations between the amount of AAs from a particular taste group and RS. In POG, AAs from taste group IV positively correlated with PR (r_s_ = 0.56), while taste group I negatively correlated with FRS (r_s_ = −0.59). In ZAB1, the sum of AAs from taste groups I, II, and IV negatively correlated with FRS (r_s_ = −0.64, r_s_ = −0.78, and r_s_ = −0.79, respectively).

## 3. Discussion

Plants evolved different strategies to achieve reproductive success. In animal pollinated species, the level of RS depends, first of all, on the presence and abundance of pollinators [85]. Their deficiency is recognized as the main cause of low RS in orchids [2]. Assemblages of pollinators are strictly connected to the character of vegetation [60,61,86,87,88]. Plants being hosts of *N. ovata* pollinators are common (e.g., species from the Apiaceae family, and *Alnus*, *Crataegus*, *Betula*, *Salix*, *Corylus*, *Vaccinium* genera) [76] and were present, more or less frequently, in plant communities in which the studied populations exist. However, vegetation in populations and surrounding areas also showed differences, which certainly influenced insects’ assemblages. Nilsson [76] found that saw-flies, one of the most important *N. ovata* pollinators, were present only in the population near marsh vegetation. This could partially explain the higher level of RS, especially FRS, in populations that existed in BNP on mineral islands among peat bogs in comparison to others (SKA, TUR, and WIS) surrounded by a distinct type of vegetation. Other plants may also decrease RS, competing successfully for pollinators, offering them more and/or better food [62,63,64,65], especially when populations of pollinators are not abundant. Nilsson [76] found differentiation of the presence and abundance of visitors and pollinators in distinct Swedish *N. ovata* populations. Variability in insect assemblages, and their abundance was probably one of the main factors shaping the levels of RS in populations of this orchid in northeast Poland. Insect assemblages also fluctuate from year to year [76,89], which may explain the temporary variation of RS in some *N. ovata* populations.

According to Nilsson [76], *N. ovata* may be visited by almost 300 species, representing different systematic groups with a wide spectrum of body sizes, mouth apparatus, and nutritional preferences. Undoubtedly, the main role in the attraction of these insects is played by the scent bouquet, comprised of compounds that are known as general attractants of a wide range of insects [71,76]. Numerous insects capable of pollinating flowers of this orchid, together with the easily available nectar on the labellum, create a chance for a high level of RS. In the majority of populations, we observed a higher level of fruiting than those found by Brzosko [80] and Brys, et al. [79]. PR in all *N. ovata* populations in both years (always above 80% or even above 90% in many cases) suggests that a large number of insects penetrated flowers. On the other hand, FRS was more differentiated (similar to the seven-year studies of Brzosko [80]). The higher efficiency of pollination we noted in populations from Biebrza National Park, and in remaining FRS was 5–11 times lower than that of PR. This indicates that not all insects that visited flowers (even able to collect pollinia) were effective pollinators. Probably some of the visitors, especially the smallest or the weakest, may suck nectar only from the groove along the labellum and do not penetrate flowers in-depth, omitting in this way the cave at the labellum base, which decreases the probability of contact with the column. Moreover, pollinaria may be attached to different parts of the insect’s body [76], and the position of the visitor sucking nectar may sometimes be unsuitable for the collection of pollinia and/or to place them on the stigma. The low efficiency of pollination could be explained by Nilsson [76]. The author found behavioral disturbances of smaller ichneumons (dominant pollinators) if they have big loads of pollinia. Insects that do not penetrate the flowers correctly may occasionally contribute to the pollination of *N. ovata* flowers. This indicates that pollination in this species has a haphazard character. The disparity between PR and FRS indicates that pollinia are often lost, as observed by Brys, et al. [79].

Incorrect flower penetration, causing ineffective pollination, is an effect of mismatch between flower and pollinator. The mechanical fit between partners is one of the essential preconditions of successful pollination [45,46,47] and one of the most important evolutionary mechanisms [2,4,44]. Such a match is generally stronger in specialized systems [90], which confirms, for example, the results of studies on long-spurred orchids [41,42,43,51]. Our results suggest the best fit between flowers and pollinators in POG and LUB populations. PR:FRS in LUB in 2019 and in POG in 2020 was close to 1, and additionally, in LUB in 2019, FRS was higher than PR, indicating the presence of effective pollinators and their high efficiency. The high PR:FRS ratio in other populations and the relatively low FRS in some of them denote a mismatch between flowers and visitors and, as a result, a larger loss of pollinia. Weak correlations between flower traits and PR and FRS (five cases among 84 analyzed in both years) confirm this mismatch. In these single cases, distinct flower traits were under selection in particular populations. Nevertheless, four among five flower traits correlated with PR or FRS concerned the sizes of structures (groove and cage), in which nectar is secreted and accumulated. Because we supposed that groove and cavity sizes are the measures of nectar quantity, it could indicate that the amount of nectar is the most important trait influencing RS in *N. ovata*. We expected that the labellum in this orchid, as a landing platform and flower part, should be adapted to pollinators’ sizes. Although we did not find an influence of labellum on RS parameters, its length was the most differentiated flower structure between populations, which suggests that it reacts on local insect assemblages. The disparity between the level of PR and FRS in most populations, probably being an effect of structural mismatch, may be explained through the great variation in body sizes of *N. ovata* visitors and the differentiation of their behaviors as nectar consumers. Even the main group of pollinators of this species (ichneumonids) includes representatives with a wide range of sizes [76]. In populations with lower FRS, these were probably predominant insects that more accidentally remove pollinia and less often place them on stigmas of other flowers. Because their main dietary sources are connected to other plant species, and *N. ovata* is a marginal part of the food (if only because of small population sizes), they do not need to adapt to its flowers. This suggests that the level of *N. ovata* RS depends on accompanying plant species, their diversity, and their abundance. Contrary to Brys, et al. [79] results, we did not find an influence of floral display on RS in *N. ovata*.

In nectariferous plants, the amount and composition of nectar are known to affect plant-pollinator interactions [9,10,12,13,16,19,22,23,27,28,34]. Our studies document that *N. ovata* is characterized by exceptionally diluted nectar with the lowest sugar concentration among orchids [17,18,27,28,29,30]. To our knowledge, these values are comparable only to the concentration of nectar used by some hummingbirds [26,91]. The relatively low sugar concentration was noted for plants pollinated by moths and flies [17,23,24,26,92,93], and only fly-pollinated species have extremely low volume and sugar concentration but high amino acids and hexose content [12,34]. Some fly species are also known to pollinate *N. ovata* [76]. The nectar of *N. ovata* is dominated by hexoses, which is in agreement with the statements of Gottsberger, et al. [39] and Pais, et al. [29] that nectar in flowers with concealed nectaries tends to be dominated by sucrose, while in more open flowers by glucose and fructose. Hexose solution has a higher osmolarity, and therefore lower evaporation rates, than sucrose solution, which can explain the high proportion of hexoses in shallow flowers [12]. However, the prevalence of hexoses was noted in the nectar of some long-spurred orchids [17,18]. On the other hand, contrary to our results, Galetto, et al. [18], studying nectar in five orchid species, found that nectar located in the spurs in two *Habenaria* species was copious and less concentrated (<20%), while in species in which nectar was accumulated in the basal lateral parts of the labellum, it was more concentrated (ca. 50%). Our results are in accordance with the studies of Johnson and Nicolson [94], who documented a clear distinction between nectar sucrose content of specialized (40–60%) and generalized (0–5%) bird-pollinated species. Nonspecialized insects, i.e., syrphids, flies, and beetles (insects from these groups are *N. ovata* pollinators) [76] preferred monosaccharide nectar of plants from phryganic communities [22]. Hexose-rich nectar, which is taken up more easily than sucrose, may be an adaptation and advantage for attracting a wide range of nonspecialized pollinators. It is worth noting that low sucrose nectar is also characteristic for species from the Apiaceae family [22,95,96], which are pollinated by the same systematic groups of insects as *N. ovata*. The lack of influence of nectar sugars on RS in five populations confirms that these nectar traits are not aimed at any of the pollinator group. Moreover, our results could suggest that insects operated in the three remaining populations did not prefer nectar sugar composition in nectar offered by *N. ovata*. With the exception of the TUR population, in which fructose participation benefited FRS, in the remaining cases, statistically significant negative correlations were noted. In studies on two *Platanthera* species, we also noted positive selection only on fructose content [17]. To amount the preferences of insects, experiments should be performed. Hexoses, and especially fructose, are preferred by some pollinators due to their lower viscosity, enabling easier absorption [25]. Heil [31] documented that some ants (often observed on *N. ovata* in our studies and by Nilsson [76]) even preferred sucrose-free nectar because they are not able to assimilate this sugar due to lack of invertase. Sucrose-rich nectar may be toxic for some generalists. All the above-mentioned results, at least partially, explain the dominance of hexoses and the high fructose:glucose ratio in the majority of *N. ovata* populations. The concentration of sugars in nectar and the sucrose:hexose ratio also depend on water availability [22]. *N. ovata* populations exist in relatively wet places, and heavy rainfall in 2020 might additionally decrease sugar concentration.

*N. ovata* nectar is rich in amino acids, we noted 28 distinct AAs (20 proteogenic and eight non-proteogenic), and 26 were common for all populations. In the nectar of specialist orchids from the *Platanthera* genus, 23 AAs were found in total, from nine to 20 in each population [17]. Moreover, the nectar of other orchids was composed of the lower number of AAs—20 in *Gymnadenia conopsea* [28] and 17 in *Limodorum abortivum* and *Epipactis atropurpurea* [29]. In three populations (OPA, LUB, and WIS), no relationship between RS and AAs was found, while in two others (TUR and SKA), only single statistically significant correlations were noted—the percentage of Cys positively influenced FRS in TUR, while in SKA negatively influenced FRS. In the last population, an increased amount of Gln increased FRS. In the remaining three populations (POG, ZAB1, and ZAB2) we noted a larger influence of AAs on RS; it concerned mainly FRS and almost all of the correlations were negative. Pollinia removal depended only on Pro in POG, on GABA and Cit in ZAB1, and on Val and Gly in ZAB2. The most abundant in all *N. ovata* populations were Ala and Glu, but they weakly affected RS, having only a negative influence on FRS (Glu in SKA and POG, and Ala in ZAB1 and POG). Other AAs with a relatively high amount in *N. ovata* nectar were Asp, Cys, Gly, Thr, Asn, Gln, Ile, Phe, and Pro among the group of proteogenic AAs, and Orn and GABA among non-proteogenic ones.

AAs in floral nectar are important for the survival of nectar-feeding animals [14,16,32,33,97], although the role of particular AAs is poorly explained. It is known, for example, that one among the most abundant AAs in *N. ovata* nectar (Ala) influences insects’ growth, while the second (Glu) affects pollinators’ behavior [28] similarly to Leu and Met [12]. On the other hand, Venjakob, et al. [61] found that Ala and Gly may deter honeybees. In the case of *N. ovata*, the second function of Ala is more probable, as we found a negative correlation of this AA with RS. One of the most common AAs in plant nectar is Pro, which rewards pollinators and acts as a propellant for the lift phase of the flight [98,99]. It triggers the normal insects’ salt-receptor neurons, which initiates feeding [19,23,97]. Its accumulation is also interpreted as a plant’s answer to stress factors [98]. Pro was present in all populations studied with a quite high amount, but only in POG did it positively correlated with PR, and in ZAB2 with FRS. Two other AAs (Asp and Thr), which belong to the most abundant AAs in *N. ovata* nectar, seem negatively correlated with FRS in ZAB1, and are known as general repellents [14]. Moreover, Glu, Leu, and Met play a potential role in parasitoid rejection [12]. One of the two most abundant non-proteogenic AAs in *N. ovata* nectar, GABA, influences the insect nervous system and muscle activity [11,100]. Its higher amount was connected with a decrease in both PR and FRS in ZAB1. An interesting result was observed in POG, where a higher amount of this AA was negatively correlated with fruiting, while its percentage was positively correlated with FRS. This indicates that not only the amount of a particular AA, but also relationships between them, may be important in shaping plant-pollinator interaction. BABA, although a less common AA in nature than GABA, was present in all *N. ovata* populations with a relatively high amount. It contributes to protecting plants from pathogens [101,102]. One of the important nectar traits is its taste, which attracts or discourages visitors and depends on some AAs [27,28,103]. AA compositions influence pollinator taste perception and pollinating behavior through specific neurological or phago-stimulating pathways [12]. Some authors suggest that the taste function is even more important than the nutritive value [34,35]. We observed a potential influence of the amount of AAs from a particular taste group only in POG and ZAB1. The positive correlation between taste group IV and PR was noted only in POG. In the remaining cases, nectar taste could shape FRS, always in a negative way. This may indicate that insects present in the majority of *N. ovata* populations were not sensitive to nectar taste or did not prefer this taste. Similar results were obtained for other nectariferous orchids [17,27].

We found inter-population variation in flower structure and the amount of particular nectar components, similar to other studies [19,27,41,55,56,58,79]. One of the sources of this variability is differences in soils in which *N. ovata* grows. Soil properties influenced mainly nectar composition. Production of nectar is costly, even to 30% of flower costs [104]; thus, it requires adequate soil resources. Our studies suggest that more important than the participation of particular chemical elements in the soil is their proportionality. The most important in shaping nectar character were the C:N and N:P ratios. In POG, where C:N was the highest, the sum of sugars was 1.5–2 times higher, and the amount of AAs was three to even almost eight times higher than in other populations. The increase of the C:N ratio in soil caused a higher sum of sugars, glucose, and fructose as well as some AAs (Asp, Glu, Asn, Ser, Trp, and His). Simultaneously, the increase in the same soil characteristic had a negative effect on the percentage of Gly, Thr, Tau, Met, and GABA. A higher N:P ratio negatively influenced the total amount of sugars and fructose and the percentage of Glu and Tyr, while it increased the percentage of Cys and Ile. The importance of soil traits for nectar traits or plant condition was noted by other authors [27,28,34,39,69].

It should be noted that the other factors, such as weather conditions, may shape plant properties and pollinators’ assemblages and their activity [12,81,96]. The weather condition in our studies differed between seasons: 2020 was rainier than 2019. It could cause that higher values characterized some plant parameters in the second year of the study. In 19 out of 63 cases, we noted statistically significant changes between years; in 15 cases, we observed an increase of these values (3 cases of floral display and 11 of flower traits). On the other hand, in 2020, in most cases, the level of fruiting was lower. The explanation of year-to-year changes of plant traits due to weather should be undertaken with caution because only some traits and only in some populations differed between seasons. Moreover, in neighboring populations in the Biebrza National Park, the same traits often changed in opposite directions from year to year. It can indicate the greater role of other factors than the weather in these changes.

In our study, we tried to answer the question: In which way is *N. ovata* adapted to a wide range of pollinators? As a generalist with reference to pollinators, *N. ovata* depends on an exceptionally high number of insects in the pollination process—almost 300 species of visitors, among which at least 50 species attached pollinia [76]. How can the demands of such a wide range of insects, differing in sizes, mouth apparatus, nutritional needs, and behaviors, be met? The answer is simple: The plants’ offer should also be wide. In the case of *N. ovata*, this rich offer includes the wide range of nectar components (e.g., a large number of AAs) and differentiation of their amounts as well as the variability of flower structures. This indicates that this species did not evolve flower traits, which filter flower visitors; thus, they are not dedicated to a certain group of pollinators. Generally, our results fit the generalistic character of *N. ovata*, but the level of generalization at the species level seems to be higher than at the population level. The lack of, or poorly matched, interactions between flower structure and nectar chemistry and the levels of RS in *N. ovata* populations from northeast Poland confirm this statement. This finding is in agreement with the results of studies on other generalists [90]. Jacquemyn and Brys [105] found that a large variation in flower traits in *Orchis purpurea* populations is maintained by the lack of strong selection pressures on these traits. Differentiation of flower traits enables pollination by whatever flower visitors have a suitable size and appropriate behavior. The probability that whatever species among almost 300 *N. ovata* visitors will serve as an effective pollinator is quite high. However, the variation of FRS among populations suggests that despite the high number of potential pollinators of this species, their abundance in particular populations was extremely differentiated. A low level of fruiting in some populations and a high ratio of PR:FRS (especially in SKA) indicates pollinators’ deficiency. In SKA, the problem with pollinators is deeper due to anthropogenic impact. It exists in disturbed and fragmented habitats in a restricted area, less abundant in plants, being hosts of *N. ovata* visitors. Significantly lower PR in such populations may reflect unsuitable conditions for insects. The high levels of fruiting in populations from Biebrza National Park resulted from the relatively unchanged environment in this area. Natural habitats are suitable for many plant species connected to insects pollinating *N. ovata* flowers. The higher RS in populations from BNP could also be a result of their larger sizes in comparison to others. The minimum population size is often required to attract sufficient pollinators. This assumption is supported by the results of Brys, et al. [79] study, which found a significant relationship between RS and population size in *N. ovata*.

Our results contribute to the knowledge about the reproductive strategy of *N. ovata* and fit into studies that explain the causes and consequences of generalization in plants. However, in the course of this study, new questions arose, which required further analysis. For example, why does this orchid invest so many resources into nectar production if it is not an effective allurement of insects, as in other nectariferous species, and which nectar components are the most important for its fitness? The yellow-green color of *N. ovata* flowers does not attract pollinators because it does not contrast with the surrounding vegetation. The color purity is typical of many generalist insect-pollinated plants [71]. In such cases, other flower traits (odor and nectar) play a key role. Floral nectar (its concentration and composition) is rarely detectable by a pollinator at a distance [11]. The fragrance is a key floral attractant for most wasps and beetles—the insect groups that are pollinators *of N. ovata*—and also other generalistic plants accompanying this orchid; also, they often possess the same odor compounds [76,95,96,106]. A probable scenario is that at the first step insects are attracted by the fragrance emitted by *N. ovata* flowers, which can explain the high level of PR; however, after probing nectar, which seems tasteless to the visitors, they do not further penetrate the flowers, thus causing a decrease in FRS. If so, why does *N. ovata* produce such ineffectual rewards? The answer partially explains Johnson and Hobbhahn [71] hypothesis that generalist pollination in orchids comes with high reproductive costs. According to the authors, these higher costs also include pollinia losses and inefficiency of pollination, characteristic of most orchids.

Supposing that, in the evolution process, all acts are intentional/on purpose, these high costs also contribute to *N. ovata* fitness. Each of the flower traits developed in this orchid is equally important in shaping RS, even those that seem to be negligible in our studies. They may operate side by side as “comprehensive consumer infinity” for pollinators. It seems that the wide flower variability and complexity of their action is the advantage of this species, which enables the maintenance of populations under different environmental conditions. The results of our studies also have conservation implications; protection of this orchid requires the protection of its wide spectrum of insect partners and their hosts and, thus, the entire habitats in which *N. ovata* exists.

## 4. Materials and Methods

### 4.1. Study Species

*Neottia ovata* is a long-lived, shade-tolerant forest herb with a wide geographical range covering Western Europe to Eastern Siberia [107]. It usually grows on moderately dry to wet soils with a wide range of pH (pH = 5.5–7.5) [108,109]. Yellow-green flowers (15–30) develop on a flexible raceme. Flowers open and age sequentially and remain receptive for 2–3 days. Each flower possesses two pollinia attached to each other, being removed as a pair. *N. ovata* is self-compatible but has a mechanism (well-developed rostellum) to prevent self-fertilization [76,78]. Flowers emit a distinct and somewhat sweet scent and secrete nectar on the labellum [107]. Nectar is produced in the shallow cavity at the wide lip base and in a lesser quantity in a central longitudinal groove along the elongated part of the two-lobbed lip [77,84]. *N. ovata* attracts many insect species. Nilsson [76] observed 283 visitors in Swedish populations, mainly unspecialized anthophilous insects such as ichneumonids, sawflies, and beetles, among them at least 50 species (belonging to the Hymenoptera, Coleoptera, and Diptera) with attached pollinia. After landing, a visiting insect licks the nectar secreted in a groove. Following the nectar trail, the insect is guided to the lip base and the gynostemium [76,77]. Fruits become ripe at the end of June. In one capsule, 218–1774 seeds develop [78].

### 4.2. Study Area

This study was performed in eight populations of *N. ovata* in northeast Poland. Five of them were localized on mineral islands among pit bogs in the Biebrza National Park (OPA, LUB, POG, ZAB1, and ZAB2), two in the Suwałki Landscape Park (TUR and WIS), and one in Knyszyńska Forest (SKA). Studies were conducted during two years (2019 and 2020), excluding WIS, which was observed only in 2020. Populations differed in size and existed under different environmental conditions (Table 8).

### 4.3. Fieldwork and Floral Trait Measurements

Because *N. ovata* populations in northeast Poland are small and only 10–20% of the population flowers each season [80], we have started observations on 20–22 individuals (whenever available) from each population. The final sample size was in many cases lower because some shoots were damaged during flowering or before fruiting. We have quantified three floral display traits directly in the field during the peak of flowering: height of shoots, length of inflorescence, and number of flowers. Next, we collected the five lowest flowers from each inflorescence. All five were used for the evaluation of nectar composition, while two out of those were drawn randomly to measure morphological variables such as flower width, labellum length and width, length of longitudinal groove with nectar on the labellum, and length and width of the cavity with nectar at the base of the labellum. The size of the groove and cavity were considered measures of nectar quantity. Samples from all populations were collected during three days under sunny weather. The measures were taken using an opto-digital microscope DSX110 (Olympus Life Science, Waltham, MA, USA) in the Laboratory of Insect Evolutionary Biology and Ecology, Faculty of Biology, University of Bialystok.

To assess the level of reproductive success (RS), we marked shoots and counted the number of flowers per inflorescence in full blooming. During the maturation of capsules, FRS and PR were quantified. FRS was evaluated as the proportion of developed fruits to the number of flowers on the inflorescence and was given in percent. PR was determined in the percent (PR to the total number of pollinaria for each inflorescence). We also evaluated the efficiency of pollination as the ratio of PR:FRS; the higher the index, the lower the pollination efficiency within a population.

### 4.4. Soil Analysis

Three soil samples were taken from each population at a depth of 5–10 cm. Samples were dried at room temperature, ground, and sieved (1 mm). Two types of pH were measured with a Hach-Lange pH meter (Hach Company, Loveland, CO, USA) in a 1:2.5 soil water mixture and 1:2 soil KCl solution (1 M) mixture [110]. About 25–50 mg of soil was used for total soil organic carbon analysis by dry combustion at 900 °C using the TOC-A Shimadzu analyzer with SSM-5000A combustion module (Shimadzu Corporation, Kyoto, Japan). About 0.5–1 g of dry soil samples were treated with 10 mL of10% HCl and connected to the gas-tight Scheibler apparatus according to the CO_2_ volumetric carbonates analysis method [111]. Total nitrogen content was measured with the Spectroquant nitrogen cell test (Merck KGaA, Darmstadt, Germany) according to Koroleff’s method [112]. Soil samples were treated with an oxidizing agent in a thermoreactor, then acidified with sulphuric and phosphoric acid. Nitrogen was measured photometrically with 2,6-dimethylphenol (DMP). Total phosphorus content was measured by perchloric acid digestion followed by the molybdate photometrical test. The absorbance was measured with a spectrofluorometer SpectraMax M2 (Molecular Devices, Sunnyvale, CA, USA).

### 4.5. Nectar Analysis

#### 4.5.1. Nectar Isolation

Nectar chemistry was studied in 2020. Five flowers per individual were used for nectar analyses. Our preliminary analyses showed that the nectar amount from the lower number of flowers was not enough to correct the detection of nectar components. The flower nectar isolation was performed using a water washing method [113]. Five flowers per sample were placed into the 2 mL Eppendorf tube containing 1 mL of distilled water and shaken in a laboratory thermomixer (120 rpm, 21 °C, 45 min; Eppendorf Corporate, Hamburg, Germany) for the nectar efflux. Then, the flowers were removed from the tubes, and the mixture of water with nectar was evaporated to dryness by centrifugal vacuum concentrator (45 °C, Eppendorf Concentrator Plus, Eppendorf Corporate, Hamburg, Germany). The obtained pellet was dissolved in 20 µL of distilled water, then transferred into the centrifuge tube with a filter and centrifuged to remove impurities (9000× *g*, 5 min; MPW-55, MPW Med. Instruments, Gliwice, Poland). The purged extract was collected in a glass vial with a 250 µL insert with polymer feet.

#### 4.5.2. Sugar and Amino Acid Determination

Determination and quantification of sugars and AAs were performed using the high-performance liquid chromatography (HPLC) method. An Agilent 1260 Infinity Series HPLC apparat (Agilent Technologies, Inc., Santa Clara, CA, USA) with quaternary pump with an in-line vacuum degasser, thermostatted column, and refrigerated autosampler with autoinjector sample loop was used.

For sugar analysis, a ZORBAX Carbohydrate Analysis Column (4.6 mm × 250 mm, 5 µm) (Agilent Technologies, Inc., Santa Clara, CA, USA) at a temperature of 30 °C and a refractive index detector (RID) was applied. The mobile phase was a solution of acetonitrile/water (70:30, *v*/*v*) at a flow rate of 1.4 mL/min. The injection volume was 10 µL. The total time of analysis was 15 min [17].

Meanwhile, for AA detection, an automatic program of derivatization was set. Thus, the *o*-phthalaldehyde (OPA) and 9-fluorenylmethyl chloroformate (FMOC) reagents were used for the derivatization of primary and secondary AAs [17]. The Agilent Zorbax Eclipse Plus C18 (4.6 × 150 mm, 5 µm) column (Agilent Technologies, Inc., Santa Clara, CA, USA) at a temperature of 40 °C was used to separate individual AA. Detection of primary AAs was performed by a photodiode array detector (DAD) at 388 nm, while detection of secondary AAs was performed by a fluorescence detector (FMOC) with an excitation wavelength of 266 nm and an emission wavelength of 305 nm. The injection volume was 5 µL; the flow rate was 1 mL/min. Eluent A of the mobile phase was 40 mM NaH_2_PO_4_ (pH 7.8, adjusted by 10 M NaOH solution), while eluent B was a mixture including acetonitrile/methanol/water (45:45:10, *v*/*v*/*v*). The gradient was the following: 0–5 min, 100–90% A; 5–25 min, 90–59.5% A; 25–30 min, 59.5–37% A; 30–35 min, 37–18% A; 35–37 min, 18–0% A; 37–40 min, 0% A; and 40–43 min, 100% A.

The analytical data were integrated using the Agilent OpenLab CDS ChemStation software (Agilent Technologies, Inc., Santa Clara, CA, USA) for liquid chromatography systems. Identification of sugars and AAs was performed by comparing retention times of individual sugars and AAs in the reference vs. test solution. The concentration of these compounds was assayed based on comparisons of peak areas obtained for the samples investigated with those of the reference solutions.

### 4.6. Statistical Analysis

The R programming language/statistical environment was used to perform all statistical computations and analyses, as well as to prepare graphics and transform data for tabular representation [114,115]. The dataset of AAs and sugars were checked for equal variances and normal distribution in each of the populations with the Shapiro-Wilk test and Levene’s test [114,116], respectively (both failed for all or some of the groups/variables). Interestingly, in the dataset of the sum of sugars (glucose + fructose + sucrose) for each population were normally distributed and had homogenous variances. The dataset of floral display and flower structure in *N. ovata* populations in northeast Poland in 2019–2020 were also tested using Shapiro-Wilk and Levene’s tests with the result that data were normally distributed and had homogenous variances.

Differences among populations in floral display and flower traits were tested using one-way ANOVA (“stats” package). The influence (monotonous relation) of analyzed parameters on reproductive success (PR and FRS) was checked separately with Spearman’s correlation coefficient (r_s_) for each population. The same test was used to evaluate the influence of soil parameters on flower display, floral traits, and nectar chemistry, but in this case, correlations were made at the population level between soil characters and average values of analyzed traits.

Dataset of the sum of sugars was also subjected to one-way ANOVA followed by Tukey’s post-hoc test. Sugar and AA datasets were supplied to the Kruskal–Wallis test (to perform a non-parametric alternative to the one-way ANOVA test) followed by a pairwise Wilcoxon Rank Sum test with Benjamini-Hochberg adjustment that compared the median values of different parameters between populations [117,118,119]. Composition of sugars and AAs was tested between populations using Permutational Multivariate Analyses of Variance (PermANOVA) in “vegan” package [120]. Furthermore, a set of descriptive statistics (n, mean, standard error, quartiles) was calculated for AAs and sugars (Figure S1). For all tests, the significance level was α = 0.05. To analyze the effect of AAs on insect chemoreceptors, all identified and determined AAs were grouped into four classes [12]: I. Asn, Gln, Ala, Cys, Gly, Ser, Thr, and Tyr (no effect on the chemoreceptors of fly); II. Arg, Asp, Glu, His, and Lys (inhibition of fly chemoreceptors); III. Pro and Hyp (stimulate the salt cell); and IV. Ile, Leu, Met, Phe, Trp, and Val (ability to stimulate the sugar cell) and presented as a ternary plot [121].

Principal component analysis (PCA) was used to simplify the exploration of AAs. To build the PCA model, the “FactoMineR” package was used [122]. Data (except for β-Ala, which was present in only a few samples) were transformed using Tukey’s Ladder of Power [123] with λ that maximizes the Shapiro-Wilk’s W statistic using the “rcompanion” package [119]. Starting λ was set to −10, and ending to 10, while the interval between λ was to 0.005. Ala was scaled using y = −x^−0.06^; all other AAs were scaled using y = x^λ^ (Table S1). Two tests that indicate the suitability of the AA dataset for structure detection and reduction were performed: Bartlett’s test of sphericity [124] and the Kaiser-Mayer-Olkin test of factorial adequacy (KMO) (“psych” package [125]). The *p*-value from Bartlett’s test of sphericity was approximately equal to 0, while the calculated overall measure of sampling adequacy (MSA) from the KMO test was equal to 0.92. MSA for individual AAs ranged from 0.53 to 0.95 (Table S2). Thus, according to Kaiser [126], the MSA value is high enough to perform PCA. Unit variance scaling of the data (scale.unit = TRUE) was applied; thus, PCA was performed on a correlation matrix, rather than on a covariance matrix. Different PCA models, i.e., without and with different data transformation techniques, as well as supplementary variables, were also created and investigated. Finally, six AAs did not participate in the creation of the final PCA model. Instead, they were used as supplementary variables to help interpret the dimensions of variability. According to Cattell’s rule, two components should be selected [127], while Kaiser’s rule indicated that three components should be retained [128]. Studying the cos^2^ plot (Figure S2) led to the selection of the first two components that explain about 73.5% of the variance (Figure S3). All biplots were created using the “factoextra” package [129]. Furthermore, uniform manifold approximation and projection (UMAP) were performed on a raw AA dataset with the exclusion of β-Ala to provide an additional source for detecting sample and population similarity (Figure S4) (“umap” package [130]).

## Figures and Tables

**Table 1:** Variation of floral display and flower structure in *Neottia ovata* populations. Data show the mean ± standard deviation. Dark blue values in bold indicate statistically significant differences between years.

Population	Year	N	Shoot Height (cm)	Inflorescence Length (cm)	Number of Flowers	Cavity Length (mm)	Cavity Width (mm)	LabellumLength (mm)	Groove Length (mm)	LabellumWidth (mm)	Flower Width (mm)
OPA	2019	38	**51.02 ± 5.56**	17.75 ± 3.22	22.75 ± 7.22	0.98 ± 0.10	1.34 ± 0.11	**8.60 ± 1.11**	3.70 ± 0.60	**3.29 ± 0.45**	7.96 ± 0.95
	2020	26	**44.50 ± 8.58**	16.28 ± 4.25	28.56 ± 12.94	0.94 ± 0.09	1.41 ± 0.08	**10.05 ± 1.17**	3.94 ± 0.35	**3.88 ± 0.49**	8.21 ± 0.53
LUB	2019	38	53.84 ± 10.62	17.71 ± 5.83	24.27 ± 7.43	**1.03 ± 0.10**	1.37 ± 0.16	9.03 ± 1.01	3.99 ± 0.38	3.35 ± 0.38	8.69 ± 0.53
	2020	28	46.63 ± 13.03	17.75 ± 6.41	26.43 ± 8.04	**0.92 ± 0.11**	1.29 ± 0.10	9.63 ± 1.02	3.98 ± 0.44	3.46 ± 0.33	8.54 ± 0.74
POG	2019	44	**47.45 ± 6.53**	**14.06 ± 3.53**	26.55 ± 4.78	**0.91 ± 0.09**	1.39 ± 0.13	**9.12 ± 0.91**	4.03 ± 0.36	**3.42 ± 0.36**	8.77 ± 0.57
	2020	44	**53.31 ± 8.49**	**20.15 ± 4.14**	25.75 ± 7.56	**1.03 ± 0.11**	1.44 ± 0.12	**10.78 ± 1.17**	4.12 ± 0.38	**3.93 ± 0.46**	8.96 ± 0.60
ZAB1	2019	40	51.70 ± 9.21	19.63 ± 4.14	22.86 ± 9.13	0.97 ± 0.15	1.35 ± 0.11	8.59 ± 1.33	**4.13 ± 0.37**	3.59 ± 0.41	8.61 ± 0.81
	2020	30	52.73 ± 8.82	18.82 ± 2.33	29.25 ± 9.45	0.99 ± 0.12	1.39 ± 0.11	9.39 ± 1.26	**3.85 ± 0.32**	3.59 ± 0.34	8.39 ± 0.40
ZAB2	2019	46	52.09 ± 6.74	14.86 ± 4.15	28.38 ± 9.25	1.02 ± 0.11	**1.46 ± 0.15**	**8.67 ± 1.38**	4.14 ± 0.39	3.59 ± 0.43	8.81 ± 0.69
	2020	33	51.04 ± 8.55	17.58 ± 3.94	24.46 ± 6.42	1.03 ± 0.10	**1.35 ± 0.09**	**9.71 ± 1.11**	3.94 ± 0.42	3.48 ± 0.54	8.64 ± 0.71
TUR	2019	44	61.91 ± 8.49	24.34 ± 4.73	**27.33 ± 8.45**	1.04 ± 0.10	1.38 ± 0.07	**7.91 ± 0.92**	**3.70 ± 0.37**	**3.22 ± 0.41**	**8.30 ± 0.61**
	2020	40	59.32 ±8.50	20.00 ± 4.61	**35.78 ± 10.49**	1.07 ± 0.12	1.40 ± 0.09	**10.17 ± 0.94**	**3.98 ± 0.37**	**3.78 ± 0.47**	**8.72 ± 0.69**
WIS	2019	nd	nd	nd	nd	nd	nd	nd	nd	nd	nd
	2020	12	51.67 ± 6.55	18.25 ± 4.55	29.83 ± 11.30	1.06 ± 0.11	1.32 ± 0.05	10.92 ± 0.75	4.18 ± 0.51	3.62 ± 0.33	8.63 ± 0.75
SKA	2019	42	45.88 ± 7.09	**15.48 ± 3.92**	26.22 ± 6.08	1.05 ± 0.12	1.42 ± 0.12	**8.85 ± 1.28**	4.13 ± 0.50	3.55 ± 0.46	8.86 ± 0.60
	2020	44	46.32 ± 10.46	**19.73 ± 5.60**	25.27 ± 5.95	1.00 ± 0.09	1.38 ± 0.09	**9.96 ± 1.30**	4.04 ± 0.43	3.70 ± 0.33	8.61 ± 0.72
IPD	2019		*p* < 0.001	*p* < 0.01	NSD	*p* < 0.05	*p* < 0.05	*p* < 0.05	*p* < 0.05	*p* < 0.05	*p* < 0.05
	2020		*p* < 0.01	NSD	*p* < 0.05	*p* < 0.01	*p* < 0.01	*p* < 0.01	NSD	*p* < 0.05	NSD

nd—no data. IPD—inter-population differentiation of particular traits. N—number of analyzed flowers. NSD—nonsignificant differences.

**Table 2:** Soil parameters for *Neottia ovata* populations.

Population	% C	% N	% P	C:N	C:P	N:P	pH Water	pH KCl	CaCO_3_
OPA	14.28	0.32	0.08	44.1	185.90	4.20	7.94	7.63	7.53
LUB	16.08	0.11	0.15	150.3	110.10	0.70	8.00	7.77	12.96
POG	15.27	0.06	0.05	260.00	309.40	1.20	7.36	7.21	0
ZAB1	0.01	0.32	0.20	0.03	0.06	1.60	8.04	7.77	13.22
ZAB2	37.41	0.70	0.60	53.60	61.90	1.20	6.81	6.73	0
TUR	44.26	0.65	0.16	67.70	278.20	4.10	7.13	6.89	0
WIS	13.13	0.15	0.05	85.70	253.80	3.00	7.78	7.65	13.52
SKA	5.31	0.19	0.06	27.40	95.10	3.50	7.04	6.35	0

**Table 3:** The concentration of sugars (µM) in *Neottia ovata* nectar. Data represent the mean ($\bar{x}$) ± standard error (SE), lower quartile (Q1), median(Q2), upper quartile (Q3), interquartile range (IQR). The same letters indicate statistically nonsignificant differences according to the pairwise Wilcoxon Rank Sum test with Benjamini-Hochberg adjustment (*p* ≥ 0.05).

		Population							
Sugar	Statistic	OPA (n = 12)	LUB (n = 13)	POG (n = 22)	ZAB1 (n = 12)	ZAB2 (n = 14)	SKA (n = 17)	TUR (n = 18)	WIS (n = 4)
Glucose	$\bar{x}$ ± SE	64.95 ± 6.80	85.23 ± 9.05	160.85 ± 7.80	59.48 ± 3.78	92.99 ± 7.82	57.86 ± 3.85	73.94 ± 2.40	80.02 ± 3.61
	Q_1_	48.16	65.92	148.83	50.29	73.02	55.10	67.42	75.75
	Q_2_ (IQR)	69.90 (30.10) ^ab^	75.31 (16.17) ^ab^	168.67 (44.14) ^c^	56.15 (18.03) ^a^	93.96 (33.46) ^c^	64.29 (10.31) ^d^	73.77 (14.64) ^ab^	80.13 (8.66) ^bc^
	Q_3_	78.25	82.09	192.96	68.32	106.48	65.41	82.06	84.40
Fructose	$\bar{x}$ ± SE	54.10 ± 5.21	78.77 ± 7.21	99.12 ± 5.84	61.69 ± 4.25	71.64 ± 4.96	61.86 ± 2.55	70.77 ± 2.58	63.52 ± 1.18
	Q_1_	43.57	64.21	85.04	54.79	66.30	55.36	63.50	61.93
	Q_2_ (IQR)	59.90 (22.77) ^ab^	68.75 (11.97) ^a^	100.31 (28.64) ^ab^	57.18 (18.83) ^ab^	70.16 (12.33) ^ab^	65.93 (13.40) ^c^	71.77 (10.85) ^a^	63.30 (2.96) ^b^
	Q_3_	66.34	76.18	113.68	73.62	78.63	68.76	74.35	64.89
Sucrose	$\bar{x}$ ± SE	11.84 ± 2.03	18.88 ± 2.46	19.51 ± 2.19	19.28 ± 2.98	15.96 ± 2.46	10.99 ± 2.08	23.17 ± 2.62	21.87 ± 2.00
	Q_1_	9.26	13.84	11.76	14.53	10.53	6.54	12.69	20.98
	Q_2_ (IQR)	11.08 (4.37) ^ab^	16.27 (4.82) ^a^	18.89 (16.98) ^b^	17.67 (5.99) ^ab^	15.48 (12.18) ^ab^	6.68 (6.40) ^ab^	24.19 (20.70) ^ab^	22.61 (2.51) ^ab^
	Q_3_	13.63	18.66	28.75	20.52	22.70	12.94	33.39	23.49

**Table 4:** The amount of sugars in *Neottia ovata* nectar. The same letters indicate statistically nonsignificant differences according to Tukey’s post-hoc test.

	OPA	LUB	POG	ZAB1	ZAB2	SKA	TUR	WIS
Mean ± SD of total sugars (mg/mL)	25.50 ± 8.79 ^b^	36.01 ± 12.80 ^b^	53.51 ± 12.90 ^a^	28.43 ± 7.82 ^b^	35.12 ± 10.08 ^b^	25.33 ± 6.79 ^b^	34.00 ± 6.63 ^b^	33.34 ± 2.77 ^b^
Glucose content in nectar (*w*/*v*) (%)	1.17	1.54	2.89	1.07	1.68	1.04	1.33	1.44
Fructose content in nectar (*w*/*v*) (%)	0.97	1.42	1.78	1.11	1.29	1.11	1.27	1.14
Sucrose content in nectar (*w*/*v*) (%)	0.41	0.65	0.67	0.66	0.55	0.38	0.79	0.75
Sugar content in nectar (*w*/*v*) (%)	2.55	3.60	5.35	2.84	3.51	2.53	3.40	3.33
Fructose:glucose	0.83	0.92	0.62	1.04	0.77	1.07	0.96	0.79
Sucrose/(fructose + glucose)	0.19	0.22	0.14	0.30	0.18	0.17	0.30	0.29

**Table 5:** The concentration of amino acids (µM) in *Neottia ovata* nectar. Data represent the mean ($\bar{x}$) ± standard error (SE), lower quartile (Q1), median (Q2), upper quartile (Q3), interquartile range (IQR). The same letters indicate statistically nonsignificant differences according to the pairwise Wilcoxon Rank Sum test with Benjamini-Hochberg adjustment (*p* ≥ 0.05). The number of classes represents the effect of amino acids on insect chemoreceptors: I—no effect; II—inhibition of chemoreceptors; III—stimulate the salt cell; IV—the ability to stimulate the sugar cell [12].

			Population							
Amino Acid	Class	Statistic	OPA (n = 12)	LUB (n = 13)	POG (n = 22)	ZAB1 (n = 12)	ZAB2 (n = 14)	SKA (n = 17)	TUR (n = 18)	WIS (n = 4)
**Proteogenic amino acids**										
Asp	I	$\bar{x}$ ± SE	114.90 ± 34.78	258.79 ± 29.19	923.49 ± 97.10	154.67 ± 39.38	259.12 ± 65.73	44.14 ± 8.32	405.13 ± 43.23	296.39 ± 34.05
		Q_1_	39.94	176.71	736.74	39.52	92.58	18.26	297.02	275.96
		Q_2_ (IQR)	73.93 (131.69) ^ab^	259.49 (136.37) ^c^	873.31 (352.41) ^d^	127.18 (223.98) ^abc^	167.22 (269.40) ^ac^	26.50 (57.86) ^b^	397.49 (216.17) ^e^	298.75 (43.22) ^ace^
		Q_3_	171.63	313.08	1089.16	263.50	361.98	76.12	513.19	319.18
Glu	I	$\bar{x}$ ± SE	339.81 ± 56.75	755.19 ± 51.04	2395.91 ± 154.04	434.54 ± 57.93	672.67 ± 148.76	216.76 ± 37.74	800.82 ± 110.67	510.73 ± 69.55
		Q_1_	212.65	635.19	1945.63	236.66	329.39	89.03	478.09	397.38
		Q_2_ (IQR)	403.58 (273.74) ^ab^	785.68 (195.29) ^c^	2284.82 (751.22) ^d^	518.04 (371.77) ^a^	443.10 (382.86) ^ac^	175.38 (212.96) ^b^	798.38 (559.64) ^c^	501.67 (217.64) ^ac^
		Q_3_	486.39	830.48	2696.85	608.43	712.25	301.99	1037.73	615.02
Ala	I	$\bar{x}$ ± SE	402.29 ± 79.45	558.92 ± 61.82	3324.72 ± 348.13	591.94 ± 149.41	573.48 ± 88.64	391.30 ± 95.09	715.04 ± 71.29	481.91 ± 80.45
		Q_1_	247.36	439.60	1978.72	218.60	408.74	123.56	439.03	403.61
		Q_2_ (IQR)	361.57 (241.01) ^a^	516.32 (300.81) ^ab^	3111.70 (2612.50) ^c^	385.60 (766.26) ^ab^	510.81 (207.66) ^ab^	177.95 (459.66) ^a^	716.84 (460.90) ^b^	518.95 (193.64) ^ab^
		Q_3_	488.38	740.41	4591.21	984.85	616.40	583.23	899.93	597.25
Cys	I	$\bar{x}$ ± SE	170.11 ± 30.27	173.11 ± 22.06	732.41 ± 62.49	194.07 ± 53.75	183.14 ± 33.14	124.34 ± 23.34	250.40 ± 30.48	195.32 ± 60.22
		Q_1_	92.36	124.85	541.84	71.35	80.17	70.33	149.91	118.68
		Q_2_ (IQR)	137.97 (149.36) ^ab^	161.86 (71.71) ^ab^	682.44 (224.80) ^c^	126.02 (193.01) ^ab^	157.57 (155.67) ^ab^	111.26 (65.35) ^a^	254.05 (172.38) ^b^	161.43 (119.38) ^ab^
		Q_3_	241.72	196.56	766.63	264.37	235.84	135.68	322.29	238.06
Gly	I	$\bar{x}$ ± SE	139.61 ± 28.14	121.60 ± 15.01	506.45 ± 46.47	126.32 ± 27.03	192.83 ± 43.85	95.29 ± 19.02	163.19 ± 14.15	115.21 ± 23.62
		Q_1_	98.38	87.58	328.59	63.14	108.77	37.49	114.31	80.47
		Q_2_ (IQR)	109.77 (85.36) ^ab^	113.70 (45.14) ^ab^	513.24 (294.99) ^c^	98.99 (114.81) ^ab^	130.81 (97.77) ^a^	74.43 (115.47) ^b^	160.19 (90.30) ^a^	114.07 (68.34) ^ab^
		Q_3_	183.74	132.73	623.58	177.95	206.55	152.96	204.61	148.81
Ser	I	$\bar{x}$ ± SE	53.79 ± 15.35	206.09 ± 17.32	745.52 ± 94.33	153.20 ± 51.36	212.98 ± 48.44	82.30 ± 18.78	322.11 ± 35.99	177.59 ± 26.57
		Q_1_	19.16	186.77	448.70	25.91	132.32	25.94	197.66	135.41
		Q_2_ (IQR)	29.34 (52.48) ^a^	212.33 (50.33) ^bc^	715.29 (568.49) ^d^	76.13 (228.27) ^abe^	166.96 (101.26) ^b^	31.23 (130.75) ^ae^	316.74 (230.35) ^c^	168.08 (74.85) ^bce^
		Q_3_	71.64	237.10	1017.19	254.18	233.58	156.69	428.01	210.26
Thr	I	$\bar{x}$ ± SE	131.63 ± 26.07	128.20 ± 12.19	351.60 ± 50.49	149.27 ± 35.45	140.66 ± 25.20	101.54 ± 18.39	168.98 ± 14.22	118.27 ± 12.90
		Q_1_	77.30	106.53	108.57	51.90	85.46	44.89	128.16	99.85
		Q_2_ (IQR)	105.86 (88.10) ^ab^	116.73 (33.95) ^ab^	345.52 (403.22) ^a^	111.56 (225.62) ^ab^	117.61 (78.14) ^ab^	75.12 (79.41) ^b^	167.10 (85.02) ^a^	117.13 (35.70) ^ab^
		Q_3_	165.40	140.48	511.79	277.53	163.60	124.31	213.17	135.55
Tyr	I	$\bar{x}$ ± SE	10.24 ± 4.35	30.30 ± 9.86	101.24 ± 18.59	19.73 ± 7.27	18.79 ± 8.64	4.56 ± 3.28	2.48 ± 1.41	4.81 ± 4.81
		Q_1_	0.00	0.00	29.35	0.00	0.00	0.00	0.00	0.00
		Q_2_ (IQR)	0.00 (18.82) ^abc^	15.09 (42.50) ^a^	74.45 (122.45) ^d^	4.74 (37.97) ^ab^	0.00 (32.27) ^abc^	0.00 (0.00) ^bc^	0.00 (0.00) ^c^	0.00 (4.81) ^abc^
		Q_3_	18.82	42.50	151.80	37.97	32.27	0.00	0.00	4.81
Arg	II	$\bar{x}$ ± SE	17.58 ± 4.57	24.62 ± 4.69	78.84 ± 12.67	26.18 ± 5.95	43.45 ± 12.93	15.94 ± 4.28	24.32 ± 4.07	14.61 ± 6.08
		Q_1_	8.18	13.41	22.48	8.48	8.69	0.00	13.17	8.00
		Q_2_ (IQR)	15.44 (12.65) ^a^	29.55 (27.28) ^ab^	85.17 (103.93) ^b^	19.20 (39.33) ^ab^	27.41 (61.44) ^ab^	13.86 (21.80) ^a^	17.65 (23.61) ^a^	14.95 (13.55) ^ab^
		Q_3_	20.83	40.69	126.41	47.81	70.14	21.80	36.78	21.55
Asn	II	$\bar{x}$ ± SE	60.01 ± 22.28	344.62 ± 59.19	1448.64 ± 236.17	212.85 ± 78.93	252.49 ± 111.30	46.00 ± 12.32	280.67 ± 43.12	178.70 ± 36.53
		Q_1_	10.88	200.50	798.45	19.63	17.64	13.43	182.66	133.28
		Q_2_ (IQR)	17.96 (72.58) ^ab^	345.78 (259.59) ^c^	1169.15 (856.17) ^d^	82.46 (282.10) ^abc^	55.56 (253.22) ^abc^	24.67 (39.87) ^a^	229.73 (154.64) ^c^	151.80 (63.94) ^bc^
		Q_3_	83.46	460.09	1654.63	301.73	270.86	53.30	337.30	197.22
Gln	II	$\bar{x}$ ± SE	92.01 ± 37.32	338.85 ± 59.08	1011.30 ± 218.53	384.08 ± 109.89	373.92 ± 132.31	119.67 ± 22.68	441.51 ± 63.80	208.35 ± 67.29
		Q_1_	33.21	223.92	446.99	28.86	126.66	55.79	248.54	133.84
		Q_2_ (IQR)	53.19 (58.73) ^a^	294.41 (220.74) ^b^	711.60 (604.83) ^c^	342.75 (609.76) ^abd^	203.08 (293.26) ^bd^	65.23 (158.28) ^ad^	447.78 (331.45) ^b^	224.13 (164.80) ^abd^
		Q_3_	91.93	444.66	1051.82	638.62	419.92	214.07	579.99	298.64
His	II	$\bar{x}$ ± SE	17.43 ± 6.28	155.67 ± 22.72	397.86 ± 38.95	15.70 ± 4.37	228.64 ± 70.16	48.83 ± 9.41	109.24 ± 15.75	38.14 ± 12.43
		Q_1_	2.83	101.98	245.04	4.79	56.16	21.27	64.36	25.16
		Q_2_ (IQR)	6.14 (26.03) ^a^	149.72 (76.04) ^b^	351.39 (302.51) ^c^	13.76 (15.28) ^a^	130.01 (151.81) ^b^	48.83 (44.11) ^d^	93.45 (88.48) ^b^	41.44 (29.25) ^ad^
		Q_3_	28.86	178.01	547.56	20.07	207.97	65.38	152.84	54.41
Lys	II	$\bar{x}$ ± SE	37.15 ± 13.63	58.05 ± 8.24	389.39 ± 51.81	82.89 ± 27.14	87.32 ± 24.46	61.59 ± 16.32	110.20 ± 15.52	64.39 ± 18.46
		Q_1_	11.21	28.22	215.21	10.43	40.43	17.31	57.70	35.61
		Q_2_ (IQR)	19.28 (26.07) ^a^	61.79 (51.87) ^abc^	335.74 (333.13) ^d^	33.51 (137.00) ^abc^	64.20 (59.81) ^bc^	35.58 (76.61) ^ab^	84.39 (97.23) ^c^	61.18 (54.35) ^abc^
		Q_3_	37.29	80.09	548.34	147.42	100.24	93.92	154.93	89.96
Pro	III	$\bar{x}$ ± SE	72.45 ± 16.97	303.90 ± 37.63	1159.30 ± 99.85	106.13 ± 24.51	383.73 ± 92.20	69.36 ± 12.31	336.08 ± 61.83	80.50 ± 18.19
		Q_1_	22.18	218.12	800.62	35.06	170.61	32.87	200.48	58.47
		Q_2_ (IQR)	75.19 (90.30) ^a^	328.10 (133.69) ^b^	1013.74 (675.10) ^c^	112.77 (113.32) ^a^	296.34 (166.64) ^b^	59.87 (52.86) ^a^	336.27 (203.78) ^b^	67.35 (30.92) ^a^
		Q_3_	112.48	351.81	1475.72	148.38	337.26	85.74	404.26	89.38
Ile	IV	$\bar{x}$ ± SE	101.47 ± 17.98	139.14 ± 14.56	452.06 ± 39.40	161.12 ± 53.32	142.63 ± 29.08	81.21 ± 19.36	239.01 ± 31.25	146.79 ± 17.76
		Q_1_	59.14	109.40	334.50	26.89	76.93	30.90	126.10	123.77
		Q_2_ (IQR)	97.27 (98.19) ^ab^	138.68 (43.90) ^ac^	439.52 (155.53) ^d^	100.01 (174.61) ^abc^	116.72 (99.30) ^ab^	62.65 (73.69) ^b^	207.61 (165.25) ^c^	149.76 (49.01) ^ac^
		Q_3_	157.33	153.31	490.03	201.50	176.22	104.59	291.35	172.78
Leu	IV	$\bar{x}$ ± SE	220.90 ± 34.29	255.87 ± 23.30	934.70 ± 60.70	258.17 ± 57.32	260.36 ± 56.28	132.27 ± 24.78	385.27 ± 38.22	256.47 ± 33.52
		Q_1_	138.39	217.03	758.48	35.19	120.63	44.87	266.85	233.41
		Q_2_ (IQR)	211.82 (193.35) ^ab^	250.99 (67.78) ^a^	888.18 (279.22) ^c^	277.10 (402.64) ^abd^	221.51 (151.65) ^abd^	109.11 (163.00) ^b^	420.63 (229.31) ^d^	260.92 (50.56) ^abd^
		Q_3_	331.75	284.80	1037.70	437.83	272.28	207.87	496.16	283.98
Met	IV	$\bar{x}$ ± SE	22.33 ± 3.92	15.81 ± 2.13	30.42 ± 2.86	31.41 ± 2.96	30.74 ± 6.98	7.62 ± 3.01	24.30 ± 3.78	7.58 ± 5.50
		Q_1_	11.50	10.44	20.30	25.72	2.99	0.00	13.84	0.00
		Q_2_ (IQR)	28.28 (20.85) ^ab^	17.89 (11.74) ^ac^	27.73 (18.47) ^b^	28.41 (7.33) ^b^	32.70 (42.63) ^ab^	0.00 (12.32) ^c^	24.44 (18.37) ^ab^	3.49 (11.07) ^ac^
		Q_3_	32.35	22.17	38.77	33.06	45.63	12.32	32.21	11.07
Phe	IV	$\bar{x}$ ± SE	110.48 ± 17.14	132.80 ± 17.35	461.23 ± 26.52	156.89 ± 41.20	118.70 ± 20.79	77.69 ± 13.22	164.46 ± 18.43	97.28 ± 13.26
		Q_1_	80.67	114.91	386.95	32.06	74.64	37.48	93.62	77.60
		Q_2_ (IQR)	106.12 (63.57) ^ab^	129.69 (32.66) ^a^	448.66 (149.68) ^c^	141.74 (197.18) ^ab^	101.08 (59.09) ^ab^	70.90 (53.69) ^b^	159.20 (108.91) ^a^	97.12 (39.19) ^ab^
		Q_3_	144.24	147.57	536.63	229.24	133.73	91.17	202.54	116.79
Trp	IV	$\bar{x}$ ± SE	62.87 ± 10.81	103.53 ± 10.00	181.22 ± 19.70	65.93 ± 13.74	72.15 ± 14.17	55.42 ± 9.41	102.43 ± 15.29	92.35 ± 28.59
		Q_1_	41.92	63.64	127.52	29.45	31.33	31.26	49.03	62.00
		Q_2_ (IQR)	56.85 (41.94) ^ab^	119.90 (67.55) ^c^	151.57 (68.77) ^d^	62.45 (72.09) ^abc^	55.52 (61.12) ^abc^	49.10 (35.97) ^a^	86.06 (82.37) ^bc^	69.82 (38.17) ^abcd^
		Q_3_	83.85	131.19	196.29	101.54	92.45	67.23	131.40	100.17
Val	IV	$\bar{x}$ ± SE	49.48 ± 22.50	80.96 ± 27.94	116.49 ± 15.57	95.92 ± 52.29	25.57 ± 17.67	197.81 ± 43.41	108.59 ± 33.25	248.59 ± 66.92
		Q_1_	8.80	22.65	64.26	0.00	0.00	84.84	22.18	229.28
		Q_2_ (IQR)	16.74 (40.02) ^ab^	26.08 (83.53) ^a^	94.40 (87.82) ^cd^	13.11 (86.12) ^abe^	0.66 (12.39) ^b^	183.90 (130.31) ^c^	50.82 (78.80) ^ade^	289.59 (79.62) ^cde^
		Q_3_	48.82	106.18	152.08	86.12	12.39	215.15	100.98	308.90
**Non-proteogenic amino acids**										
Orn		$\bar{x}$ ± SE	153.64 ± 88.35	126.73 ± 10.87	134.27 ± 14.46	96.86 ± 27.01	55.19 ± 9.62	37.66 ± 7.55	134.56 ± 11.75	84.90 ± 11.86
		Q_1_	39.32	99.23	76.40	7.81	32.90	11.24	89.02	72.34
		Q_2_ (IQR)	72.35 (71.66) ^ab^	134.77 (45.44) ^a^	103.38 (109.81) ^a^	78.80 (171.73) ^ab^	53.61 (30.28) ^b^	23.75 (53.99) ^b^	142.82 (87.98) ^a^	88.30 (28.52) ^ab^
		Q_3_	110.97	144.66	186.22	179.54	63.18	65.24	177.00	100.86
Cit		$\bar{x}$ ± SE	10.81 ± 2.35	15.84 ± 2.27	58.45 ± 13.10	7.64 ± 2.72	25.72 ± 8.72	4.14 ± 2.25	10.21 ± 2.77	7.53 ± 4.43
		Q_1_	4.47	10.84	28.44	0.00	0.00	0.00	0.00	0.00
		Q_2_ (IQR)	11.67 (10.63) ^a^	17.85 (9.46) ^a^	36.65 (27.01) ^b^	3.81 (13.89) ^ac^	11.12 (35.77) ^a^	0.00 (0.00) ^c^	4.64 (18.33) ^ac^	6.49 (14.02) ^ac^
		Q_3_	15.10	20.29	55.46	13.89	35.77	0.00	18.33	14.02
Tau		$\bar{x}$ ± SE	59.85 ± 18.63	63.83 ± 17.30	121.95 ± 17.04	55.39 ± 15.94	135.37 ± 32.55	62.97 ± 10.85	82.06 ± 14.64	64.33 ± 44.18
		Q_1_	2.93	17.10	67.07	0.00	28.06	37.00	47.28	15.29
		Q_2_ (IQR)	44.09 (93.96) ^a^	39.37 (59.25) ^a^	102.23 (108.07) ^a^	55.81 (101.43) ^a^	111.77 (204.77) ^a^	61.84 (51.65) ^a^	62.94 (57.95) ^a^	31.54 (65.29) ^a^
		Q_3_	96.89	76.35	175.13	101.43	232.82	88.64	105.23	80.58
AABA		$\bar{x}$ ± SE	16.04 ± 5.27	22.01 ± 5.50	3.64 ± 2.93	26.22 ± 11.74	29.80 ± 8.95	13.91 ± 3.45	50.51 ± 7.12	57.26 ± 29.22
		Q_1_	0.00	11.38	0.00	0.00	1.65	0.00	25.64	21.07
		Q_2_ (IQR)	9.24 (29.53) ^a^	16.41 (19.36) ^a^	0.00 (0.00) ^b^	5.68 (32.69) ^a^	27.32 (43.50) ^ac^	13.18 (15.72) ^a^	43.22 (42.48) ^c^	42.17 (57.30) ^ac^
		Q_3_	29.53	30.74	0.00	32.69	45.15	15.72	68.12	78.37
BABA		$\bar{x}$ ± SE	19.90 ± 7.25	6.84 ± 2.80	38.33 ± 7.54	12.48 ± 6.94	14.99 ± 9.62	18.28 ± 4.29	8.94 ± 3.23	18.46 ± 12.21
		Q_1_	0.00	0.00	12.44	0.00	0.00	0.00	0.00	0.00
		Q_2_ (IQR)	10.17 (30.44) ^ab^	0.00 (10.99) ^a^	31.21 (35.59) ^b^	0.00 (11.02) ^ab^	0.00 (5.96) ^a^	20.29 (29.93) ^ab^	0.00 (19.74) ^a^	11.17 (29.63) ^ab^
		Q_3_	30.44	10.99	48.03	11.02	5.96	29.93	19.74	29.63
GABA		$\bar{x}$ ± SE	94.97 ± 30.51	41.58 ± 10.22	500.42 ± 77.81	138.96 ± 44.84	161.11 ± 35.08	116.97 ± 24.03	62.50 ± 19.01	118.27 ± 73.87
		Q_1_	21.78	14.23	194.27	0.00	59.13	22.42	0.00	48.23
		Q_2_ (IQR)	60.89 (89.58) ^ab^	41.48 (54.03) ^a^	455.26 (427.76) ^c^	87.63 (235.14) ^ab^	129.89 (222.39) ^b^	102.89 (163.50) ^ab^	0.00 (144.08) ^ab^	69.39 (91.20) ^ab^
		Q_3_	111.36	68.26	622.03	235.14	281.52	185.92	144.08	139.43
β-Ala		$\bar{x}$ ± SE	11.42 ± 8.46	21.52 ± 9.13	0.00 ± 0.00	7.87 ± 4.37	0.00 ± 0.00	28.31 ± 19.34	7.58 ± 6.42	0.00 ± 0.00
		Q_1_	0.00	0.00	0.00	0.00	0.00	0.00	0.00	0.00
		Q_2_ (IQR)	0.00 (2.02)	0.00 (30.49)	0.00 (0.00)	0.00 (6.28)	0.00 (0.00)	0.00 (20.83)	0.00 (0.00)	0.00 (0.00)
		Q_3_	2.02	30.49	0.00	6.28	0.00	20.83	0.00	0.00
Nva		$\bar{x}$ ± SE	6.95 ± 3.33	4.05 ± 1.75	62.30 ± 8.17	4.06 ± 2.23	1.71 ± 0.92	11.44 ± 2.52	0.00 ± 0.00	0.00 ± 0.00
		Q_1_	0.00	0.00	42.07	0.00	0.00	0.00	0.00	0.00
		Q_2_ (IQR)	0.76 (8.41)	0.00 (6.52)	55.42 (31.73)	0.00 (5.27)	0.00 (0.00)	15.18 (19.73)	0.00 (0.00)	0.00 (0.00)
		Q_3_	8.41	6.52	73.81	5.27	0.00	19.73	0.00	0.00

**Table 6:** Sugar and amino acid ratio in *Neottia ovata* populations.

Population	Total Sugars [mg/mL]	Total AAs [mg/mL]	Total Sugars/Total AAs
OPA	306.01	3.71	82.41
LUB	468.12	6.93	67.54
POG	1177.32	42.37	27.79
ZAB1	341.13	5.42	62.89
ZAB2	491.74	7.87	62.48
SKA	430.61	4.55	94.56
TUR	612.02	11.76	52.02
WIS	133.38	1.74	76.68

**Table 7:** Spatial and temporal variation of FRS and PR in *Neottia ovata* populations. Data show the mean ± standard deviation.

Population	Year	FRS (%)	PR (%)	PR:FRS
OPA	2019	72.41 ± 20.12	98.44 ± 4.42	1.19 ± 0.22
	2020	63.00 ± 29.56	92.75 ± 11.98	1.44 ± 0.52
LUB	2019	93.74 ± 9.58	90.10 ± 16.59	0.97 ± 0.19
	2020	73.65 ± 18.52	92.11 ± 6.69	1.36 ± 45
POG	2019	nd	nd	nd
	2020	86.76 ± 14.83	86.97 ± 33.98	1.04 ± 0.49
ZAB1	2019	69.59 ± 25.92	97.09 ± 6.50	1.40 ± 0.63
	2020	75.24 ± 21.10	95.00 ± 6.21	1.42 ± 0.68
ZAB2	2019	86.63 ± 14.69	93.45 ± 7.76	1.12 ± 0.23
	2020	44.77 ± 24.78	94.80 ± 7.90	4.65 ± 6.83
TUR	2019	40.73 ± 16.45	nd	nd
	2020	41.02 ± 27.86	81.61 ± 16.33	5.55 ± 8.36
WIS	2019	nd	nd	nd
	2020	38.69 ± 34.35	88.35 ± 18.58	4.81 ± 4.67
SKA	2019	44.35 ± 14.16	nd	nd
	2020	14.85 ± 8.34	85.48 ± 35.56	9.11 ± 5.13

nd—no data.

**Table 8:** Habitat characteristics for *Neottia ovata* populations in northeast Poland.

Region	Population	Habitat Characteristics
Biebrza National Park (BNP)	OPA	Mineral elevation with domination of *Betula pendula* in tree layer, and in undergrowth layer species characteristic for broadleaved forests
	LUB	At the border of mineral island covered by broadleaved forests
	POG	The border of alder forest and peat bogs, partly in open area with domination of grasses and sedges, and partly under shrubs and trees canopy
	ZAB1	Mineral island dominated by open space, covered mainly by grasses and sedges, with patches of shrubs and trees at the border
	ZAB2	Mineral, island dominated by open space, covered mainly by grasses and sedges, with patches of shrubs and single trees at the border
Suwalki Landscape Park (NLP)	TUR	Under canopy of fragment of alder forest with loose undergrowth layer
	WIS	Shallow lowland springs rich in mosses and *Equisetum telmateia*
Knyszynska Forest (KF)	SKA	The small patch of birch forest with domination of sedges, at the foot of the railway embankment

## Data Availability

Data is contained within the current article and supplementary material.

## Supplementary Materials

The following are available online at https://www.mdpi.com/1422-0067/22/4/2214/s1, Figure S1: Boxplots of amino acids concentration for *Neottia ovata* populations. Colored dots are individual samples. The crossed square shows the mean. The lower and upper hinges correspond to the lower (Q1) and upper (Q3) quartiles. Thus box length shows the interquartile range (IQR). The thicker line inside boxes corresponds to the median. The lower whisker extends from the hinge to the smallest value at most Q1 − 1.5 × IQR of the hinge. The upper whisker extends from the hinge to the largest value no further than Q3 + 1.5 × IQR. Data beyond the end of the whiskers, indicated with an asterisk symbol, are outliers, Figure S2: Scree plot showing the proportion of explained variance by the principal components, Figure S3: Cos^2^ for the amino acids selected as active variables in the principal component analysis model, representing the quality of representation for variables on the factor map (Dim1-3), Figure S4: Uniform manifold approximation and projection of all amino acids in *Neottia ovata* populations, except for β-Ala. Individuals (populations) are color-coded and labeled with a number corresponding to Id used in Table S3, Table S1: Amino acids transformation using Tukey’s Ladder of Power, Table S2: Kaiser-Meyer-Olkin test results sorted in descending order by the measure of sampling adequacy (MSA) (overall MSA = 0.92), Table S3: Amino acids dataset used in PCA and UMAP analyses.
