# Supplementary material for: How Are the Flower Structure and Nectar Composition of the Generalistic Orchid Neottia ovata Adapted to a Wide Range of Pollinators?
Source: Int J Mol Sci. 2021 Feb 23;22(4):2214. doi: 10.3390/ijms22042214 (PMC7926835; doi:10.3390/ijms22042214)
Supplement: Supplementary file 1 [file ijms-22-02214-s001.zip › Supplementary materials - Tables S1-2 and Figures S1-4.docx]

**Table S1.** Amino acids transformation using Tukey’s Ladder of Power:

$y=\left\{ \begin{matrix} x^{\lambda} \\ log(x) \\ -(x^{\lambda}) \end{matrix}\begin{matrix} , if \lambda>0 \\ , if \lambda=0 \\ , if \lambda<0 \end{matrix} \right\}$, where y – new value, x – original value.

| **Amino acid** | **Number of iterations** | **λ** | **W statistic** | ***p*-value** |
| --- | --- | --- | --- | --- |
| Asp | 2078 | 0.385 | 0.9857 | 0.2798 |
| Glu | 2042 | 0.205 | 0.9822 | 0.1411 |
| Asn | 2048 | 0.235 | 0.9867 | 0.3380 |
| Ser | 2038 | 0.185 | 0.9811 | 0.1139 |
| Gln | 2055 | 0.270 | 0.9795 | 0.08244 |
| His | 2063 | 0.310 | 0.9863 | 0.3146 |
| Gly | 2065 | 0.320 | 0.9686 | 0.009655 |
| Thr | 2079 | 0.390 | 0.9756 | 0.03763 |
| Cit | 2083 | 0.410 | 0.8806 | 5.183e-08 |
| Arg | 2090 | 0.445 | 0.9517 | 0.0004818 |
| Ala | 1989 | -0.060 | 0.9839 | 0.1981 |
| Tau | 2111 | 0.550 | 0.9699 | 0.01231 |
| GABA | 2081 | 0.400 | 0.9497 | 0.0003495 |
| BABA | 2103 | 0.510 | 0.8189 | 2.054e-10 |
| Tyr | 2074 | 0.365 | 0.8103 | 1.053e-10 |
| AABA | 2103 | 0.510 | 0.8793 | 4.537e-08 |
| Cys | 2020 | 0.095 | 0.9920 | 0.7597 |
| Val | 2075 | 0.370 | 0.9718 | 0.01793 |
| Met | 2167 | 0.830 | 0.9497 | 0.0003468 |
| Nva | 2075 | 0.370 | 0.7778 | 9.977e-12 |
| Trp | 2092 | 0.455 | 0.9845 | 0.2221 |
| Phe | 2078 | 0.385 | 0.9725 | 0.02038 |
| Ile | 2070 | 0.345 | 0.9882 | 0.4378 |
| Orn | 2078 | 0.385 | 0.9294 | 1.682e-05 |
| Leu | 2062 | 0.305 | 0.9868 | 0.3403 |
| Lys | 2059 | 0.290 | 0.9722 | 0.01927 |
| Pro | 2061 | 0.300 | 0.9819 | 0.134 |

**Table S2.** Kaiser-Meyer-Olkin test results sorted in descending order by the measure of sampling adequacy (MSA) (overall MSA = 0.92).

| **Amino acid** | **MSA** | **Variable type** |
| --- | --- | --- |
| Asp | 0.95 | Active |
| Glu | 0.95 | Active |
| Asn | 0.95 | Active |
| Ser | 0.95 | Active |
| Cys | 0.95 | Active |
| Trp | 0.95 | Active |
| Ile | 0.95 | Active |
| Orn | 0.95 | Active |
| Leu | 0.95 | Active |
| Lys | 0.95 | Active |
| Ala | 0.94 | Active |
| Phe | 0.94 | Active |
| Gly | 0.93 | Active |
| Cit | 0.93 | Active |
| Tyr | 0.93 | Active |
| Pro | 0.93 | Active |
| His | 0.90 | Active |
| Gln | 0.88 | Active |
| Thr | 0.88 | Active |
| GABA | 0.88 | Active |
| Nva | 0.86 | Supplementary |
| Arg | 0.85 | Active |
| Met | 0.74 | Supplementary |
| BABA | 0.69 | Supplementary |
| Val | 0.62 | Supplementary |
| AABA | 0.54 | Supplementary |
| Tau | 0.53 | Supplementary |

**Table S3.** Amino acids dataset used in PCA and UMAP analyses.

*Dataset is in Table3S.xlsx file.*

**Figure S1.** Boxplots of amino acids concentration for *Neottia ovata* populations. Colored dots are individual samples. The crossed square shows the mean. The lower and upper hinges correspond to the lower (Q_1_) and upper (Q_3_) quartiles. Thus box length shows the interquartile range (IQR). The thicker line inside boxes corresponds to the median. The lower whisker extends from the hinge to the smallest value at most Q_1_ - 1.5 × IQR of the hinge. The upper whisker extends from the hinge to the largest value no further than Q_3_ + 1.5 × IQR. Data beyond the end of the whiskers, indicated with an asterisk symbol, are outliers.


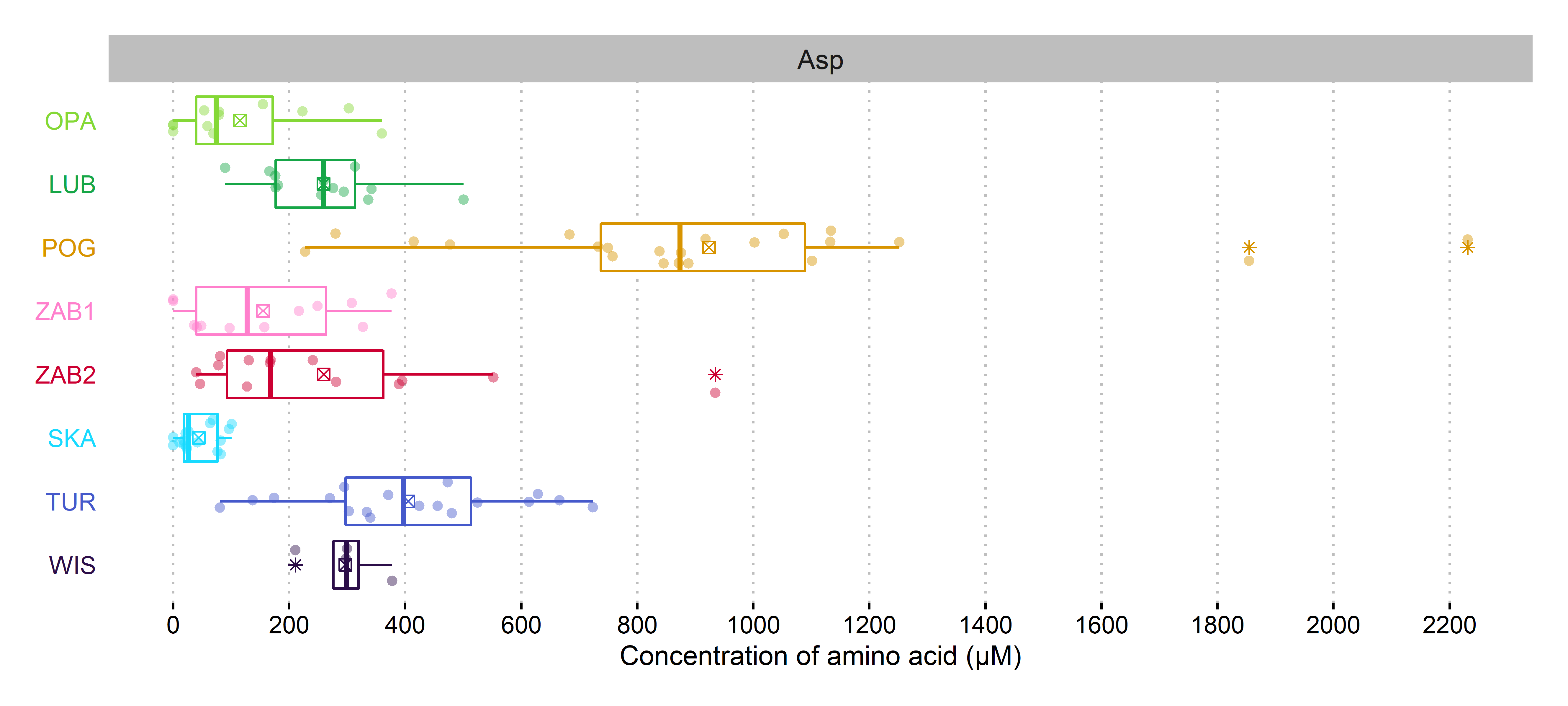

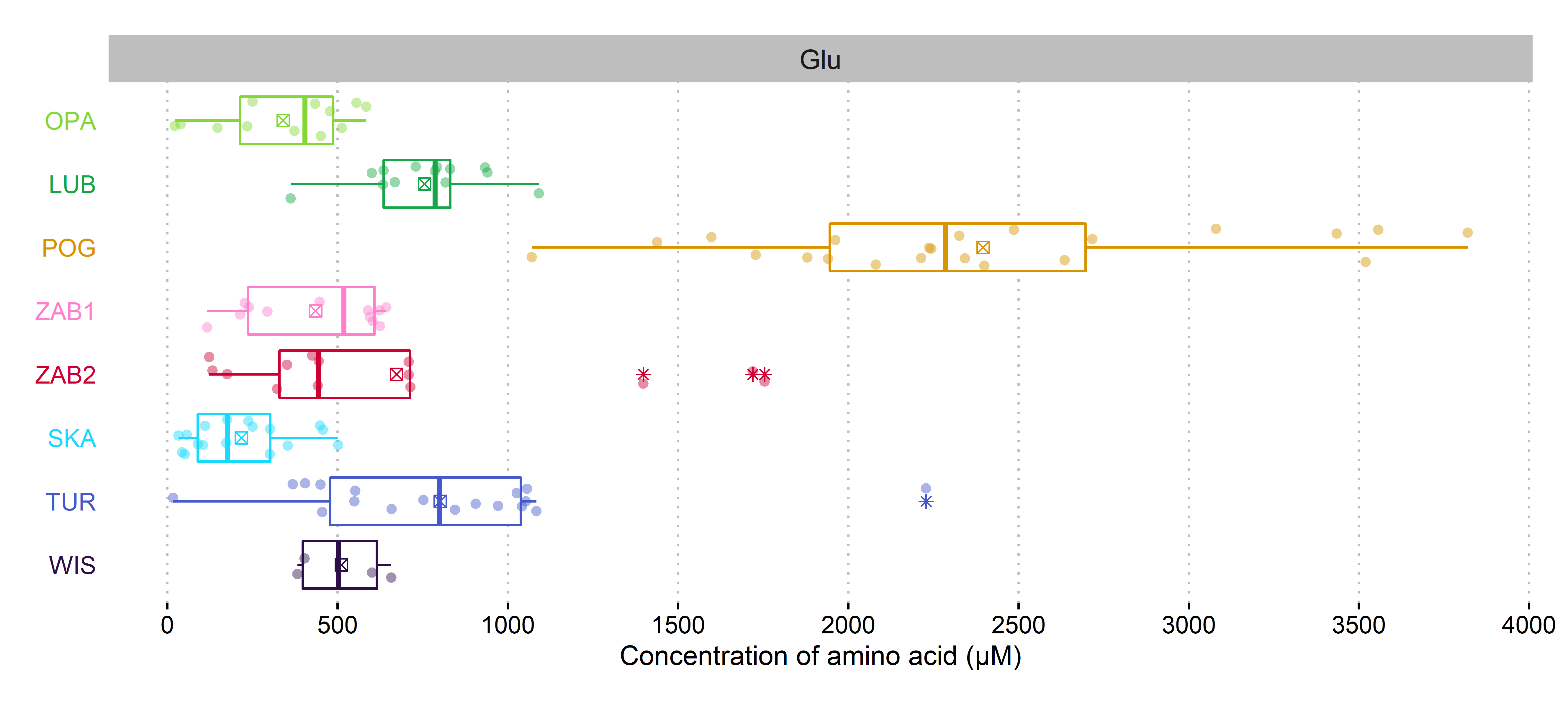

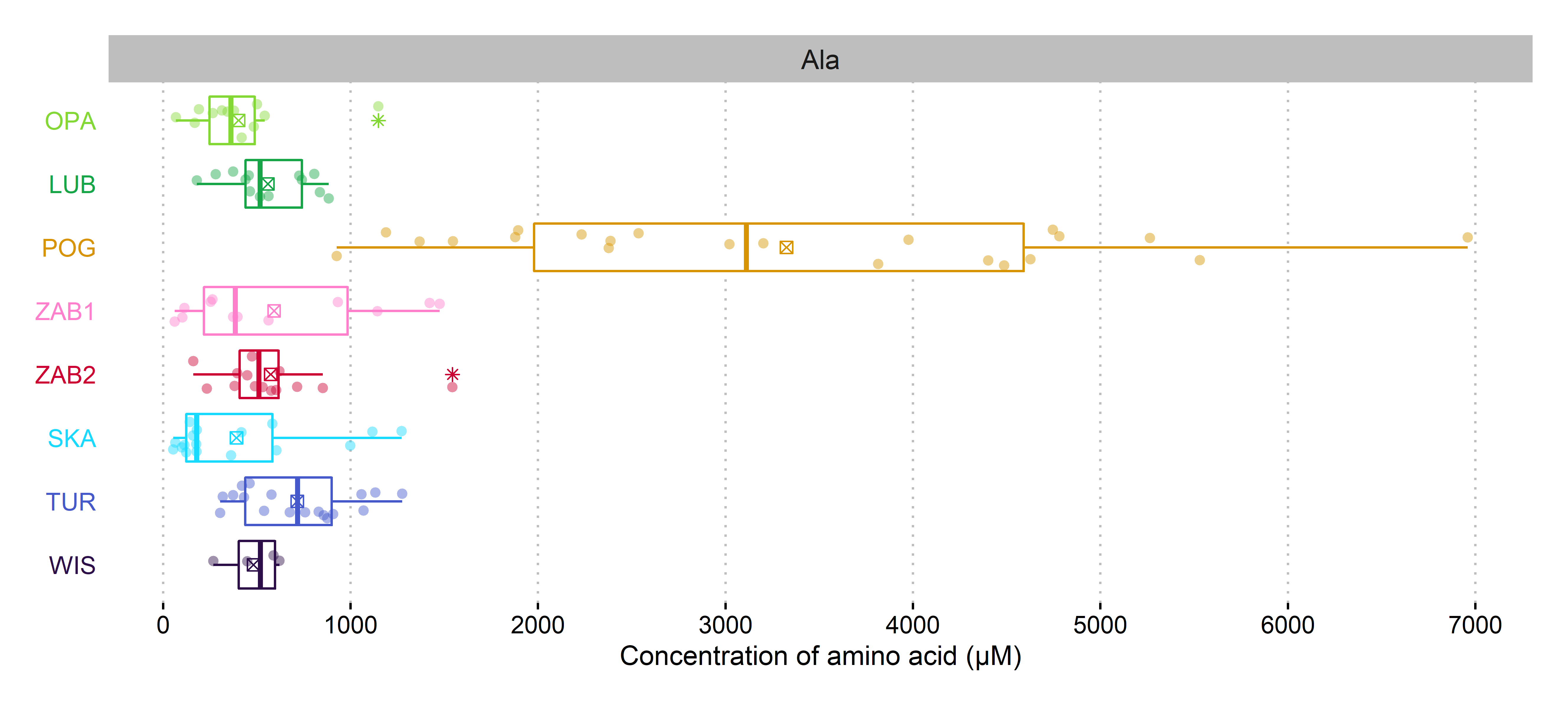

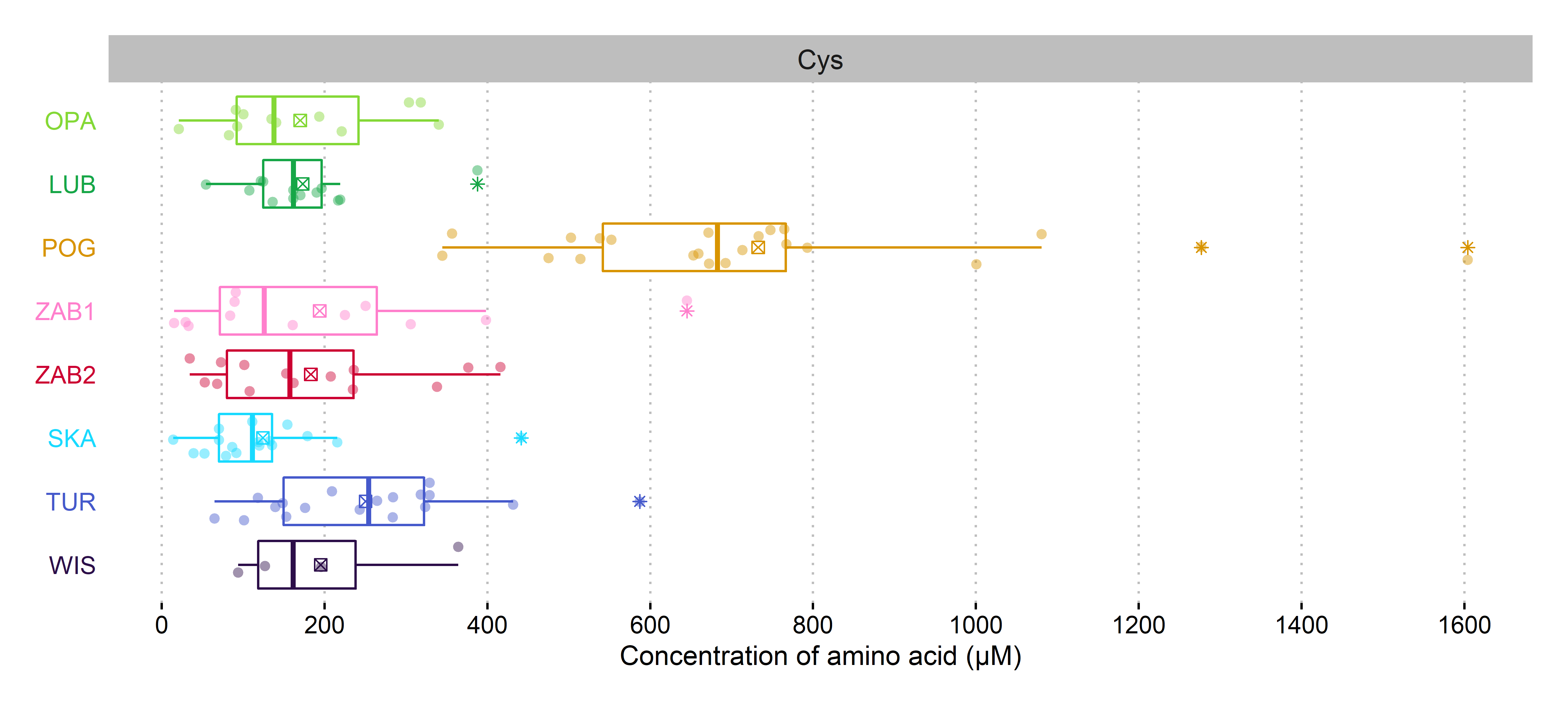

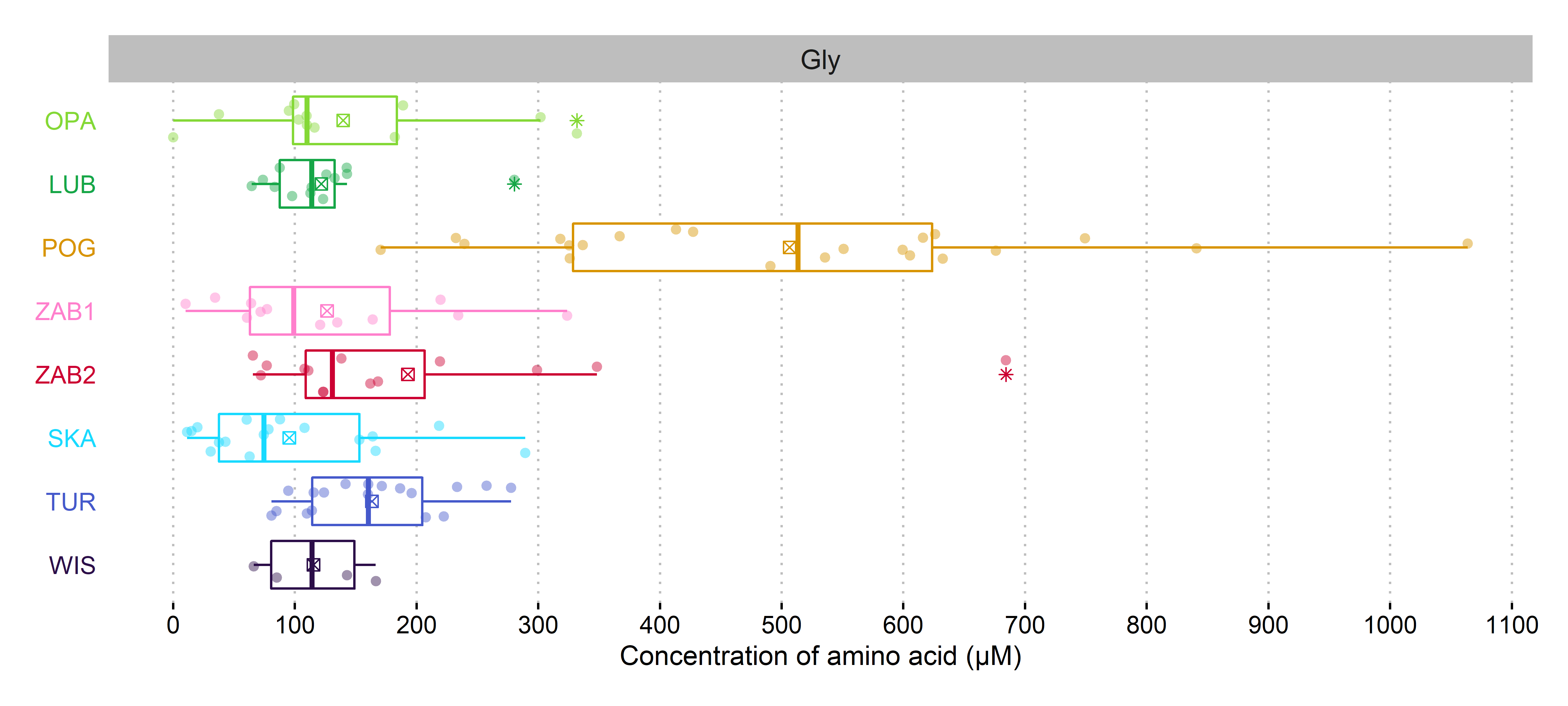

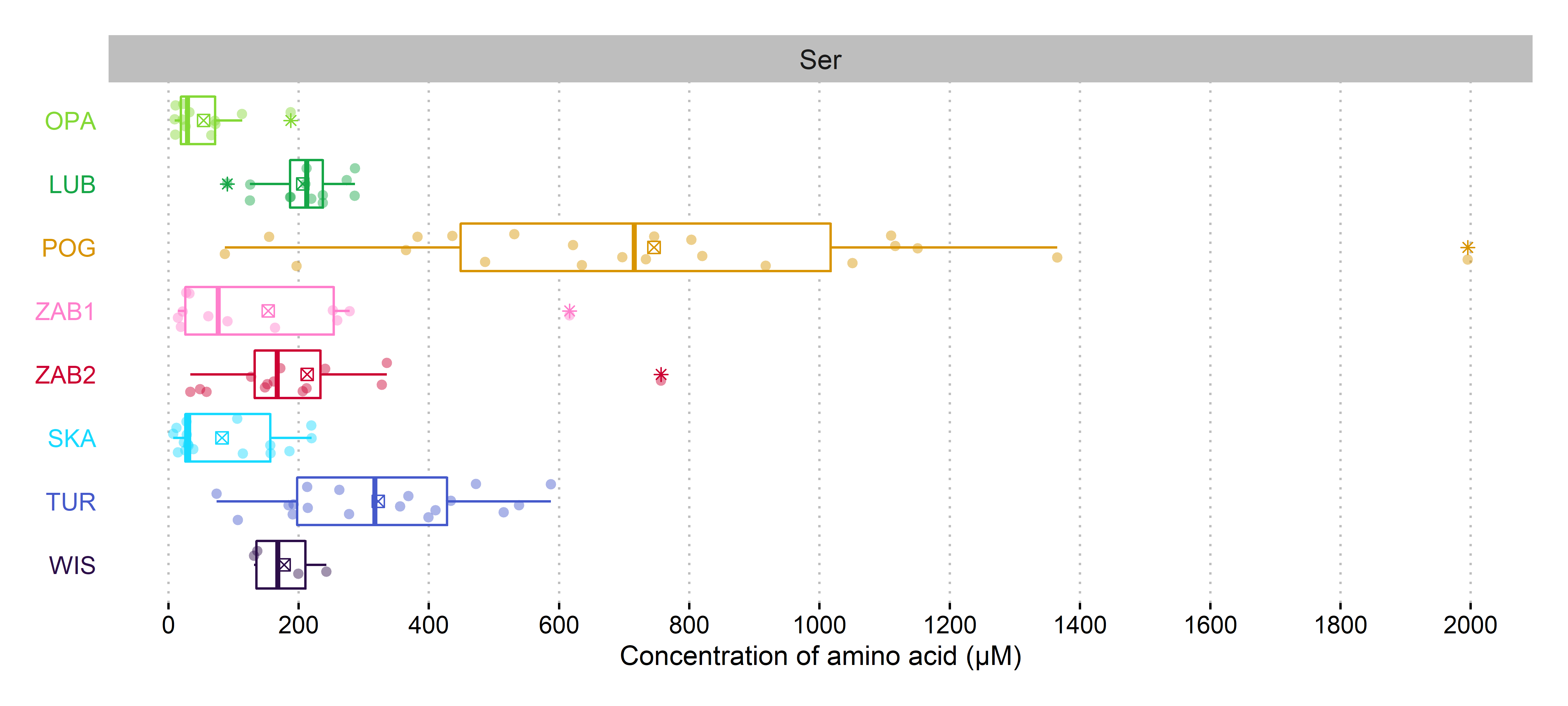

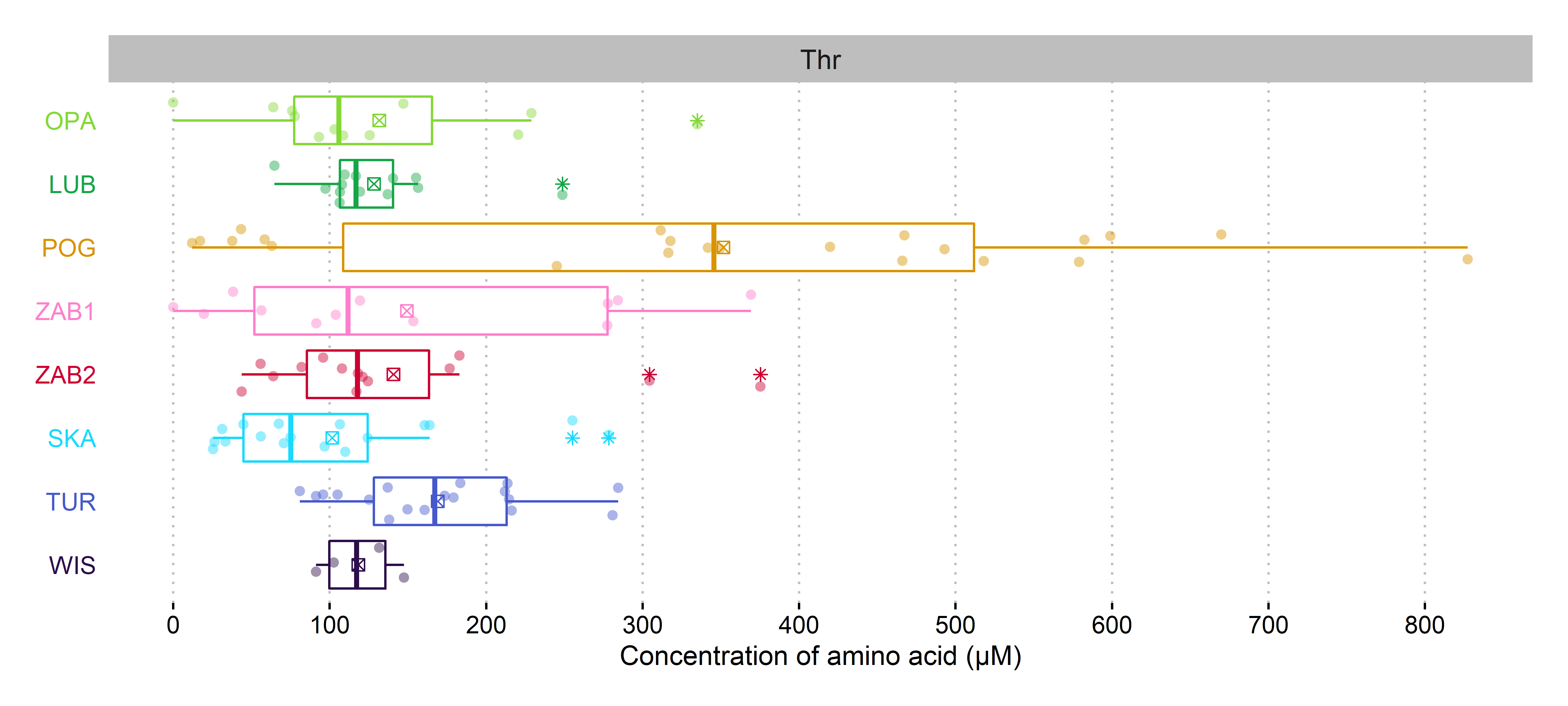

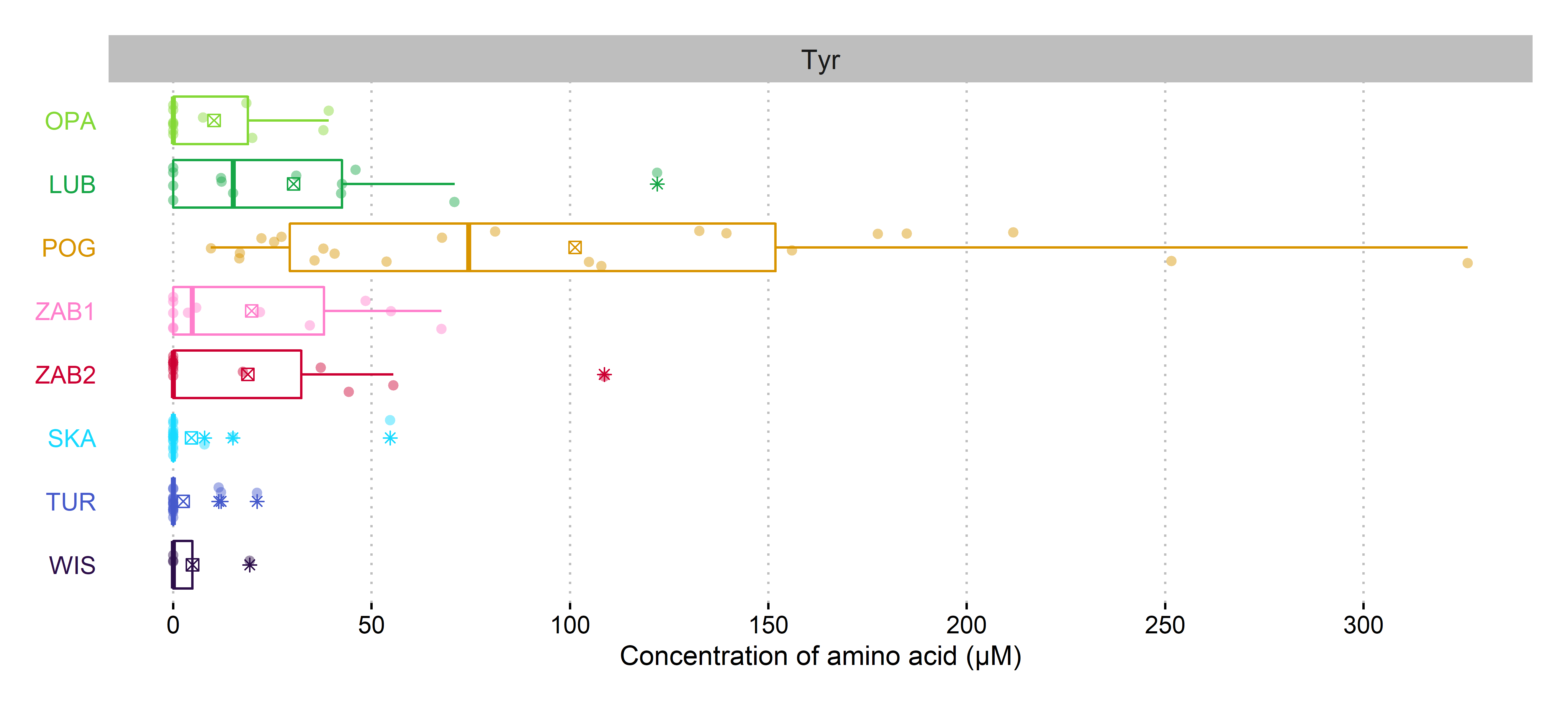

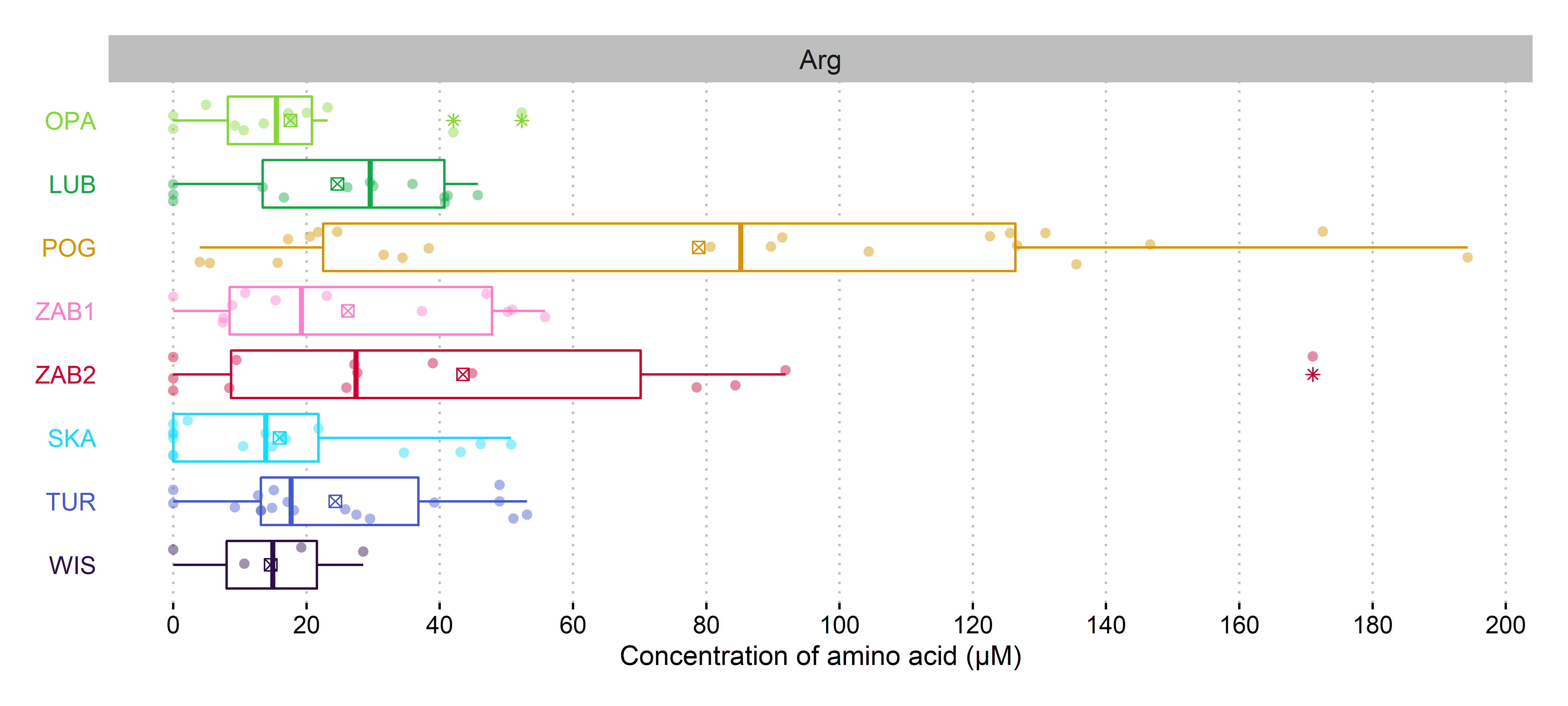

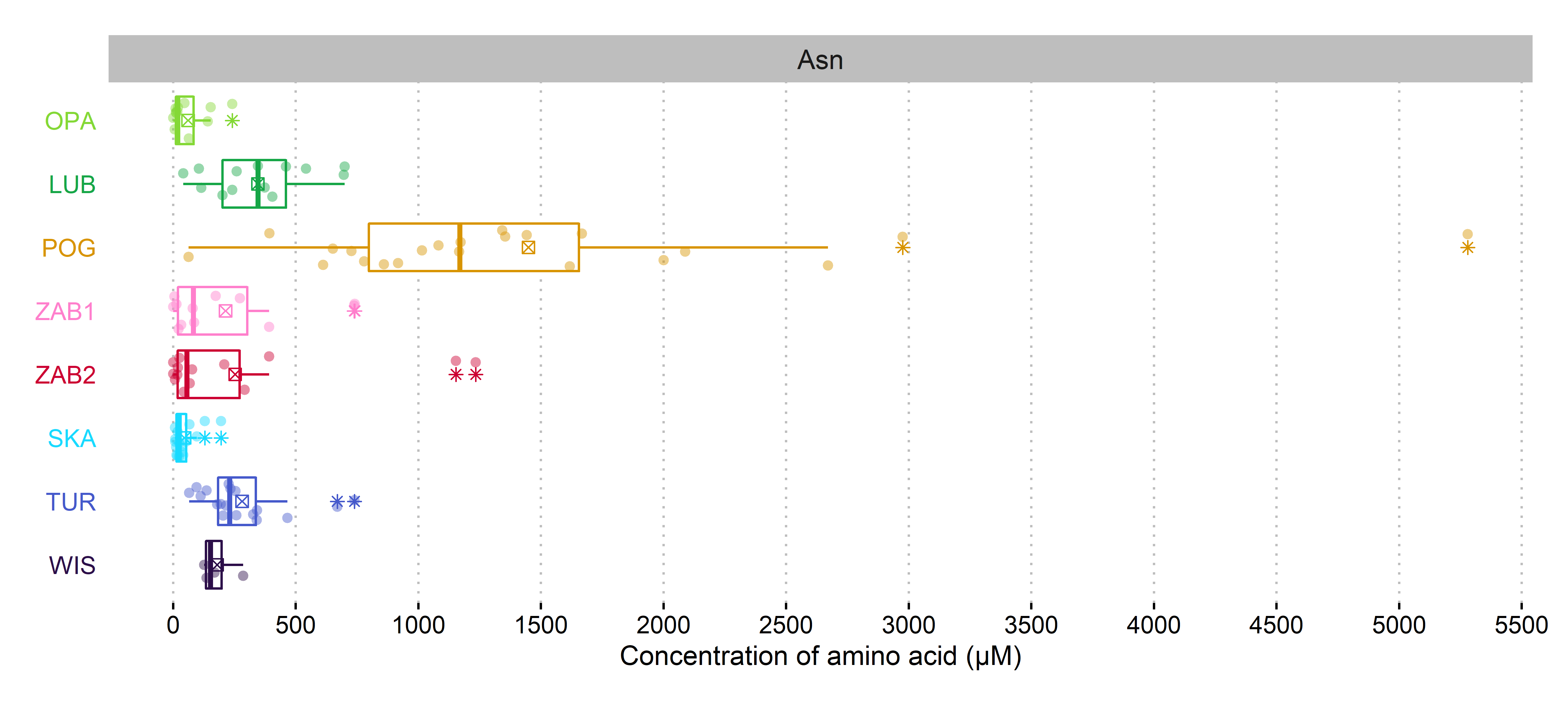

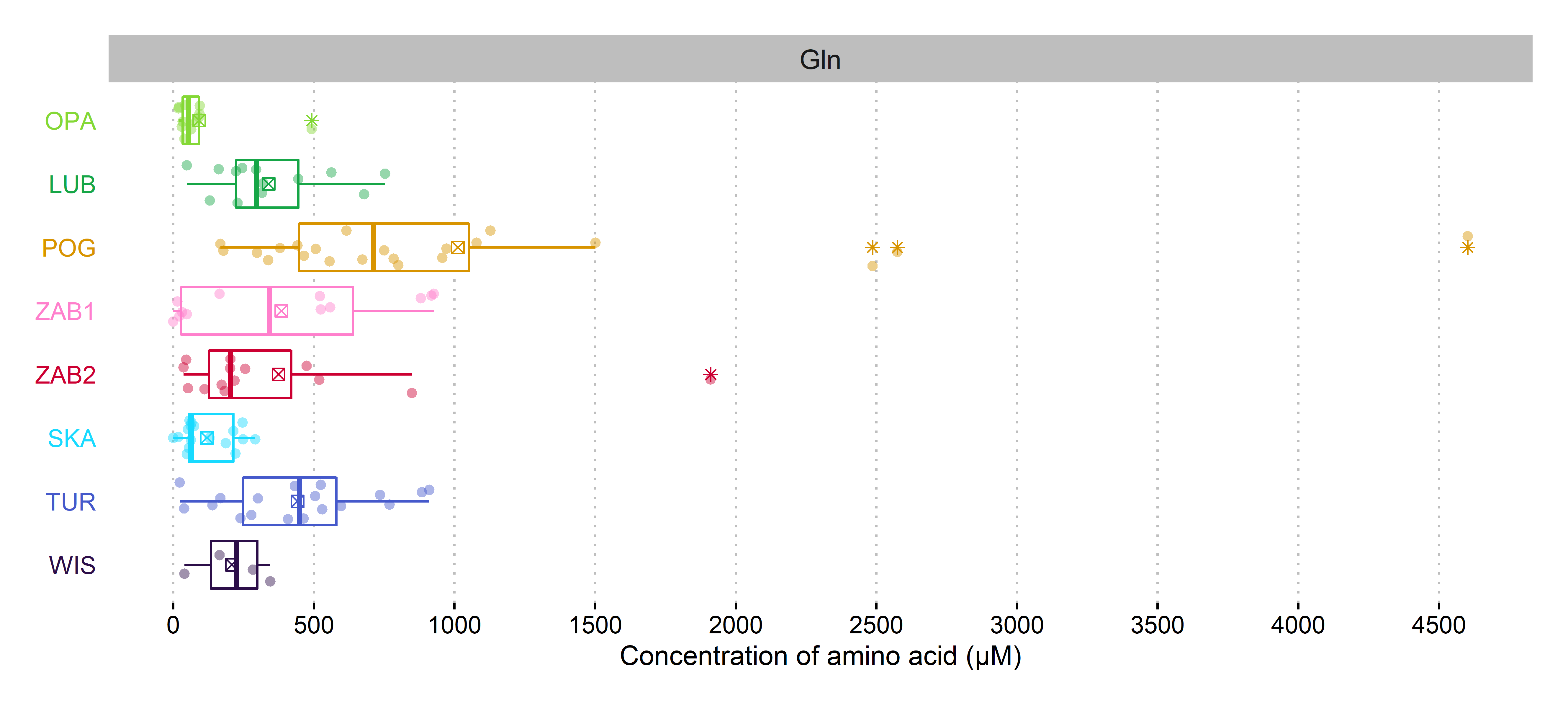

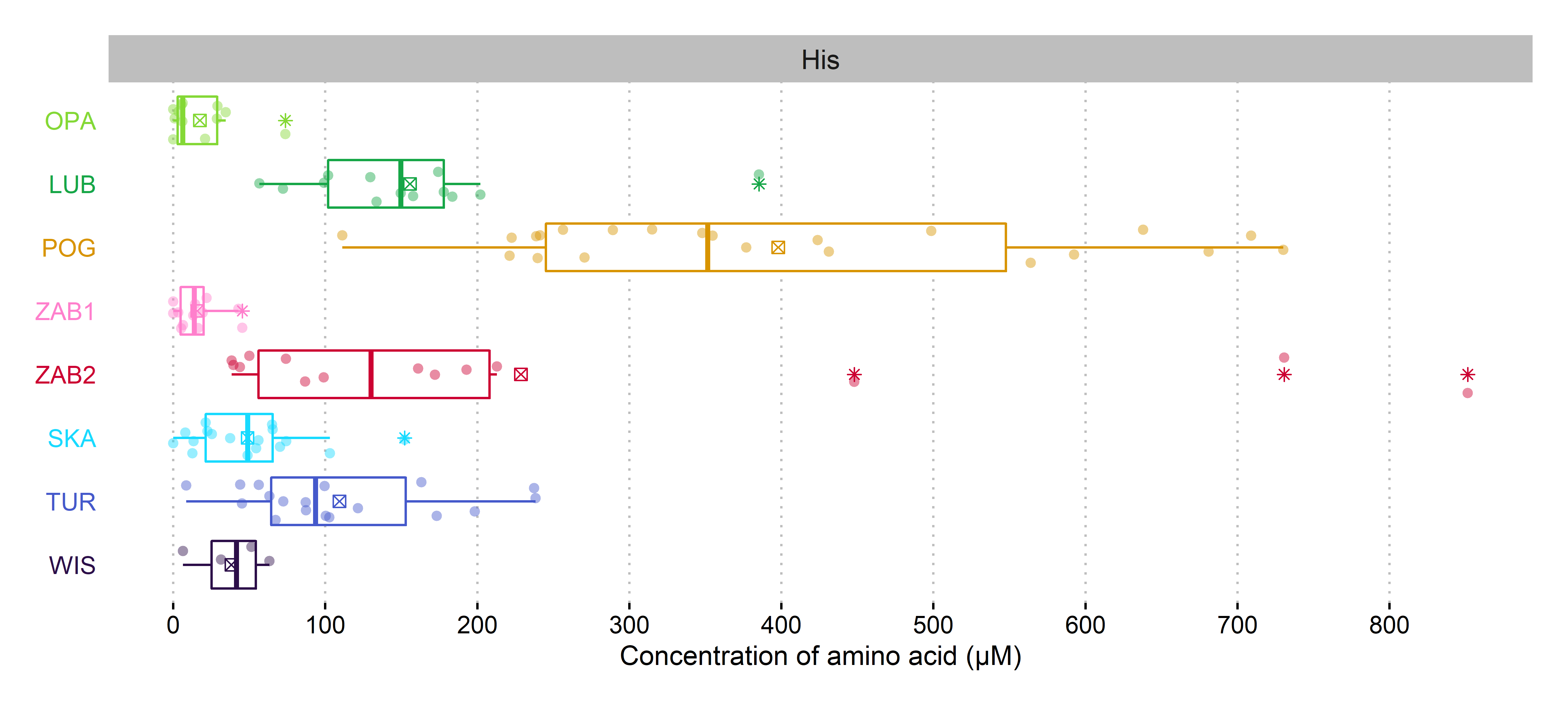

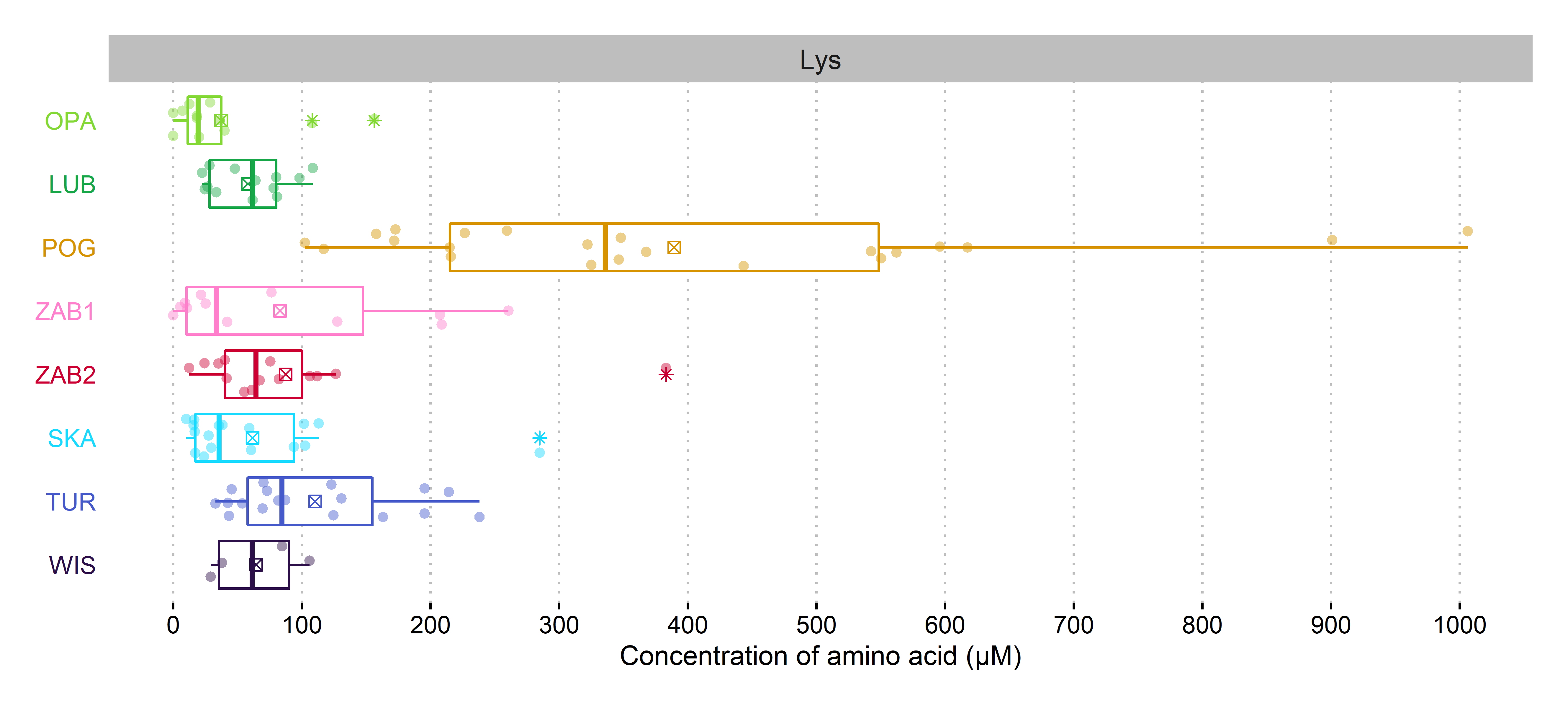

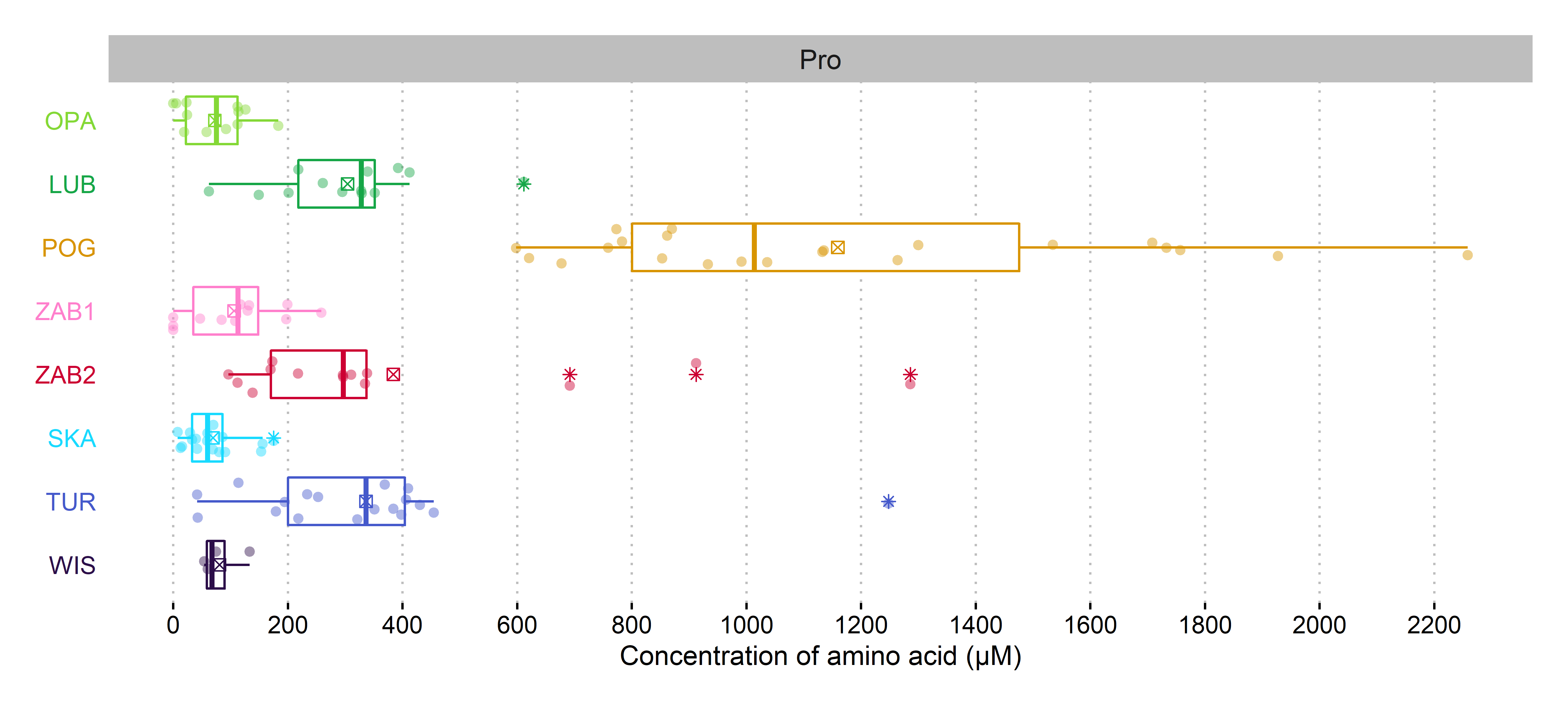

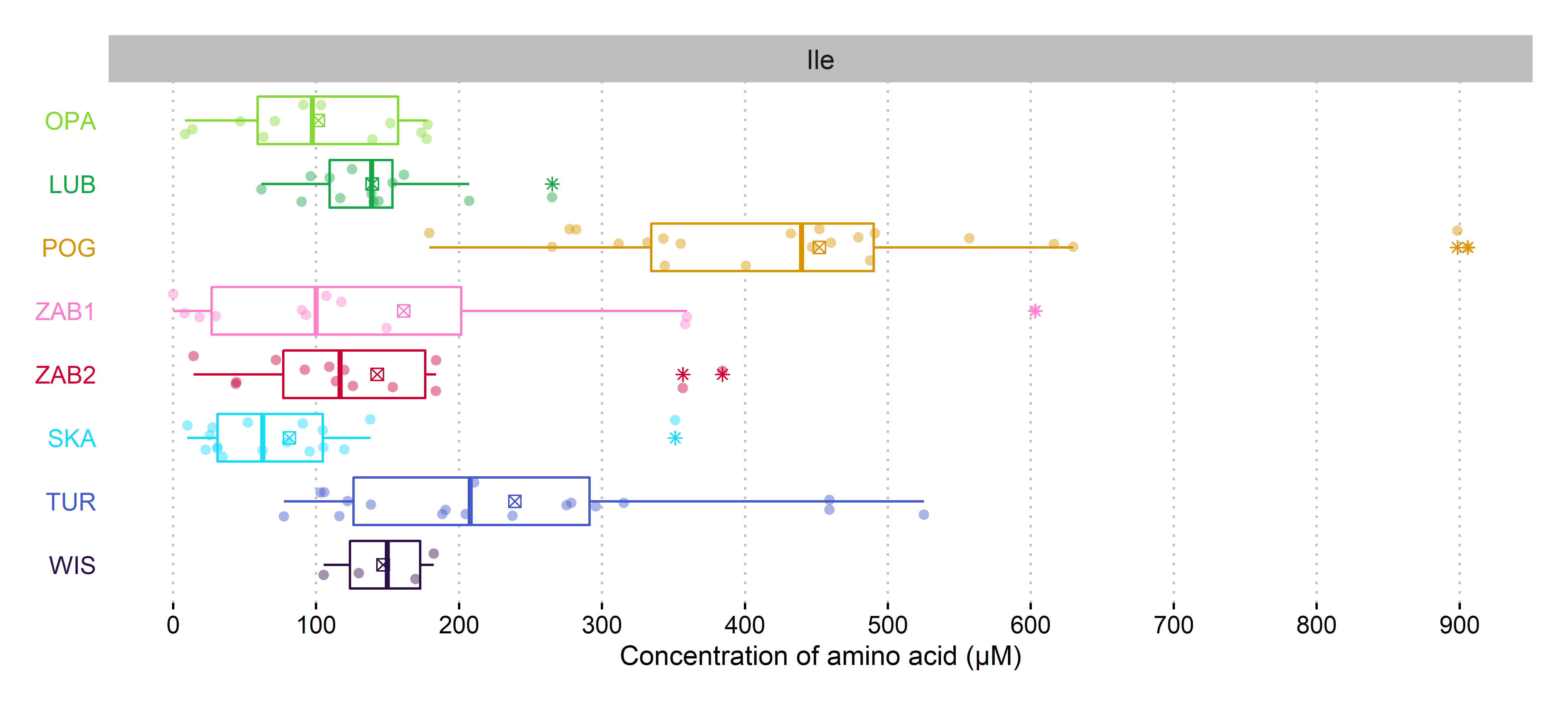

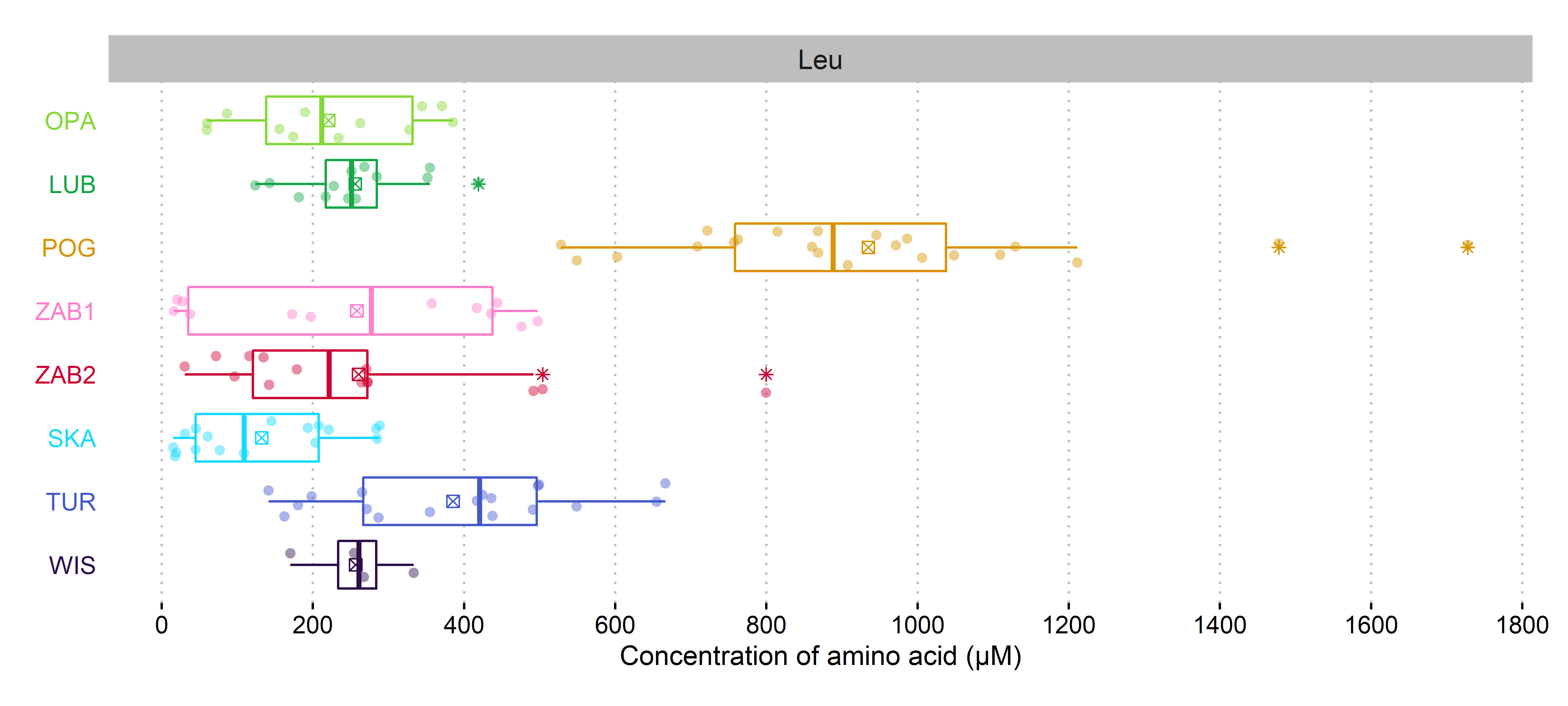

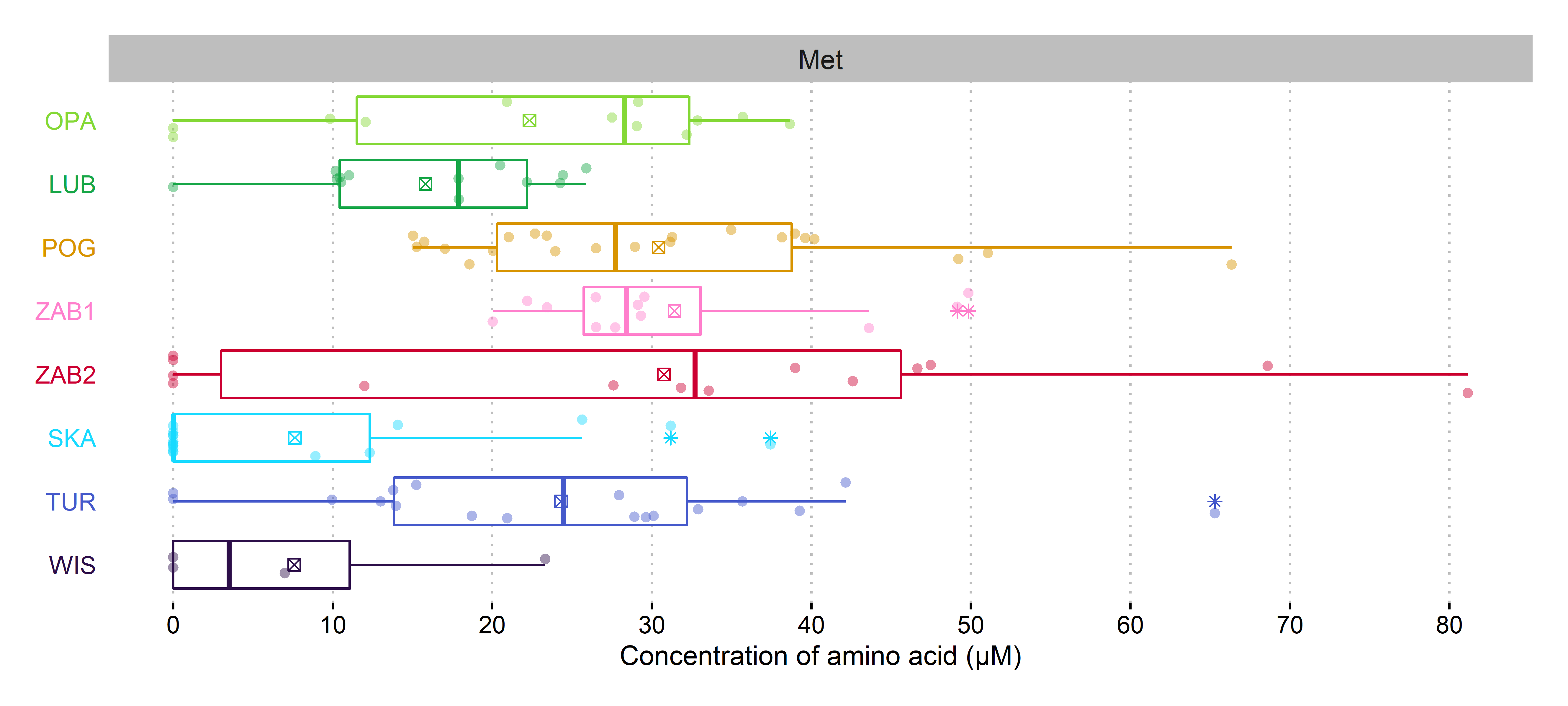

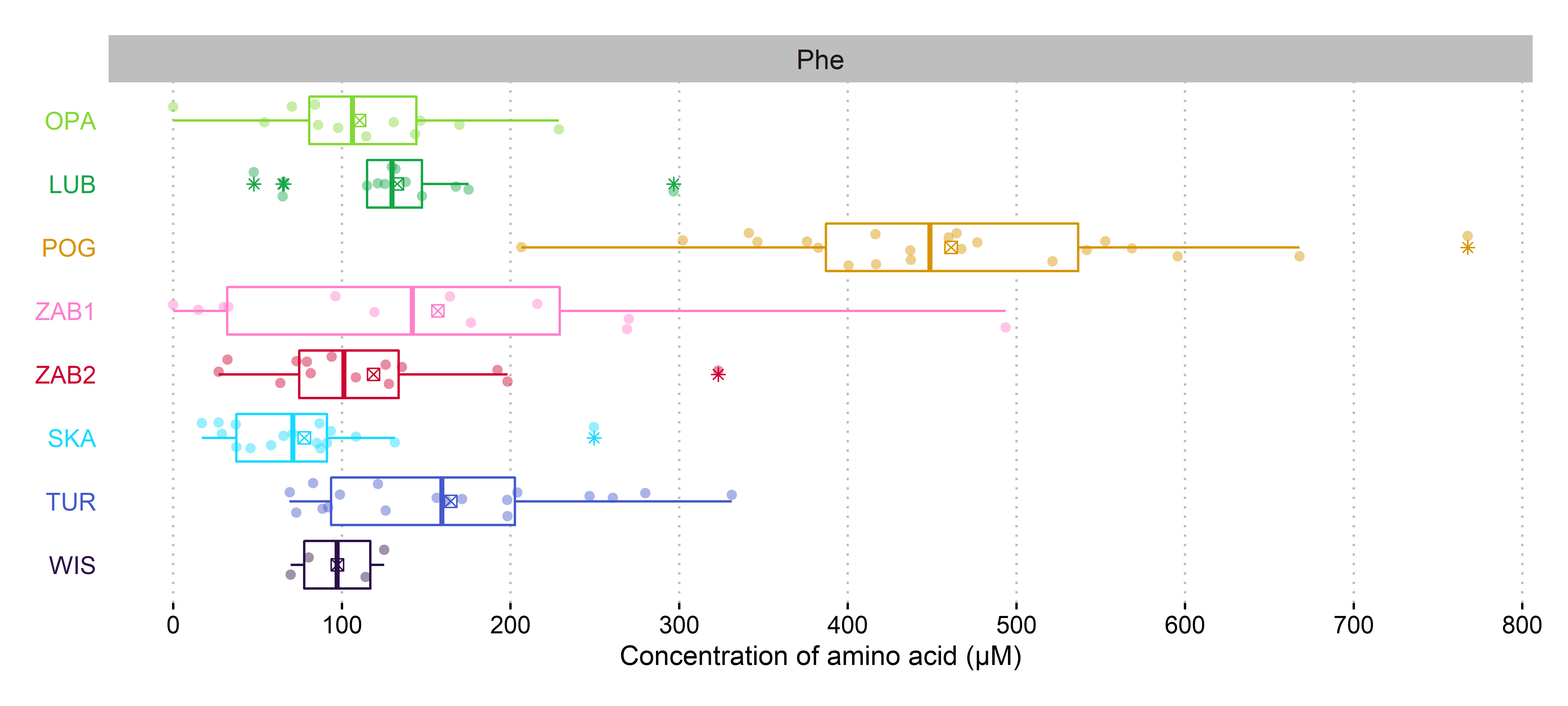

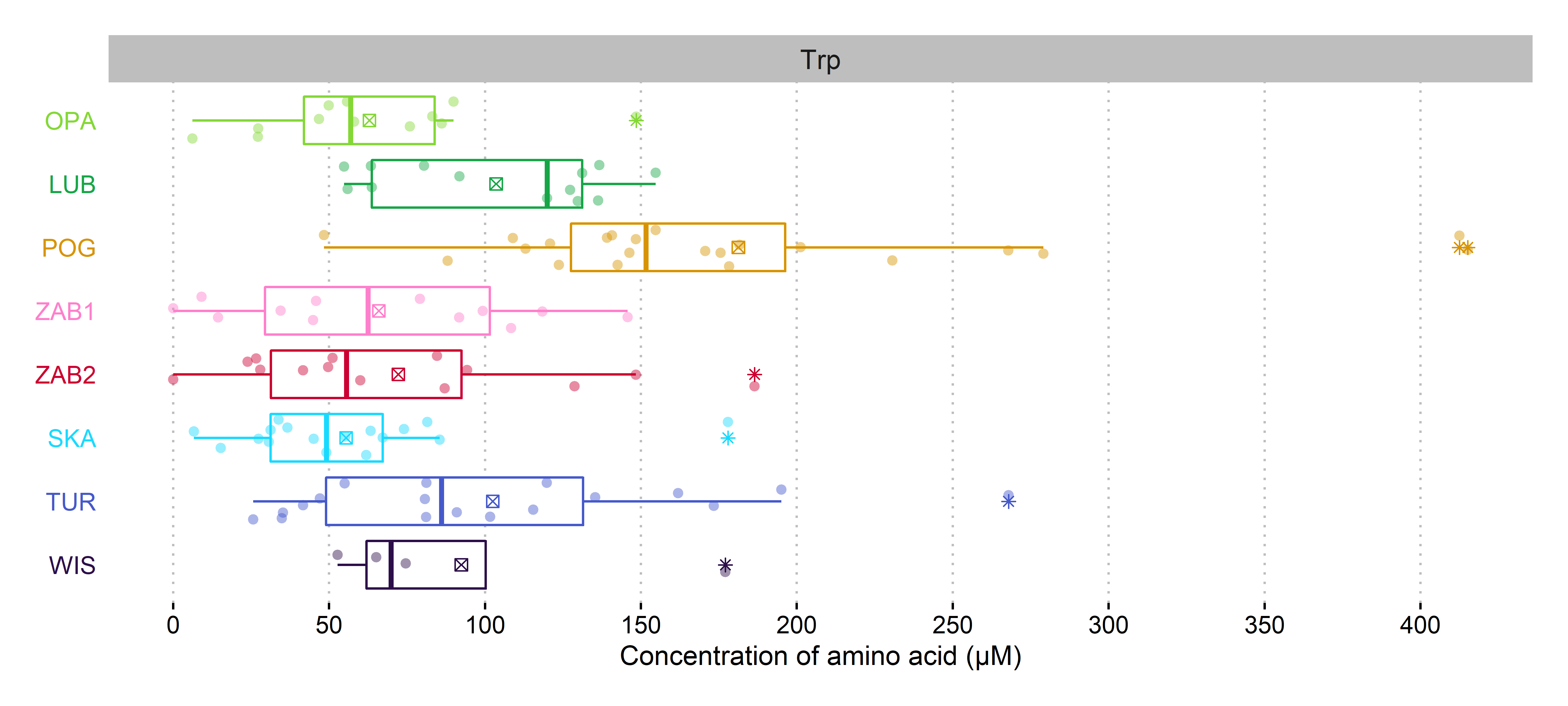

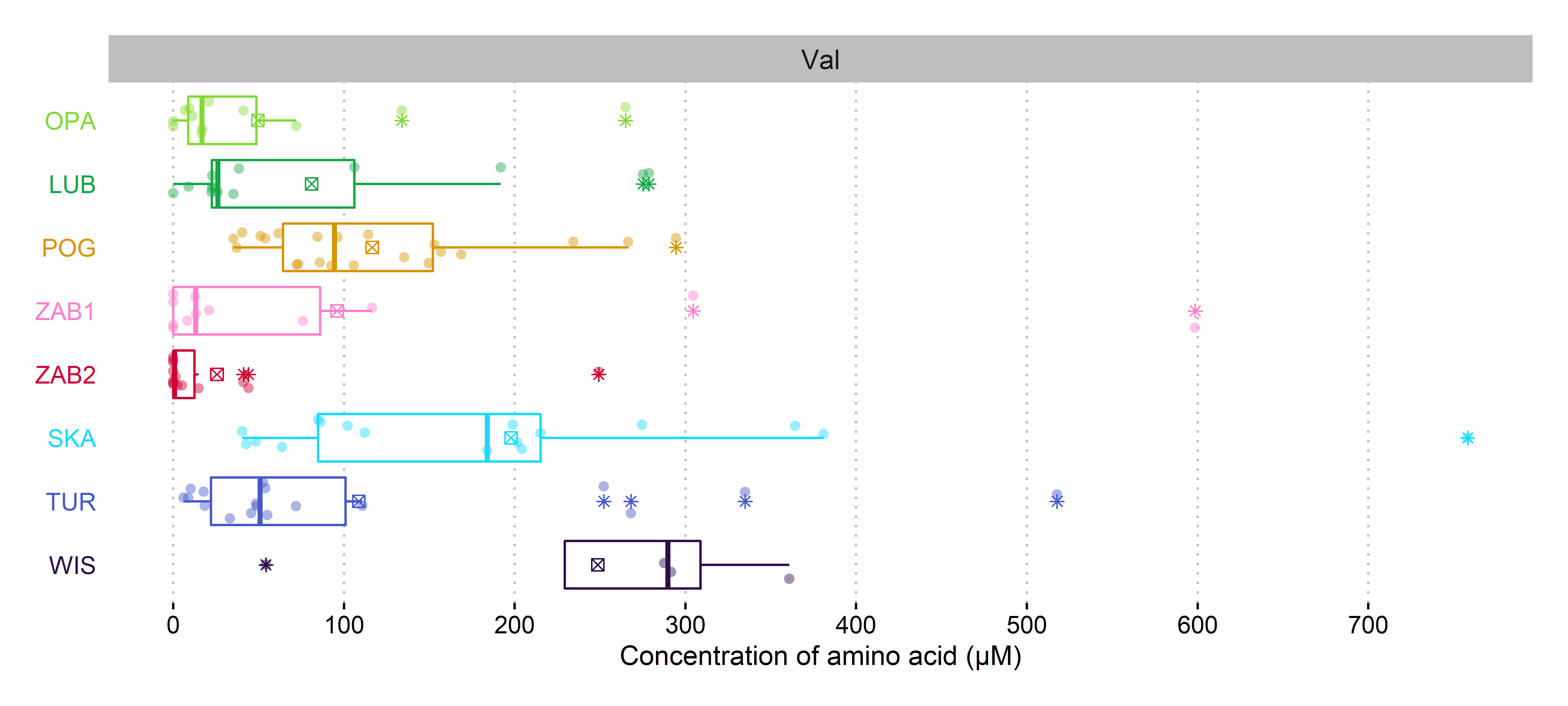

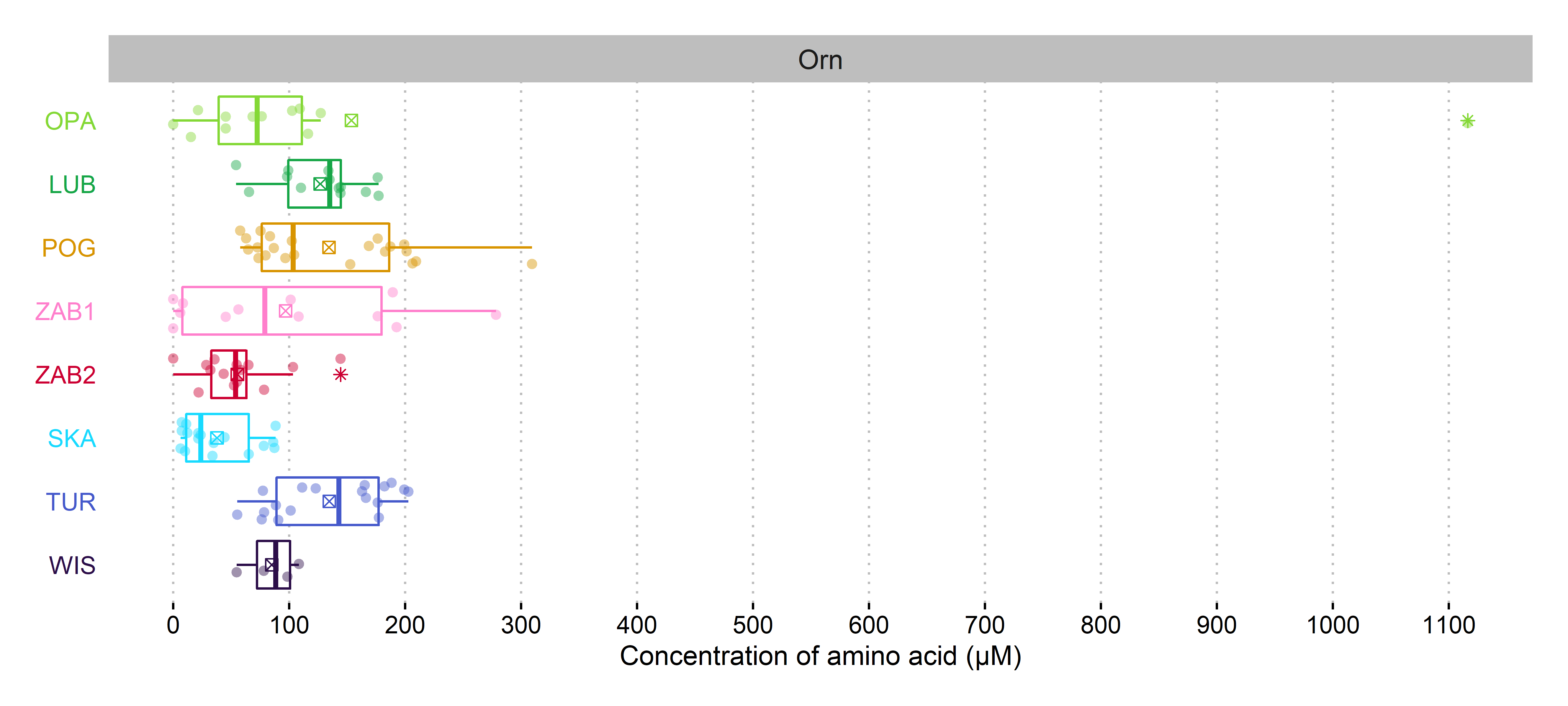

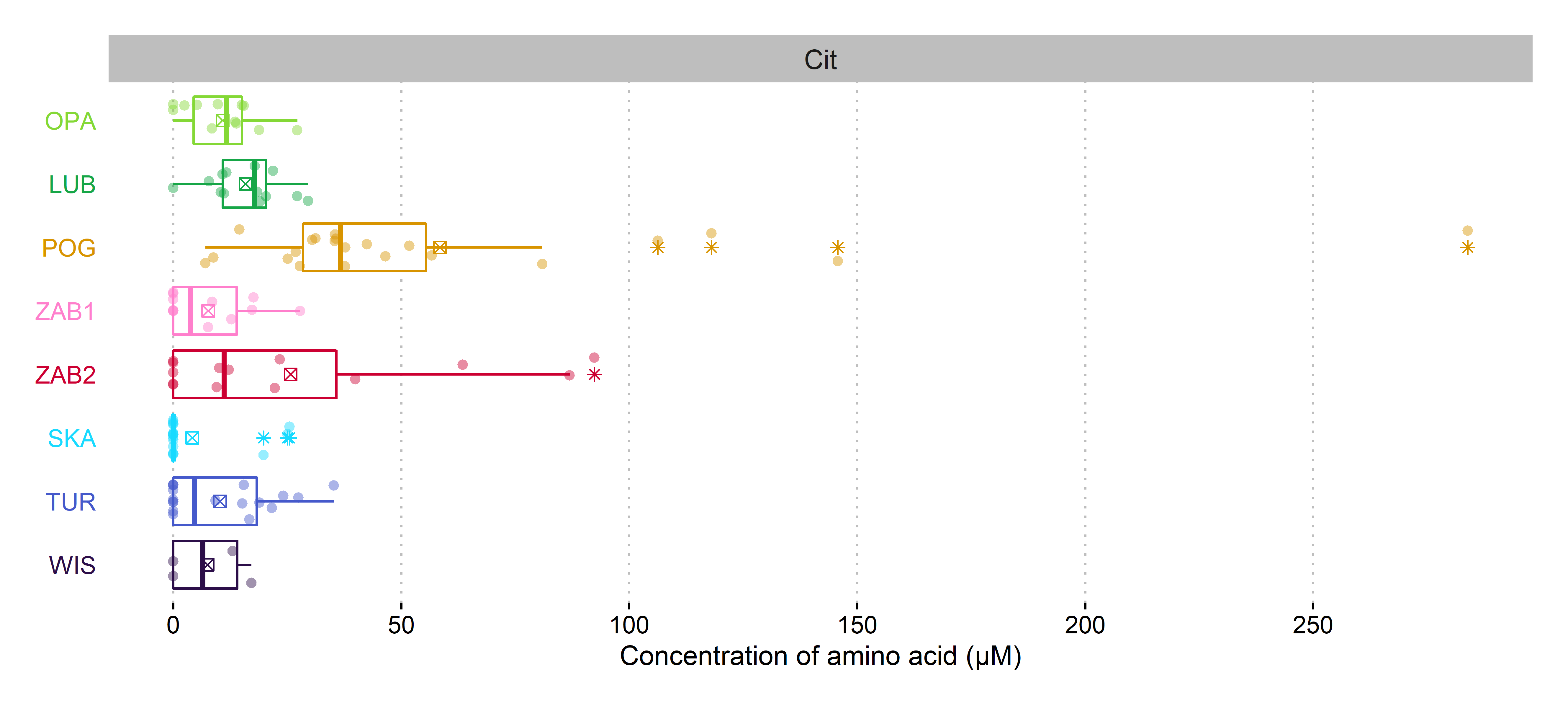

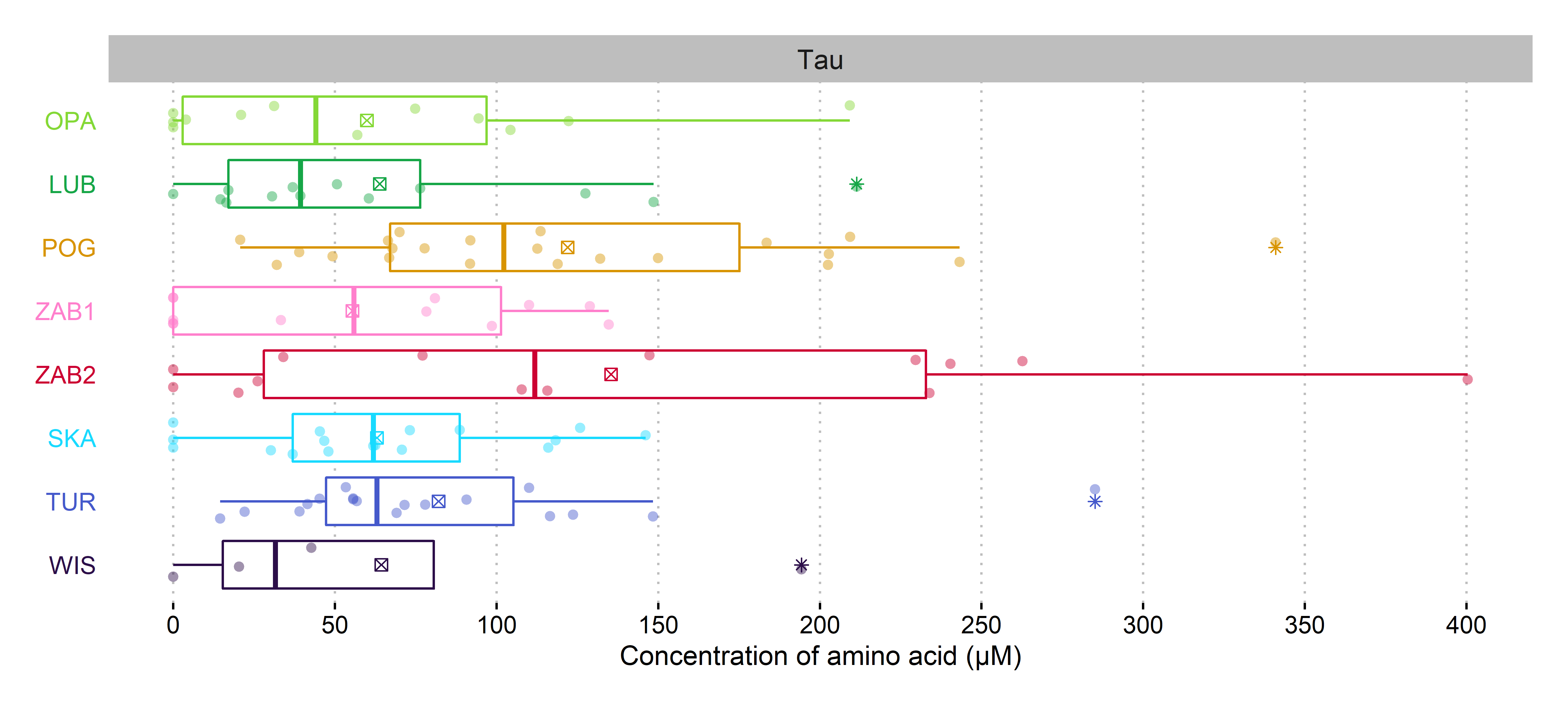

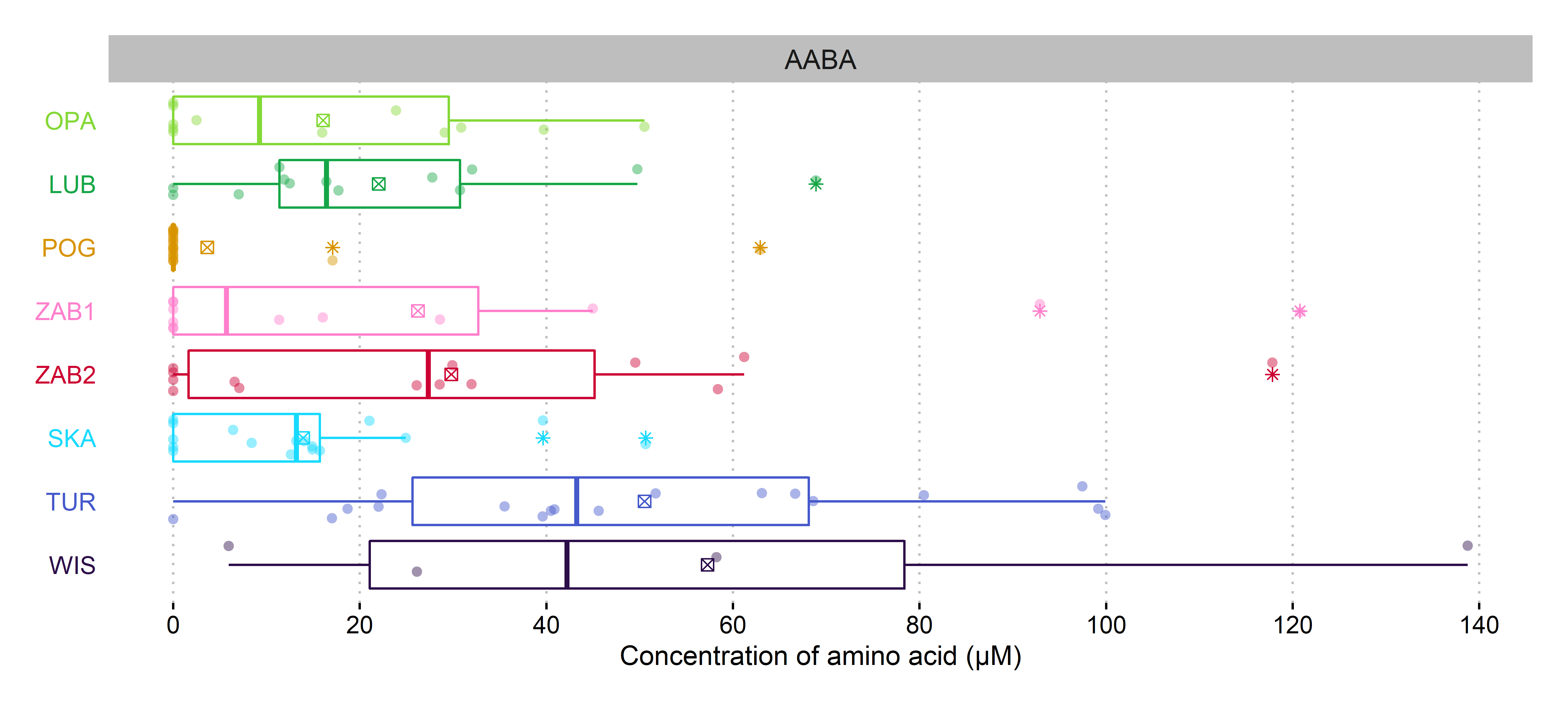

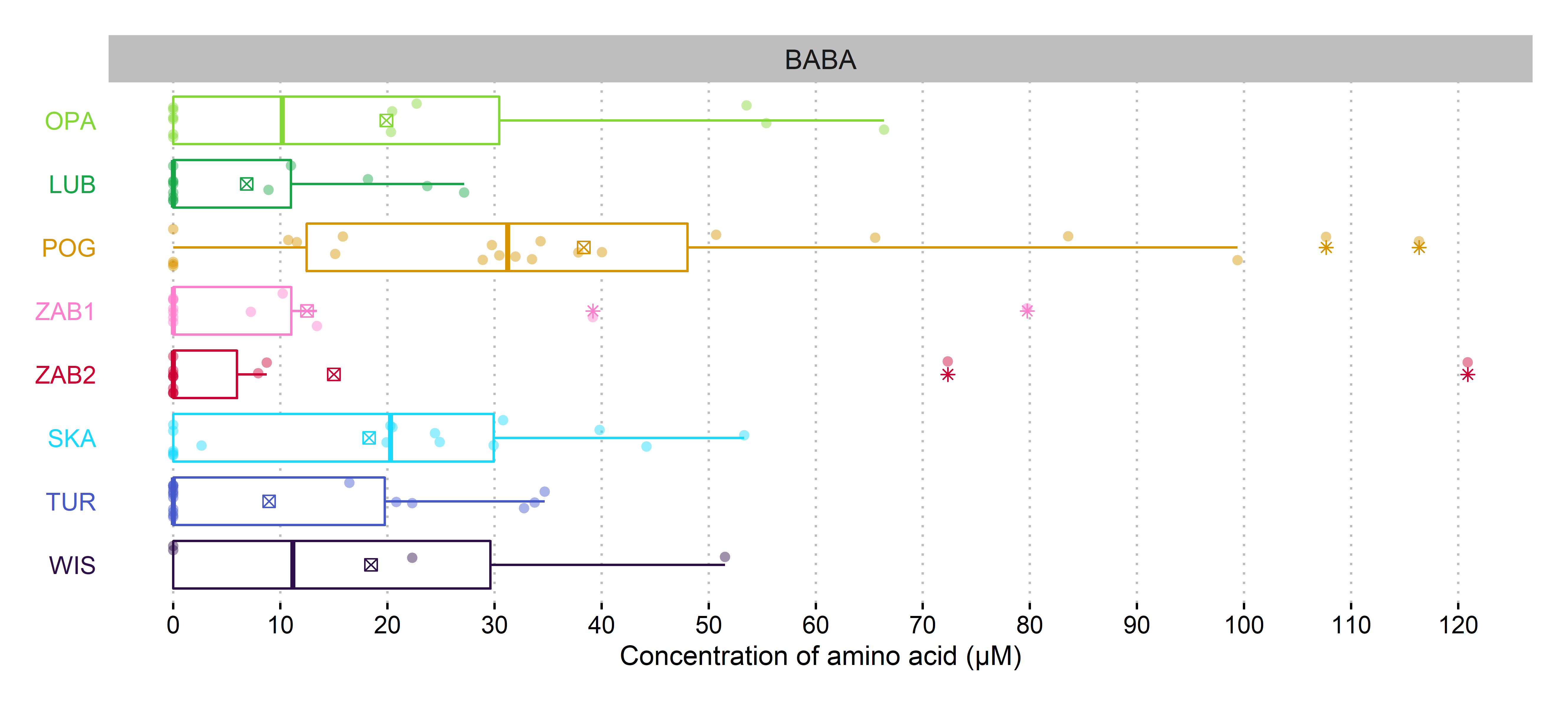

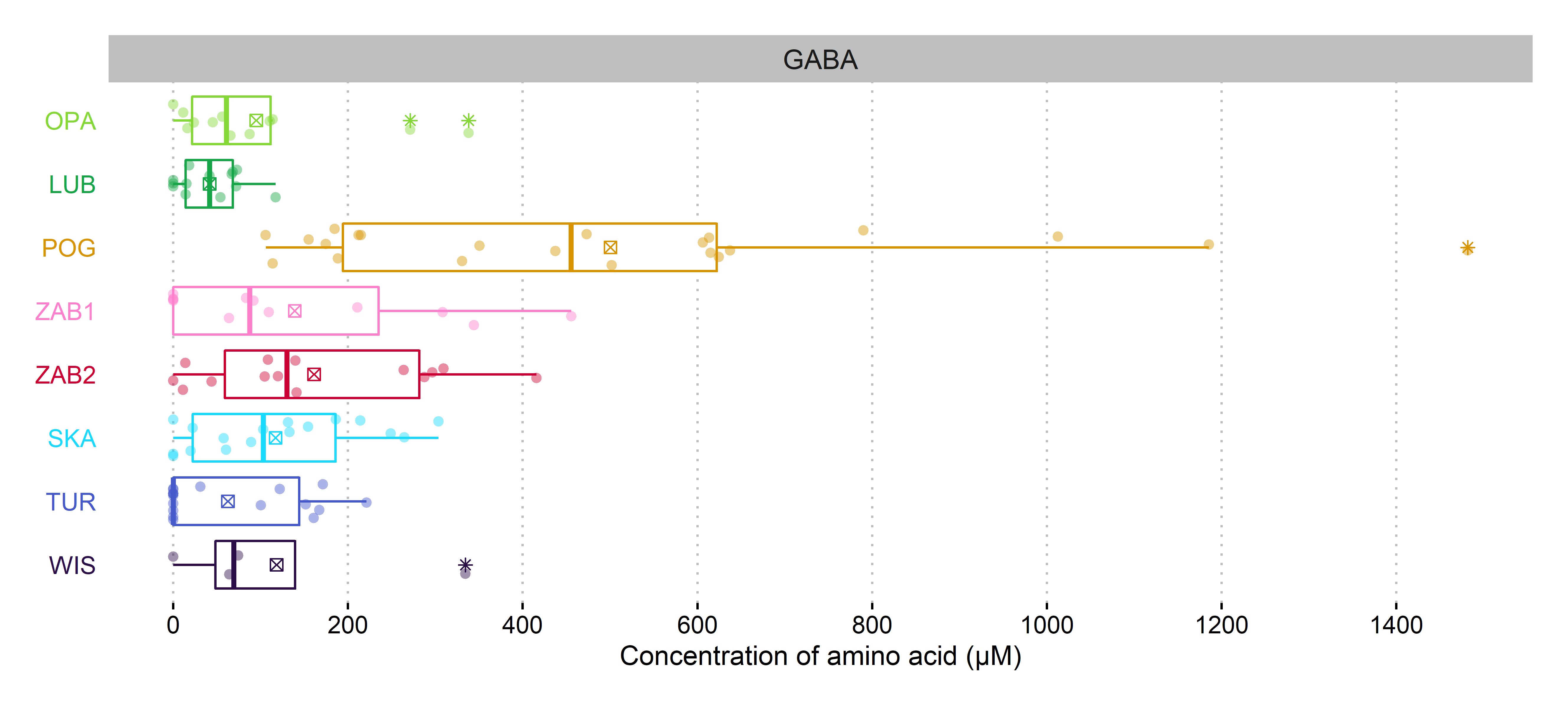

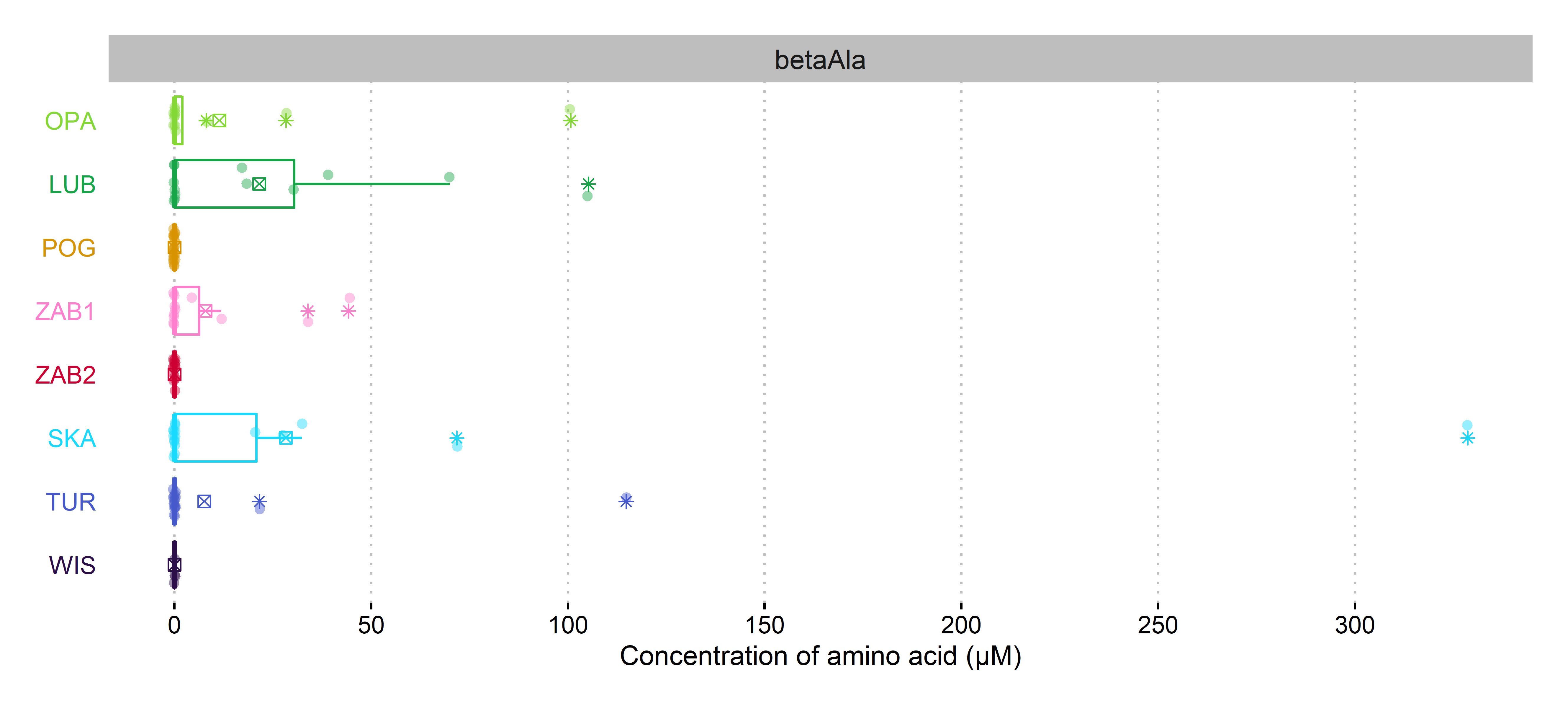

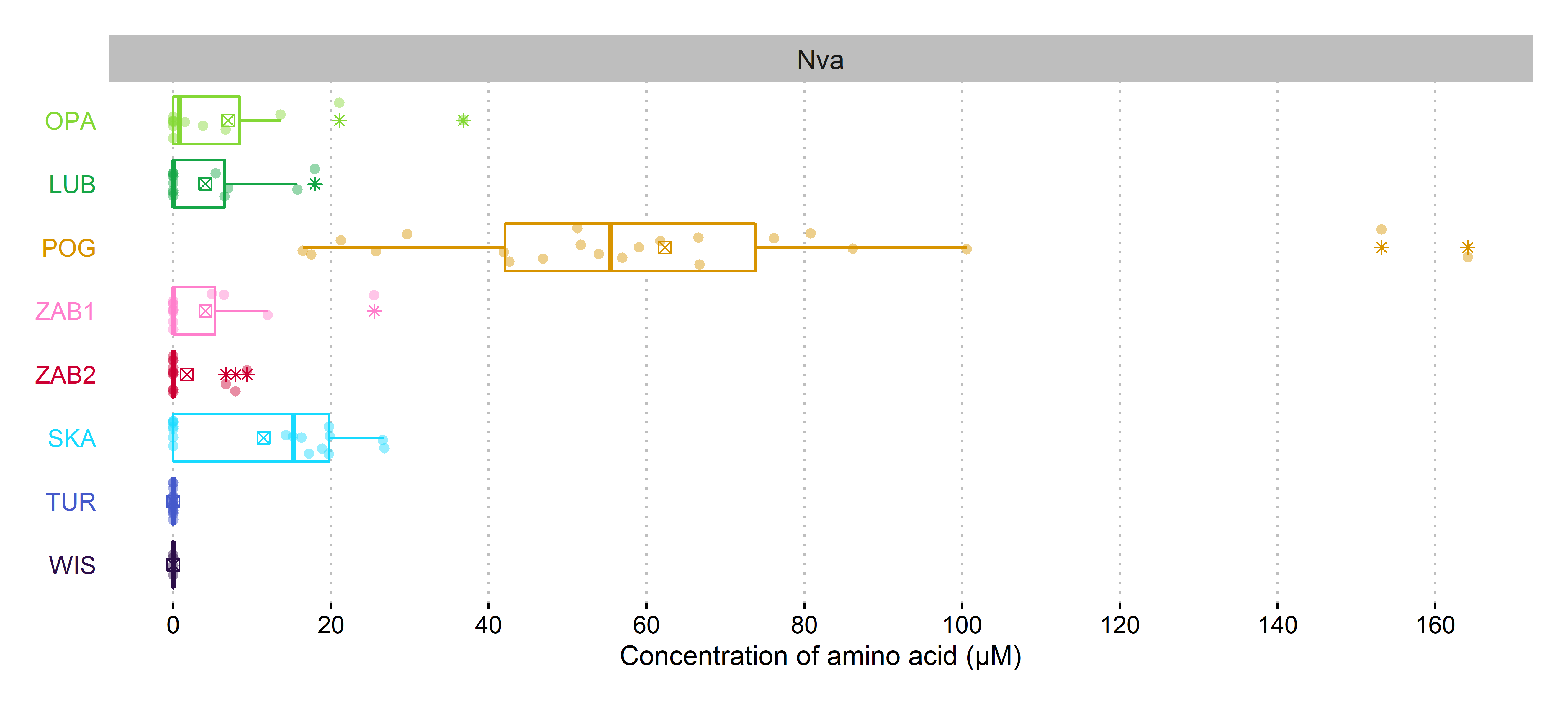


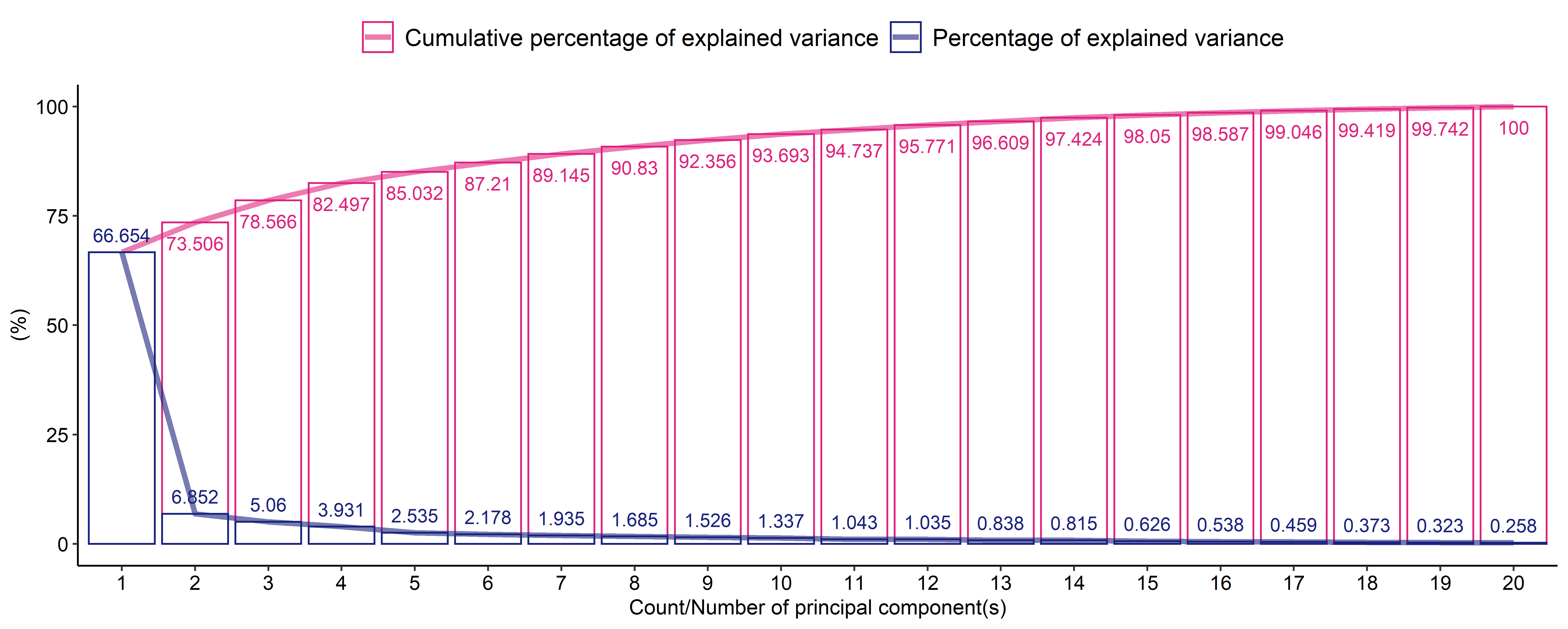


**Figure S2.** Scree plot showing the proportion of explained variance by the principal components.


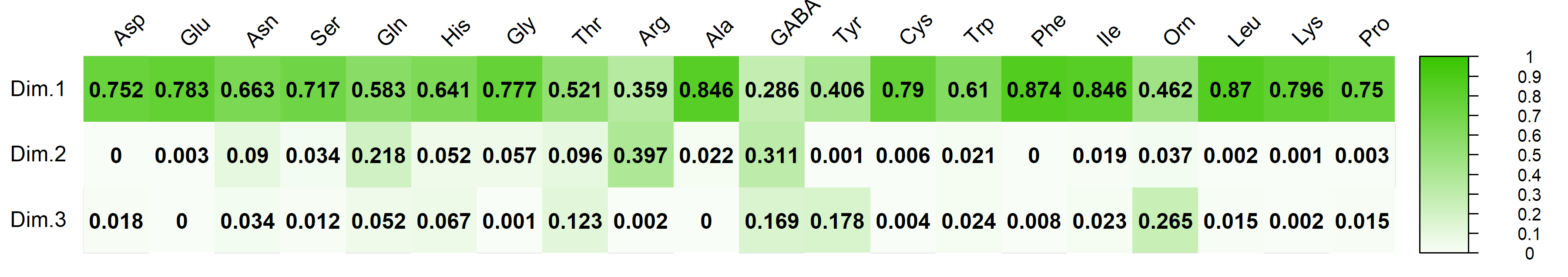


**Figure S3.** Cos^2^ for the amino acids selected as active variables in the principal component analysis model, representing the quality of representation for variables on the factor map (Dim1-3).


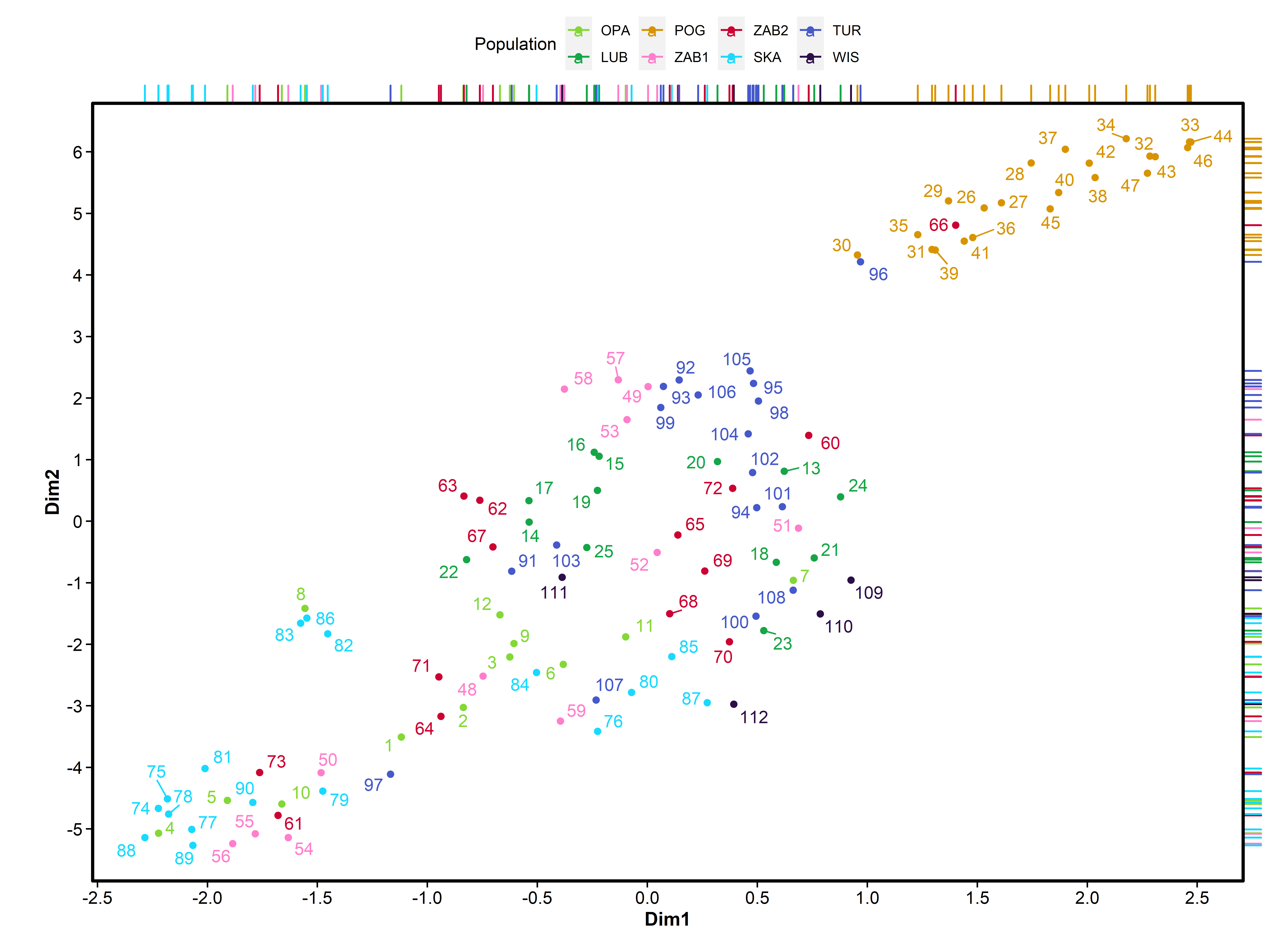


**Figure S4.** Uniform manifold approximation and projection of all amino acids in *Neottia ovata* populations, except for β-Ala. Individuals (populations) are color-coded and labeled with a number corresponding to Id used in Table S3.
