# Supplementary material for: How Are the Flower Structure and Nectar Composition of the Generalistic Orchid Neottia ovata Adapted to a Wide Range of Pollinators?
Source: Int J Mol Sci. 2021 Feb 23;22(4):2214. doi: 10.3390/ijms22042214 (PMC7926835; doi:10.3390/ijms22042214)
Supplement: Supplementary file 1 [file ijms-22-02214-s001.zip › Supplementary materials - Tables S1-2 and Figures S1-4.pdf]

Table S1. Amino acids transformation using Tukey's Ladder of Power:

$$y = \begin{Bmatrix} x^{\lambda} & , if\ \lambda > 0 \\ log(x) & , if\ \lambda = 0 \\ -(x^{\lambda}) & , if\ \lambda < 0 \end{Bmatrix}$$, where y – new value, x – original value.

Amino acid	Number of iterations	λ	W statistic	*p*-value
Asp	2078	0.385	0.9857	0.2798
Glu	2042	0.205	0.9822	0.1411
Asn	2048	0.235	0.9867	0.3380
Ser	2038	0.185	0.9811	0.1139
Gln	2055	0.270	0.9795	0.08244
His	2063	0.310	0.9863	0.3146
Gly	2065	0.320	0.9686	0.009655
Thr	2079	0.390	0.9756	0.03763
Cit	2083	0.410	0.8806	5.183e-08
Arg	2090	0.445	0.9517	0.0004818
Ala	1989	-0.060	0.9839	0.1981
Tau	2111	0.550	0.9699	0.01231
GABA	2081	0.400	0.9497	0.0003495
BABA	2103	0.510	0.8189	2.054e-10
Tyr	2074	0.365	0.8103	1.053e-10
AABA	2103	0.510	0.8793	4.537e-08
Cys	2020	0.095	0.9920	0.7597
Val	2075	0.370	0.9718	0.01793
Met	2167	0.830	0.9497	0.0003468
Nva	2075	0.370	0.7778	9.977e-12
Trp	2092	0.455	0.9845	0.2221
Phe	2078	0.385	0.9725	0.02038
Ile	2070	0.345	0.9882	0.4378
Orn	2078	0.385	0.9294	1.682e-05
Leu	2062	0.305	0.9868	0.3403
Lys	2059	0.290	0.9722	0.01927
Pro	2061	0.300	0.9819	0.134

Table S2. Kaiser-Meyer-Olkin test results sorted in descending order by the measure of sampling adequacy (MSA) (overall MSA = 0.92).

Amino acid	MSA	Variable type
Asp	0.95	Active
Glu	0.95	Active
Asn	0.95	Active
Ser	0.95	Active
Cys	0.95	Active
Trp	0.95	Active
Ile	0.95	Active
Orn	0.95	Active
Leu	0.95	Active
Lys	0.95	Active
Ala	0.94	Active
Phe	0.94	Active
Gly	0.93	Active
Cit	0.93	Active
Tyr	0.93	Active
Pro	0.93	Active
His	0.90	Active
Gln	0.88	Active
Thr	0.88	Active
GABA	0.88	Active
Nva	0.86	Supplementary
Arg	0.85	Active
Met	0.74	Supplementary
BABA	0.69	Supplementary
Val	0.62	Supplementary
AABA	0.54	Supplementary
Tau	0.53	Supplementary

Table S3. Amino acids dataset used in PCA and UMAP analyses.

Dataset is in Table3S.xlsx file.

Figure S1. Boxplots of amino acids concentration for *Neottia ovata* populations. Colored dots are individual samples. The crossed square shows the mean. The lower and upper hinges correspond to the lower (Q_1) and upper (Q_3) quartiles. Thus box length shows the interquartile range (IQR). The thicker line inside boxes corresponds to the median. The lower whisker extends from the hinge to the smallest value at most Q_1 - 1.5 × IQR of the hinge. The upper whisker extends from the hinge to the largest value no further than Q_3 + 1.5 × IQR. Data beyond the end of the whiskers, indicated with an asterisk symbol, are outliers.

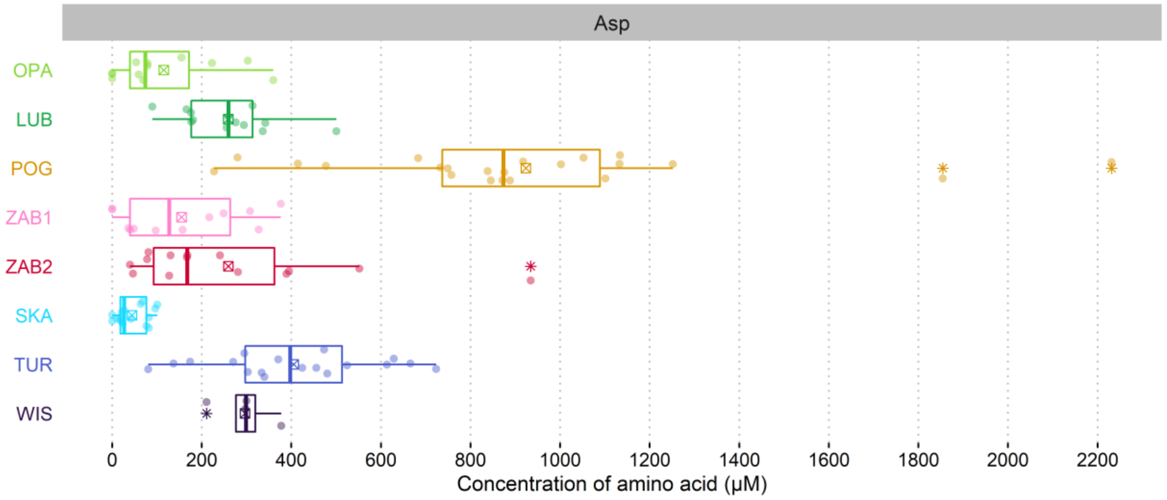

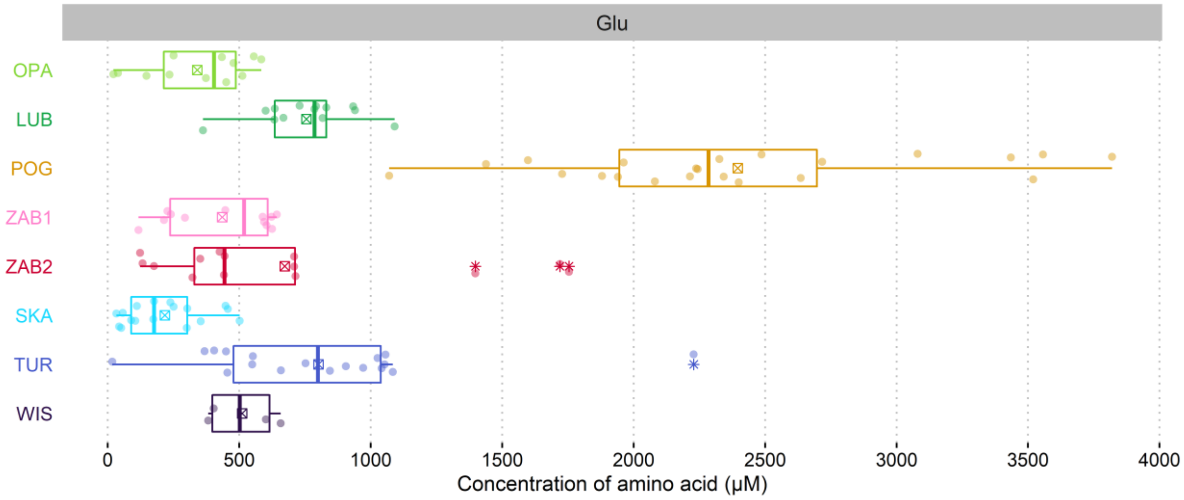

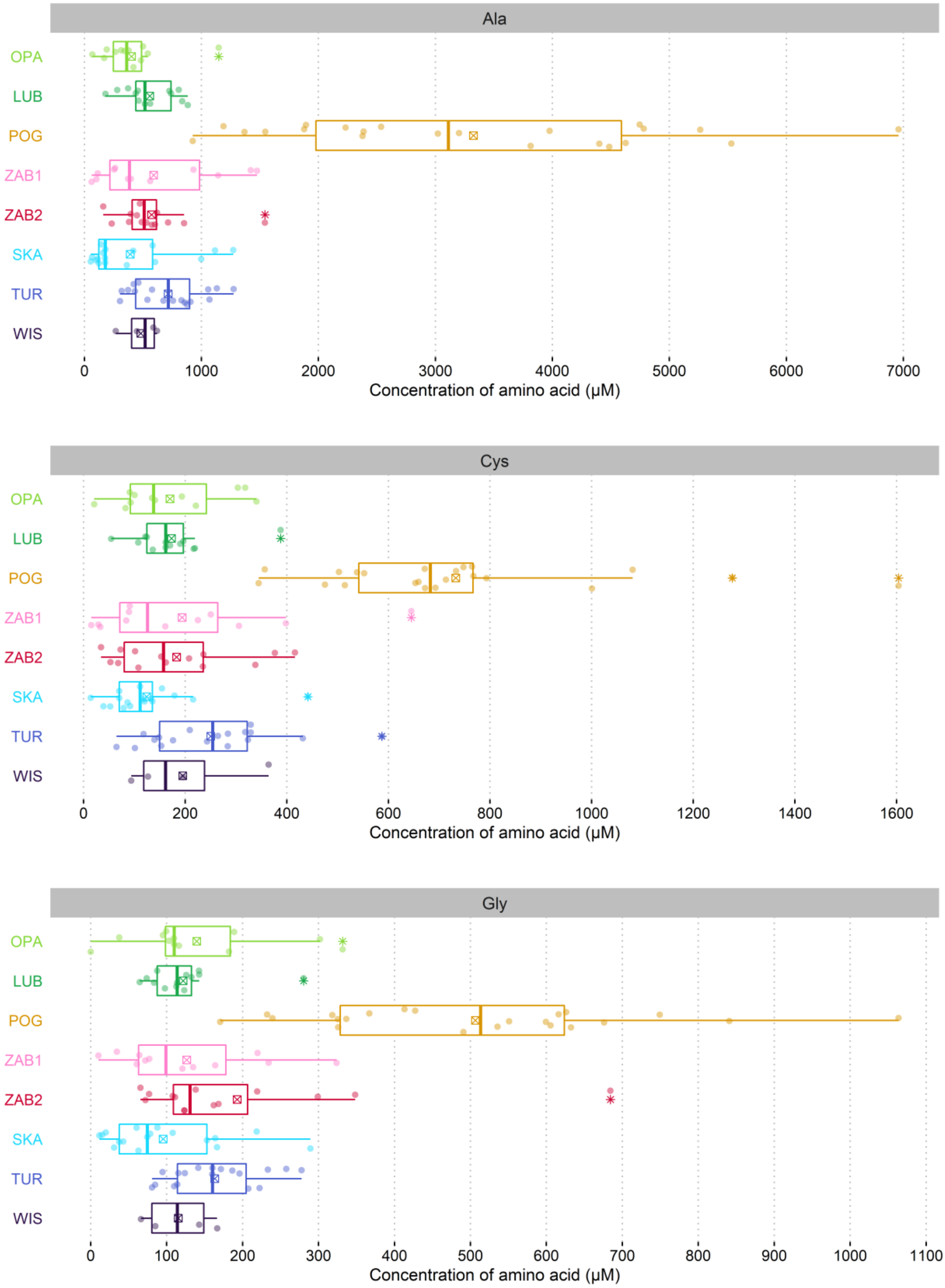
Ala
OPA
LUB
POG
ZAB1
ZAB2
SKA
TUR
WIS
0
1000
2000
3000
4000
5000
6000
7000
Concentration of amino acid (µM)
Cys
OPA
LUB
POG
ZAB1
ZAB2
SKA
TUR
WIS
0
200
400
600
800
1000
1200
1400
1600
Concentration of amino acid (µM)
Gly
OPA
LUB
POG
ZAB1
ZAB2
SKA
TUR
WIS
0
100
200
300
400
500
600
700
800
900
1000
1100
Concentration of amino acid (µM)

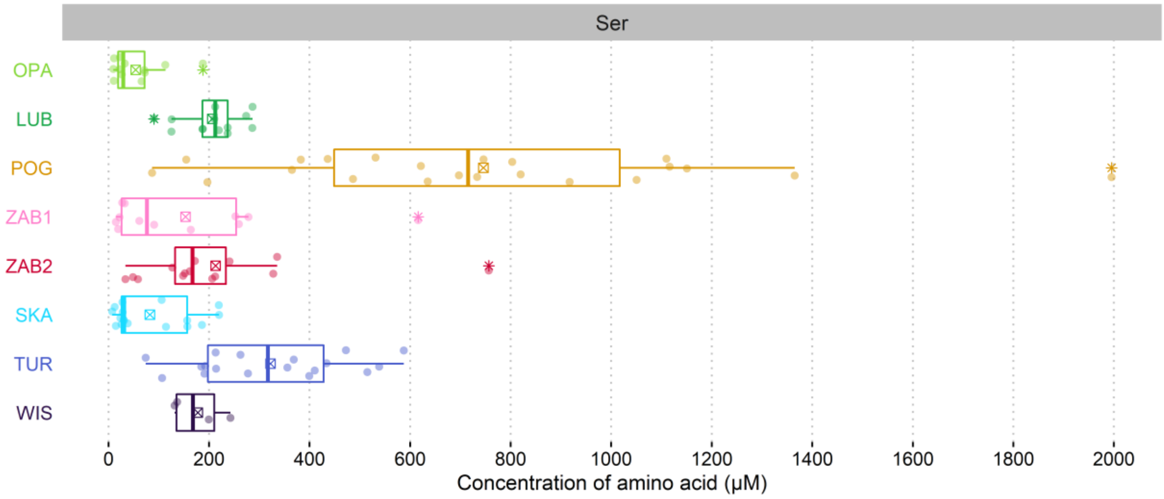
Ser
OPA
LUB
POG
ZAB1
ZAB2
SKA
TUR
WIS
0
200
400
600
800
1000
1200
1400
1600
1800
2000
Concentration of amino acid (μM)

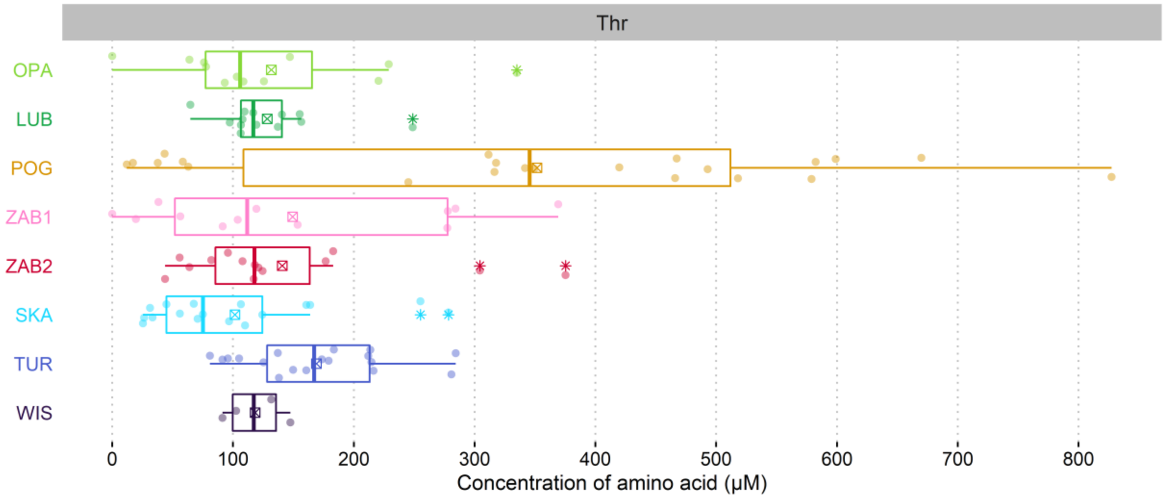
Thr
OPA
LUB
POG
ZAB1
ZAB2
SKA
TUR
WIS
0
100
200
300
400
500
600
700
800
Concentration of amino acid (μM)

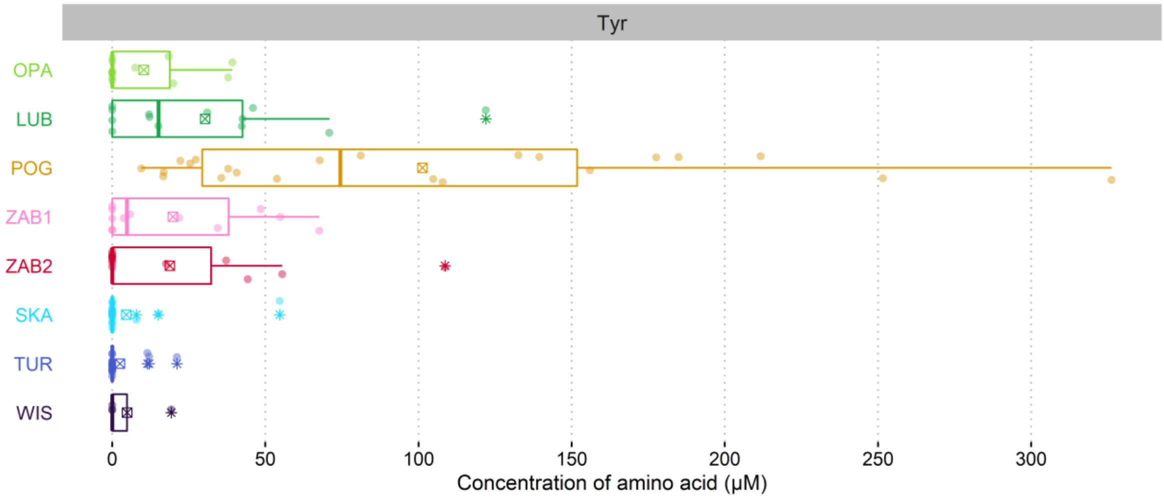
Tyr
OPA
LUB
POG
ZAB1
ZAB2
SKA
TUR
WIS
0
50
100
150
200
250
300
Concentration of amino acid (μM)

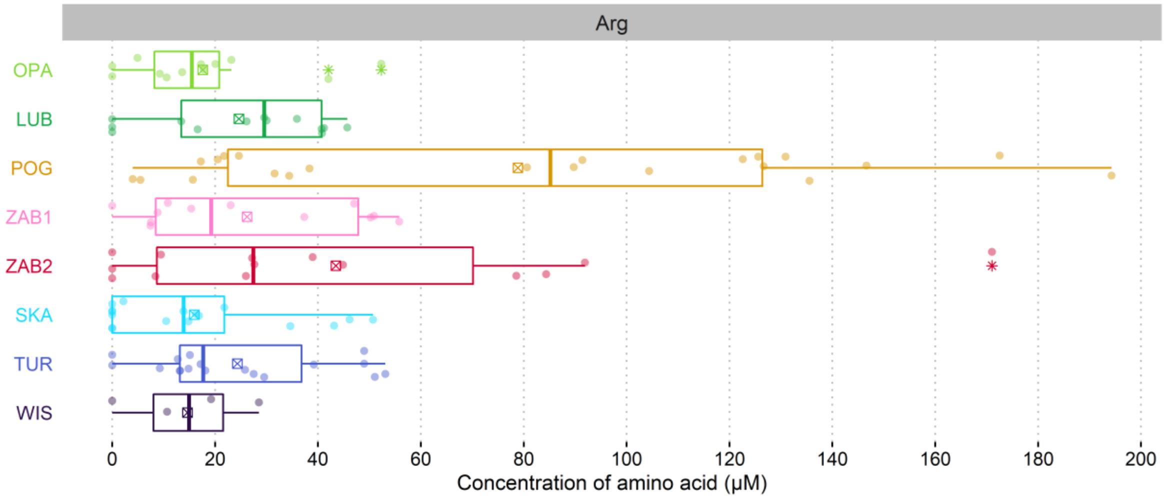
Arg
OPA
LUB
POG
ZAB1
ZAB2
SKA
TUR
WIS
0
20
40
60
80
100
120
140
160
180
200
Concentration of amino acid (µM)

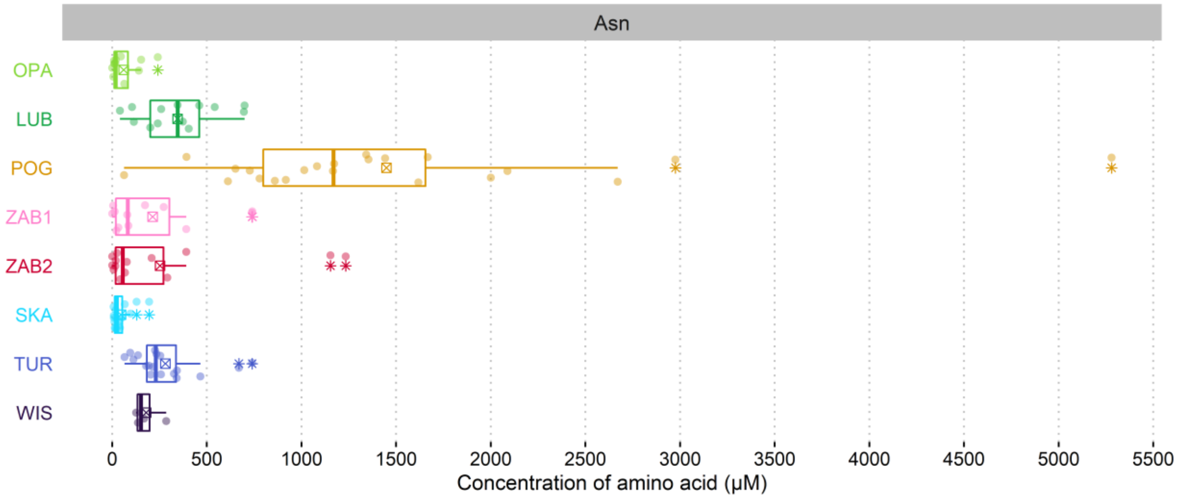
Asn
OPA
LUB
POG
ZAB1
ZAB2
SKA
TUR
WIS
0
500
1000
1500
2000
2500
3000
3500
4000
4500
5000
5500
Concentration of amino acid (µM)

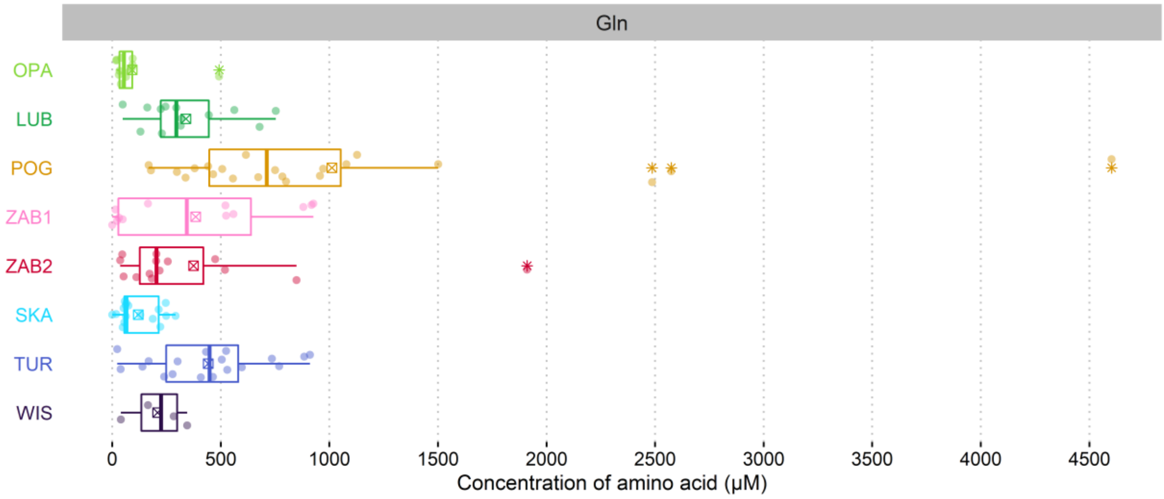
Gln
OPA
LUB
POG
ZAB1
ZAB2
SKA
TUR
WIS
0
500
1000
1500
2000
2500
3000
3500
4000
4500
Concentration of amino acid (µM)

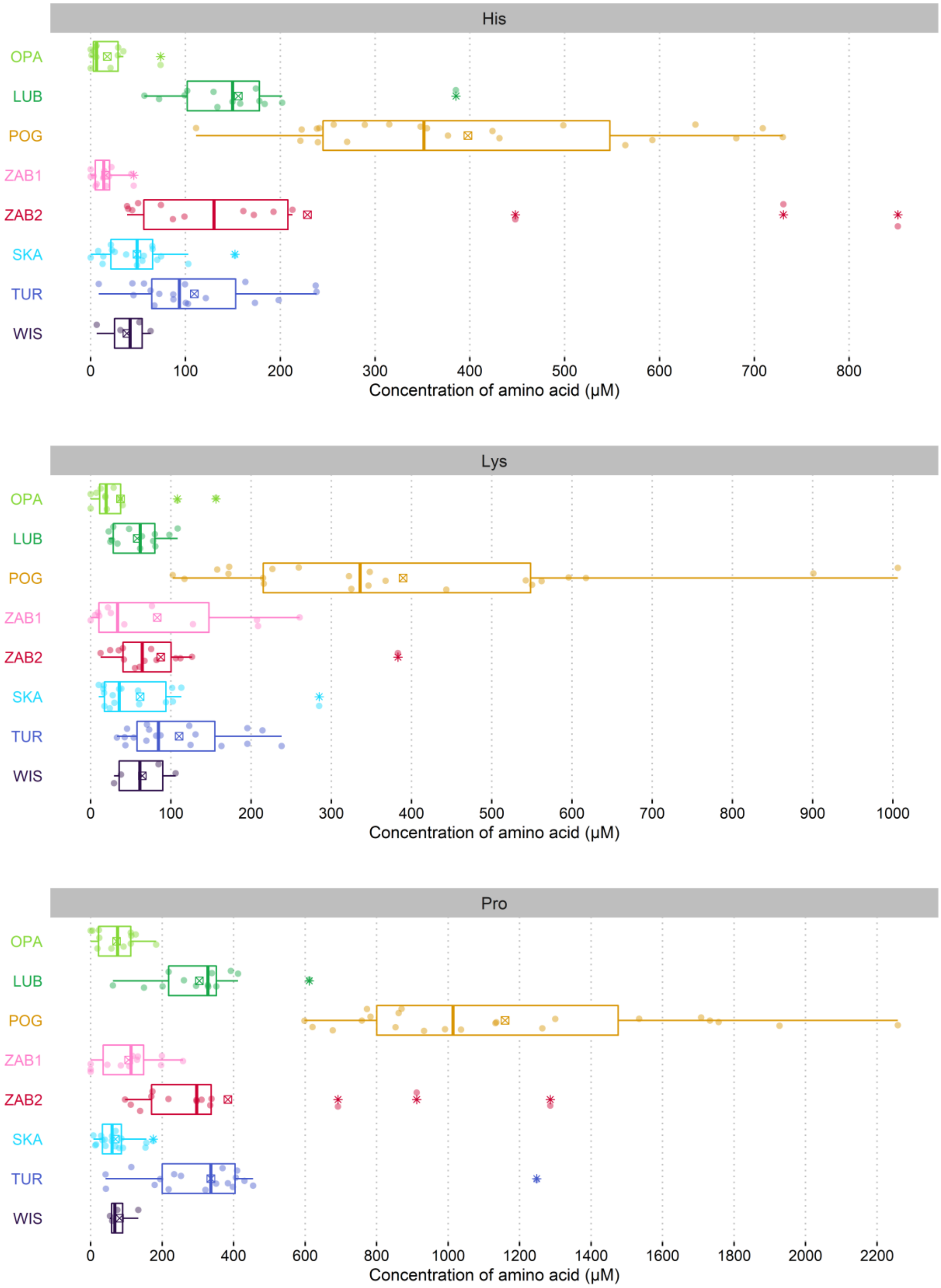
His
OPA
LUB
POG
ZAB1
ZAB2
SKA
TUR
WIS
0
100
200
300
400
500
600
700
800
Concentration of amino acid (µM)
Lys
OPA
LUB
POG
ZAB1
ZAB2
SKA
TUR
WIS
0
100
200
300
400
500
600
700
800
900
1000
Concentration of amino acid (µM)
Pro
OPA
LUB
POG
ZAB1
ZAB2
SKA
TUR
WIS
0
200
400
600
800
1000
1200
1400
1600
1800
2000
2200
Concentration of amino acid (µM)

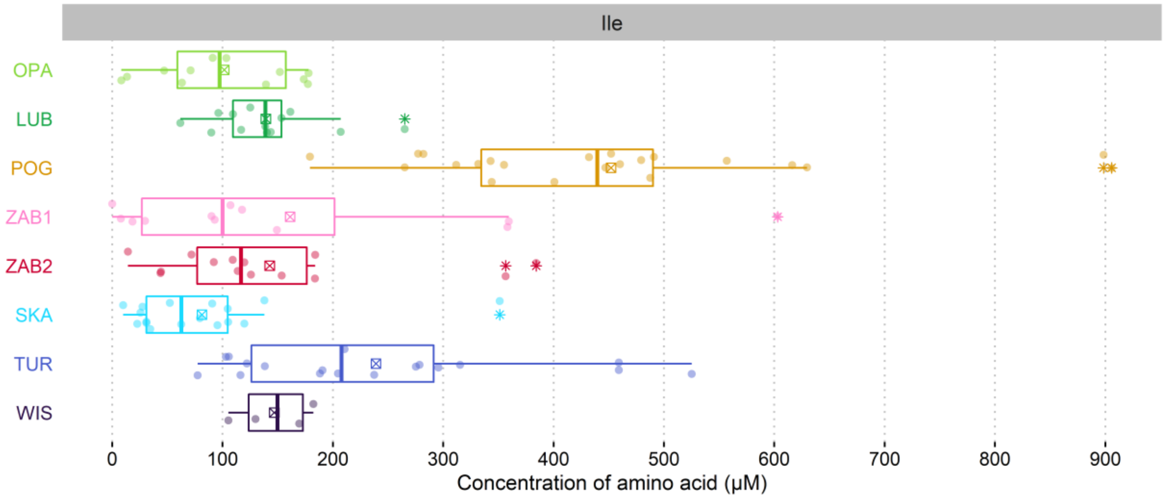
Ile
OPA
LUB
POG
ZAB1
ZAB2
SKA
TUR
WIS
0
100
200
300
400
500
600
700
800
900
Concentration of amino acid (µM)

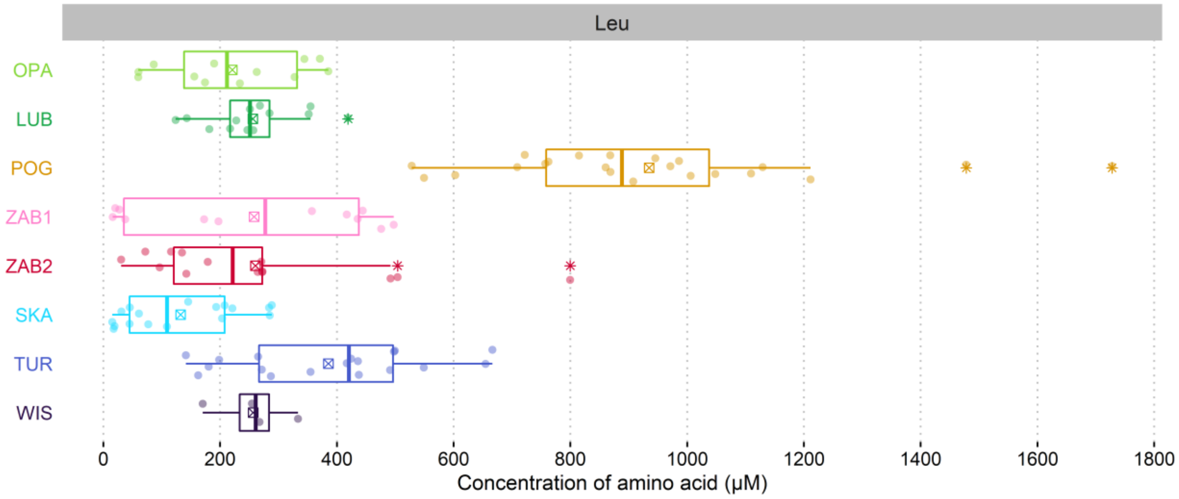
Leu
OPA
LUB
POG
ZAB1
ZAB2
SKA
TUR
WIS
0
200
400
600
800
1000
1200
1400
1600
1800
Concentration of amino acid (µM)

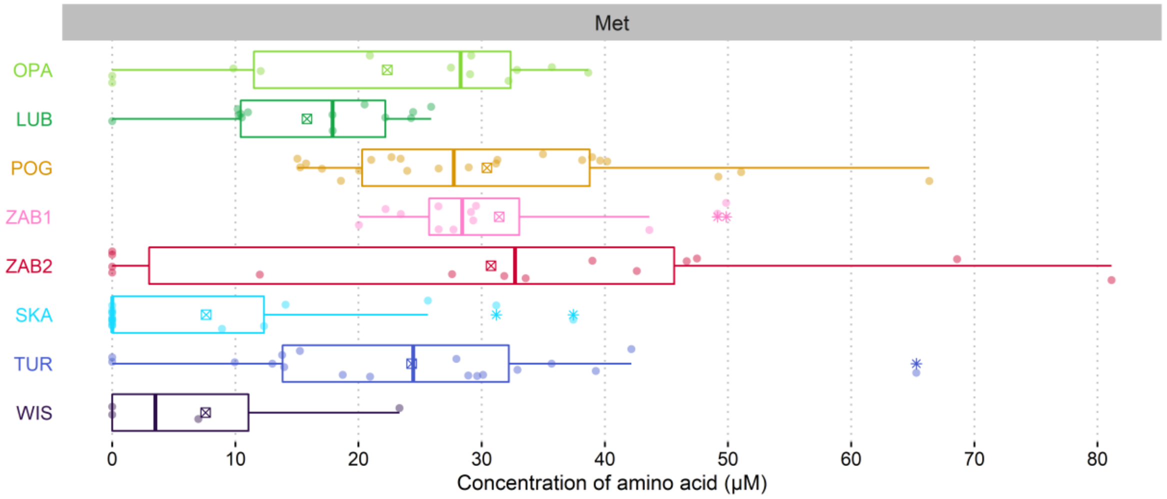
Met
OPA
LUB
POG
ZAB1
ZAB2
SKA
TUR
WIS
0
10
20
30
40
50
60
70
80
Concentration of amino acid (µM)

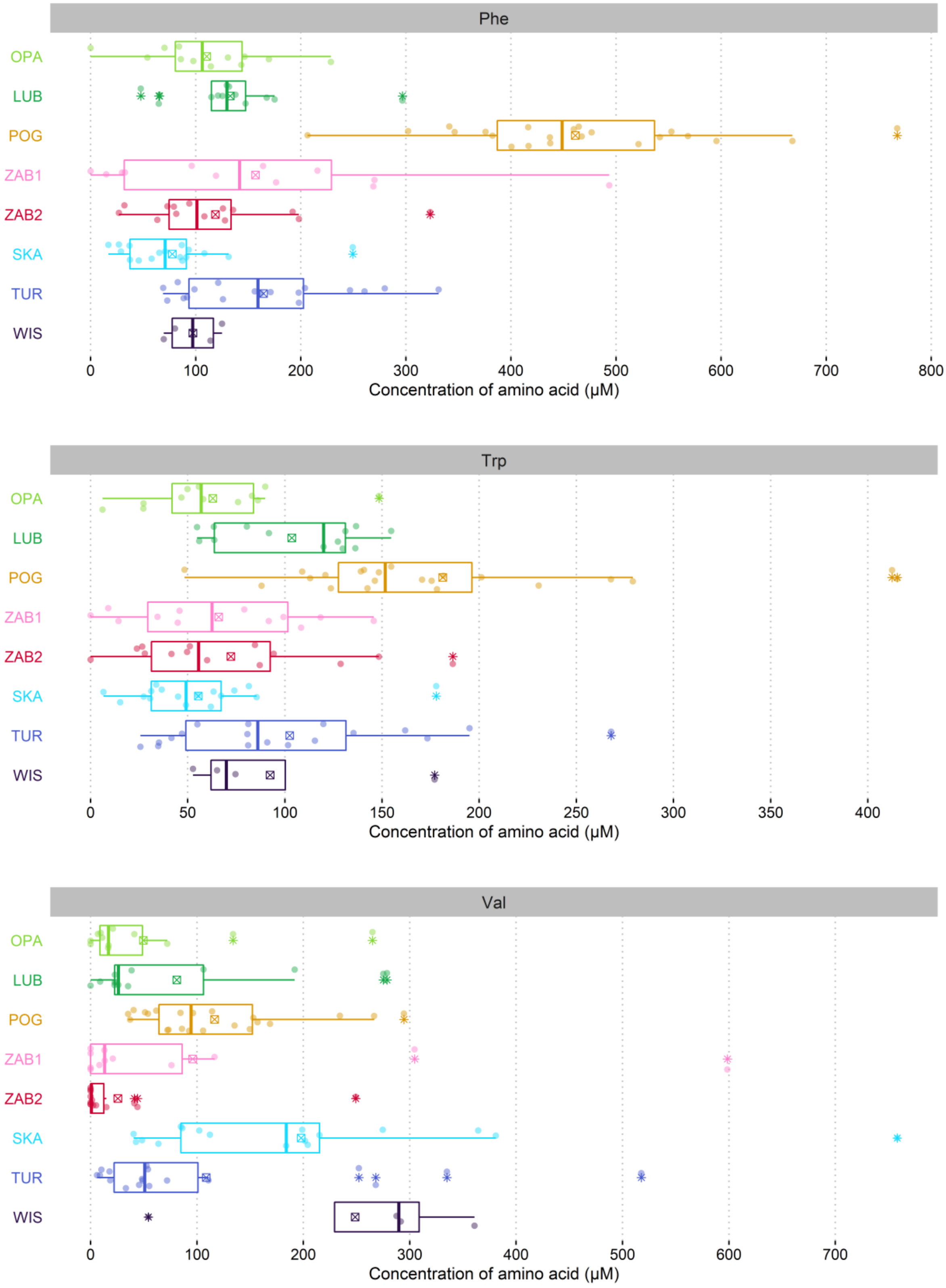
Phe
OPA
LUB
POG
ZAB1
ZAB2
SKA
TUR
WIS
0
100
200
300
400
500
600
700
800
Concentration of amino acid (μM)
Trp
OPA
LUB
POG
ZAB1
ZAB2
SKA
TUR
WIS
0
50
100
150
200
250
300
350
400
Concentration of amino acid (μM)
Val
OPA
LUB
POG
ZAB1
ZAB2
SKA
TUR
WIS
0
100
200
300
400
500
600
700
Concentration of amino acid (μM)

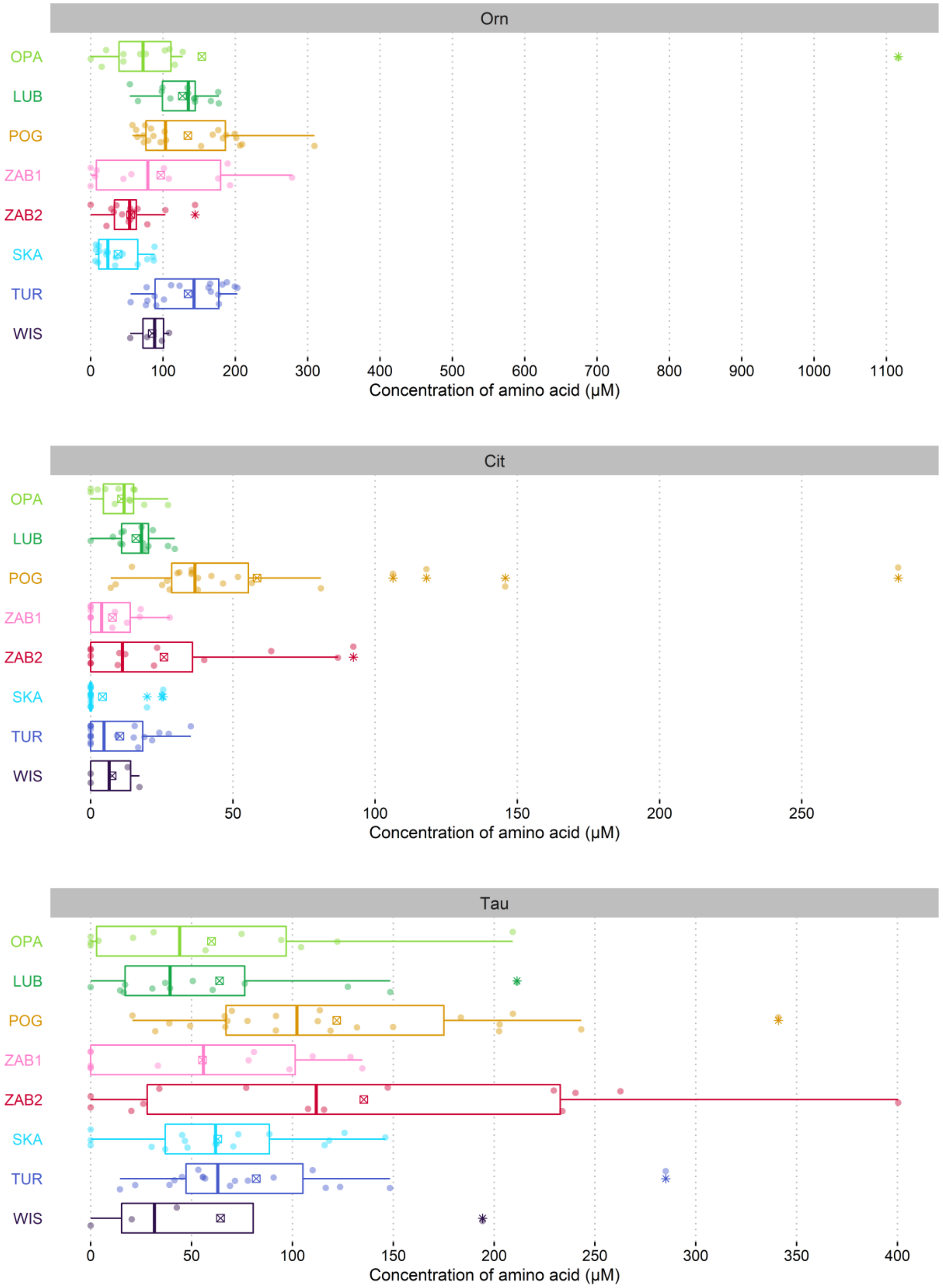
Orn
OPA
LUB
POG
ZAB1
ZAB2
SKA
TUR
WIS
0
100
200
300
400
500
600
700
800
900
1000
1100
Concentration of amino acid (μM)
Cit
OPA
LUB
POG
ZAB1
ZAB2
SKA
TUR
WIS
0
50
100
150
200
250
Concentration of amino acid (μM)
Tau
OPA
LUB
POG
ZAB1
ZAB2
SKA
TUR
WIS
0
50
100
150
200
250
300
350
400
Concentration of amino acid (μM)

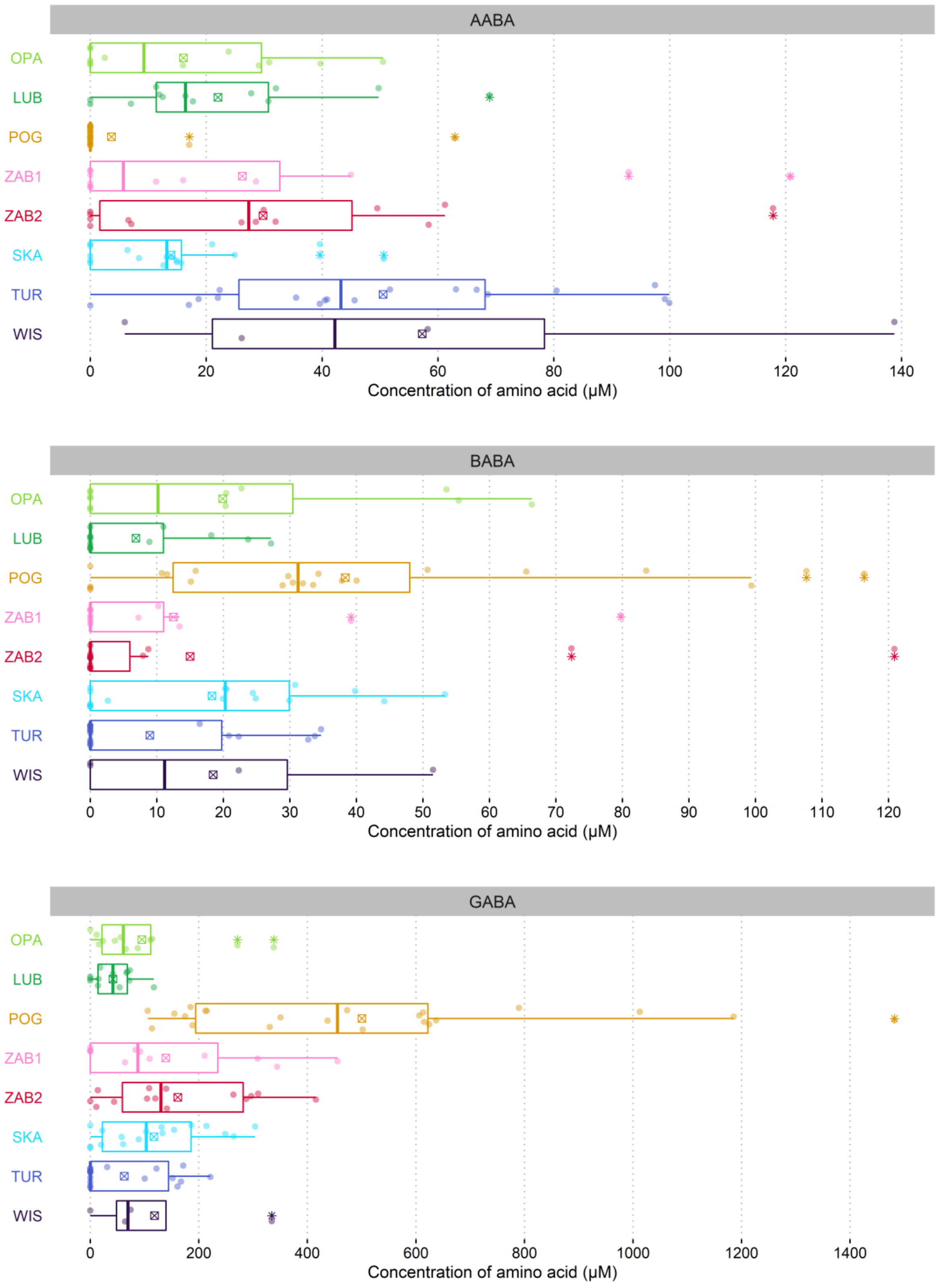
AABA
OPA
LUB
POG
ZAB1
ZAB2
SKA
TUR
WIS
0
20
40
60
80
100
120
140
Concentration of amino acid (μM)
BABA
OPA
LUB
POG
ZAB1
ZAB2
SKA
TUR
WIS
0
10
20
30
40
50
60
70
80
90
100
110
120
Concentration of amino acid (μM)
GABA
OPA
LUB
POG
ZAB1
ZAB2
SKA
TUR
WIS
0
200
400
600
800
1000
1200
1400
Concentration of amino acid (μM)

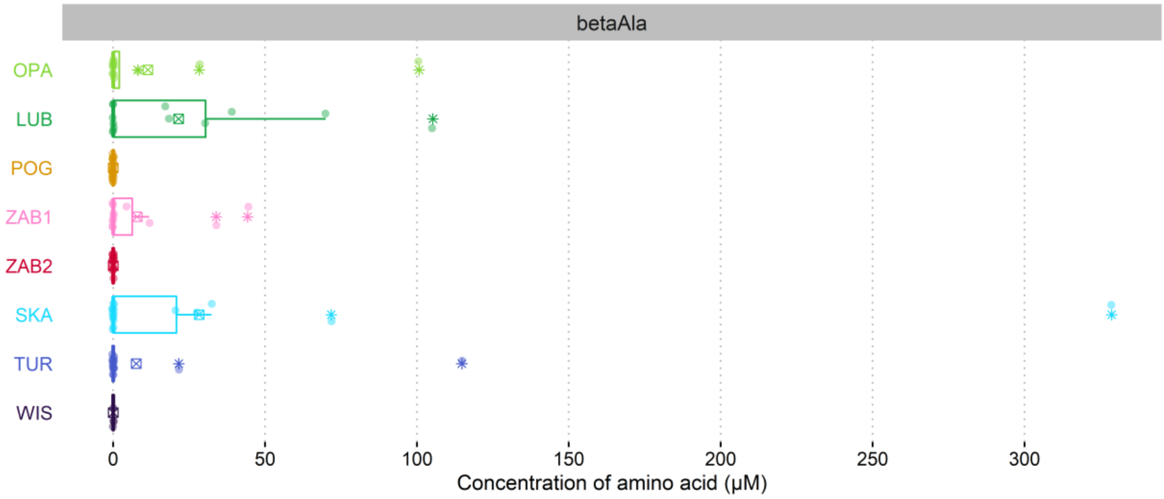

betaAla
OPA
LUB
POG
ZAB1
ZAB2
SKA
TUR
WIS
0
50
100
150
200
250
300
Concentration of amino acid (μM)

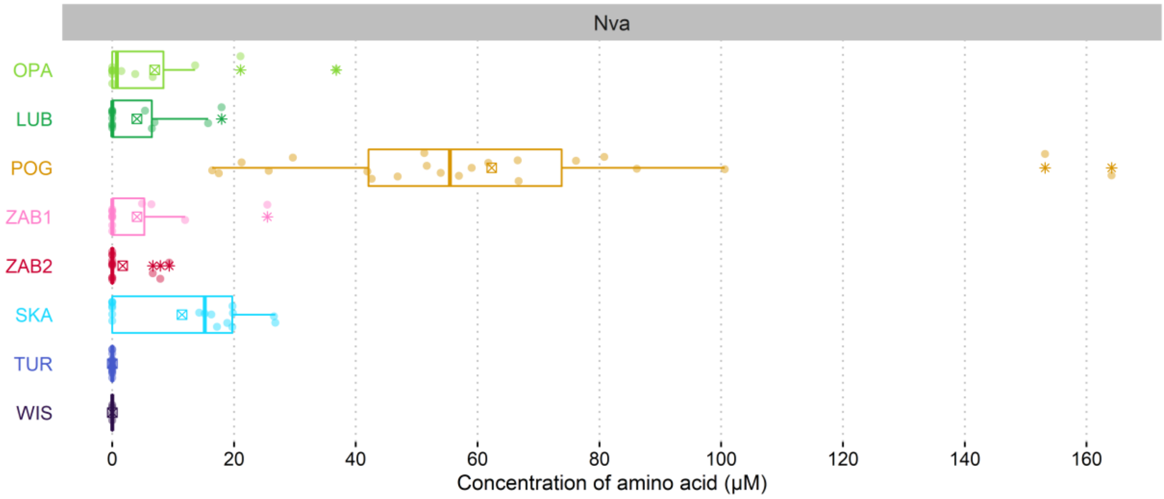

Nva
OPA
LUB
POG
ZAB1
ZAB2
SKA
TUR
WIS
0
20
40
60
80
100
120
140
160
Concentration of amino acid (μM)

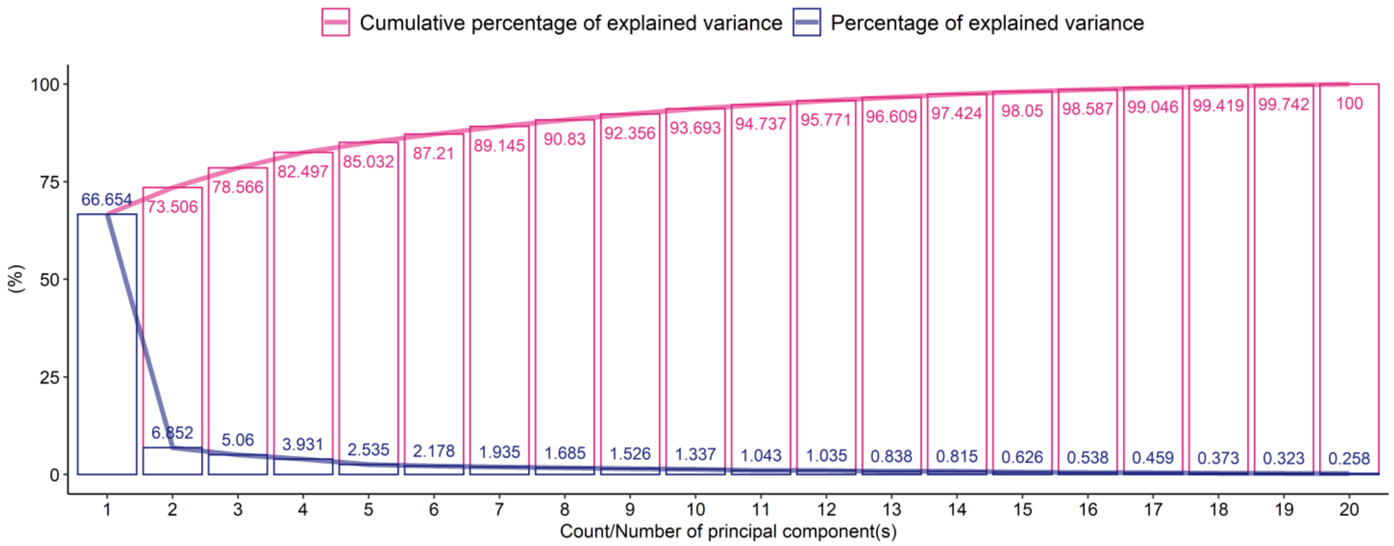

Figure S2. Scree plot showing the proportion of explained variance by the principal components.

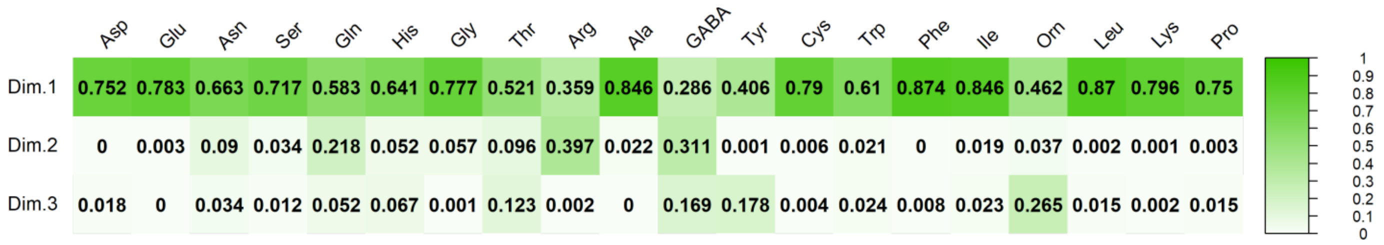

Figure S3. Cos² for the amino acids selected as active variables in the principal component analysis model, representing the quality of representation for variables on the factor map (Dim1-3).

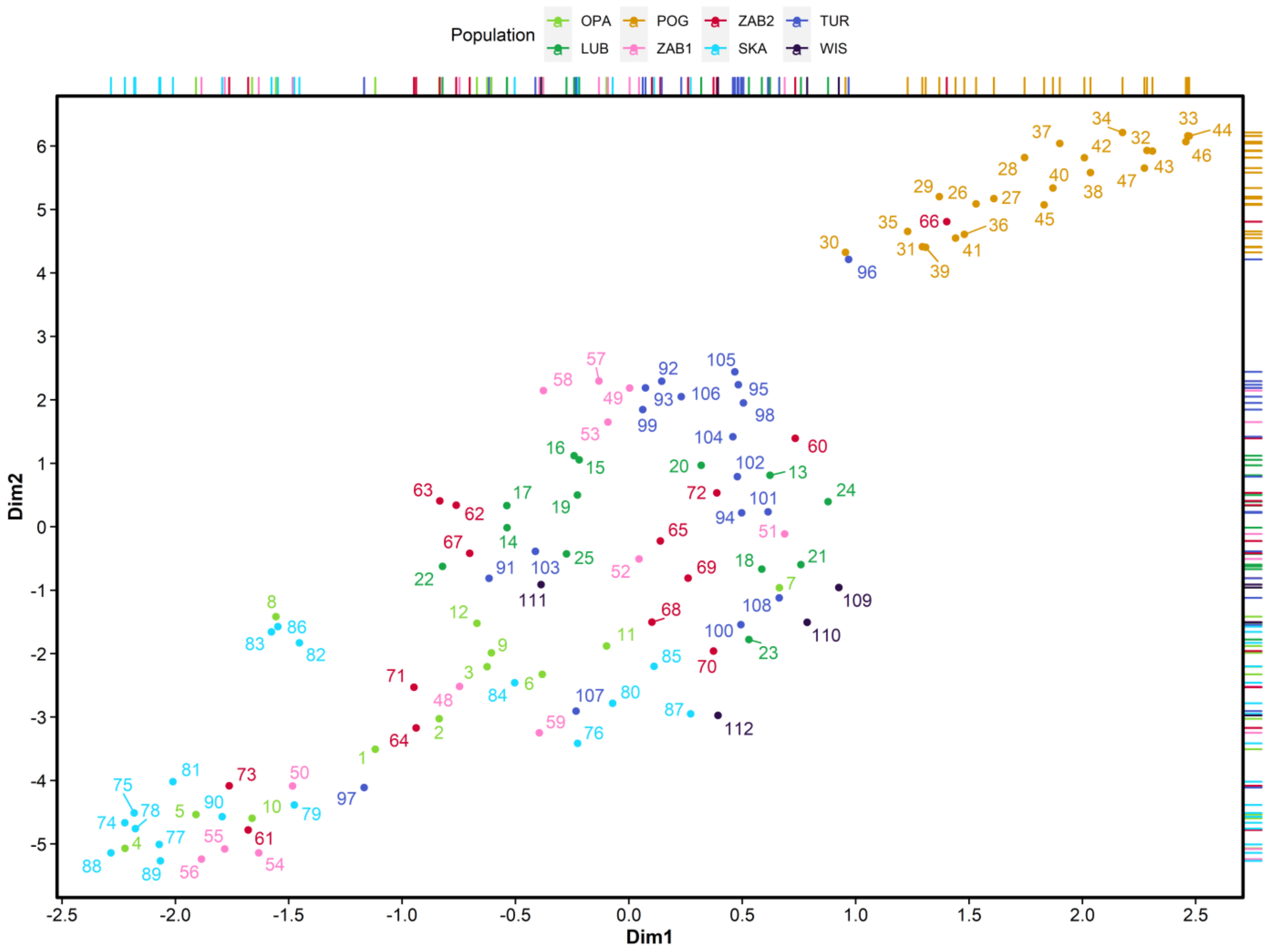

Figure S4. Uniform manifold approximation and projection of all amino acids in *Neottia ovata* populations, except for β-Ala. Individuals (populations) are color-coded and labeled with a number corresponding to Id used in Table S3.
